# Decoding CO_2_ Adsorption Modes and CO Yield Variation on TM–N_4_ Single-Atom Catalysts via Constant-Potential Computation and Machine-Learning Investigation

**DOI:** 10.1021/jacs.6c13568

**Published:** 2026-09-23

**Authors:** Linguo Lu, Weibin Chen, Jingsong Huang, Ian Street, Bobby G. Sumpter, Alexey Serov, Robert L. Sacci, Gabriel M. Veith, William E. Mustain, Ju Li, Zhongfang Chen

**Affiliations:** † Department of Physics, 19855 University of Puerto Rico, Rio Piedras, San Juan, Puerto Rico 00931, United States; ‡ Department of Nuclear Science and Engineering, Massachusetts Institute of Technology, 77 Massachusetts Avenue, Cambridge, Massachusetts 02139, United States; § Center for Nanophase Materials Sciences, 6146 Oak Ridge National Laboratory, Oak Ridge, Tennessee 37831, United States; ∥ Department of Chemical Engineering, 2629 University of South Carolina, Columbia, South Carolina 29208, United States; ⊥ Electrification and Energy Infrastructure Division, Oak Ridge National Laboratory, Oak Ridge, Tennessee 37831, United States; # Chemical Sciences Division, Oak Ridge National Laboratory, Oak Ridge, Tennessee 37831, United States; ∇ Department of Nuclear Science and Engineering and Department of Materials Science and Engineering, 2167 Massachusetts Institute of Technology, Cambridge, Massachusetts 02139, United States; ○ Department of Chemistry, 2310 University of Puerto Rico, Rio Piedras, San Juan, Puerto Rico 00931, United States

## Abstract

Transition metal-based TM–N_4_ single-atom catalysts (SACs) are promising materials for electrochemical CO_2_ reduction reactions (CO_2_RR) toward CO formation. In this study, we employed the constant-potential method to investigate CO_2_ adsorption and reduction on 10 TM–N_4_ SACs (TM = Sc–Zn), with a particular focus on Ni–N_4_. We first evaluated the CO_2_ adsorption mode on the TM active site as a function of the applied electrode potential, *U*. Landau free energy analysis revealed a transition in CO_2_ adsorption mode from physisorption (*D*_∞*h*_–*D*_2*h*_) to chemisorption (*C*_2*v*_–*C*_2*v*_) at a critical electrode potential, referred to as the “transorption” potential (*U*_trans_). Walsh diagram and machine-learning analyses revealed significant π*-orbital splitting and an unusual σ back-donation governing *U*_trans_. We then examined the CO_2_RR processes by considering both adsorption modes of CO_2_. The transition from CO_2_ physisorption to chemisorption modes leads to a shift in the rate-limiting step from CO_2_ hydrogenation to CO desorption, the emergence of new active sites for competing hydrogen evolution reactions, and a decrease in electron transfer rates at more negative potentials. Taken together, these observations align with the experimental CO yield profile under increasingly negative potentials, capturing key performance trends of rise, plateau, and decline. Notably, the calculated *U*_trans_ values are consistent with the applied potentials at which CO yield declines for five SACs for which experimental data are available (Mn, Fe, Ni, Cu, and Zn). Thus, this work establishes *U*_trans_ as a novel predictive parameter for CO yield performance and offers valuable guidance for the design of advanced SACs in future studies.

## Graphical abstract

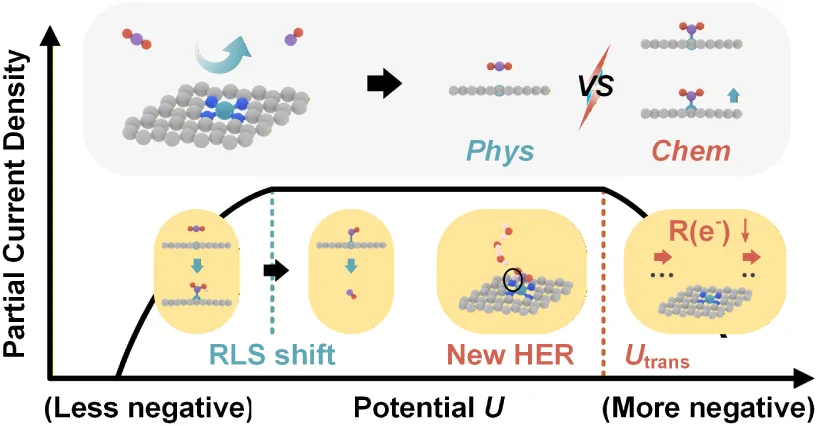

## Introduction

With escalating global energy demand and the urgent need to mitigate CO_2_ emissions, converting CO_2_ into value-added chemicals presents a viable and sustainable approach for carbon recycling.^1–6^ Among available strategies, the electrochemical CO_2_ reduction reaction (CO_2_RR) provides an efficient and cost-effective means of harnessing electrical energy to transform CO_2_ into useful products such as CO, HCOOH, CH_2_O, CH_3_OH, and CH_4_. Since copper was first recognized as an effective CO_2_RR electrocatalyst,^7^ intensive efforts have broadened the search to other transition metals (TMs) to improve reaction activity and selectivity.^8–10^ More recently, single-atom catalysts (SACs) have drawn increasing attention due to their well-defined active sites, near-unity metal utilization efficiency, and highly tunable electronic structures.^11–14^

Among single-atom architectures, TM–N_4_ SACs, in which each TM atom is coordinated by four N atoms within a graphene matrix, have emerged as promising platforms for CO_2_RR-to-CO conversion.^15–18^ Progress has been propelled by synergistic theory-experiment advances: the earliest density functional theory (DFT) study identified Rh–N_4_ as a viable CO_2_RR catalyst,^19^ and subsequent experiments confirmed the catalytic potential of TM–N_4_ SACs, highlighting Ni–N_4_ for its high CO selectivity.^20–25^ Despite many DFT investigations into Ni–N_4_ behavior, significant gaps remain in mechanistic understanding and in reconciling theoretical predictions with experimental performance metrics, particularly regarding potential-dependent adsorption modes and rate-limiting steps (RLS).

A central challenge is to resolve the CO_2_RR mechanism on Ni–N_4_ SAC, specifically, the operative CO_2_ adsorption mode and the true RLS.^16^ Most prior theoretical studies utilized the constant charge (CC) approach in conjunction with the computational hydrogen electrode (CHE) model,^26–28^ which typically favors CO_2_ physisorption on Ni–N_4_ and assigns the *COOH formation to a coupled electron–proton transfer (CEPT: * + CO_2_ + H^+^ + e^–^ → *COOH) pathway.^29–32^ However, the CC/CHE framework couples electron transfer (ET) and proton transfer (PT) on a physisorbed CO_2_ state and therefore neglects the CO_2_ chemisorption and the stepwise, decoupled ET and PT route (DEPT: * + CO_2_ → *CO_2_; *CO_2_ + H^+^ + e^–^ → *COOH).^8,14,33–37^ Together with the fixed Fermi level in the CC framework, the treatment overestimates the free-energy change for *COOH formation (∼1.8 eV) and artificially promotes it as the RLS.

In contrast to the widely used CC method, the constant potential (CP) approach explicitly captures potential-dependent Fermi-level shifts and interfacial electron transfer under working electrochemical conditions.^17,38–42^ Early CP studies on Ni–N_4_ suggested that the reaction initiates with CO_2_ physisorption, and *COOH formation is identified as the RLS.^38,43^ In contrast, more recent research indicates that cation effects can shift the CO_2_ adsorption mode toward chemisorption, enabling a DEPT pathway.^44^ On a Fe–N_4_ SAC, both the adsorption mode and the RLS were found to vary with applied potential and ligand environment.^45^ These divergent findings indicate that the CO_2_RR mechanism is strongly potential-dependent, underscoring the need for a more consistent mechanistic picture for Ni–N_4_ and related SACs.

Another challenge is to reconcile DFT predictions with experiments to achieve a comprehensive understanding of CO_2_RR performance. Two metrics are commonly used: CO selectivity (based on Faradaic efficiency) and CO yield (derived from the CO partial current density, PCD). Note that their optima often occur at different electrode potentials. For example, in the case of Ni–N_4_ SAC, peak selectivity typically appears near ∼−0.7 V vs. reversible hydrogen electrode (RHE), whereas the maximum CO yield spans a broader potential plateau from ∼−1.0 V to ∼−1.6 V vs. RHE. While the absolute CO yield scales with the number of active sites, the potential-dependent trend, such as the rise, the plateau, and overall shape of the yield curve, tends to be characteristic of a given TM. Although recent DFT studies have provided valuable insights into CO-H_2_ selectivity,^46^ the variation of CO yield with potential has received far less attention. Therefore, a deeper understanding of CO yield is essential to capture catalytic performance and to guide the rational design of improved SACs.

In this work, using the fully converged constant-potential (FCP) method within the grand canonical ensemble, we investigated how applied electrode potential governs CO_2_ adsorption mode transitions and CO yield on 3d TM–N_4_ SACs. As depicted for Ni–N_4_ in Figure 1, we found that the applied potential, *U*, tunes the competition between CO_2_ physisorption and chemisorption. Walsh diagrams, together with machine-learning (ML) analysis, elucidate the accompanying electronic structure evolution. We introduced the concept of “transorption” potential (*U*_trans_) that marks the crossover at which CO_2_ chemisorption becomes thermodynamically more favorable than physisorption. To rationalize the experimentally observed CO yield profile (Figure S1), we identified three key factors: a shift of the rate-limiting step (*COOH formation → CO release), the emergence of new HER active sites after chemisorption, and a decrease in electron transfer rates at high overpotentials. Notably, the calculated *U*_trans_ values coincide with the experimental potentials at which the CO yield begins to decline. Leveraging this correlation, we propose *U*_trans_ as a DFT-derived descriptor for the design and screening of advanced SACs.

**Figure 1:**
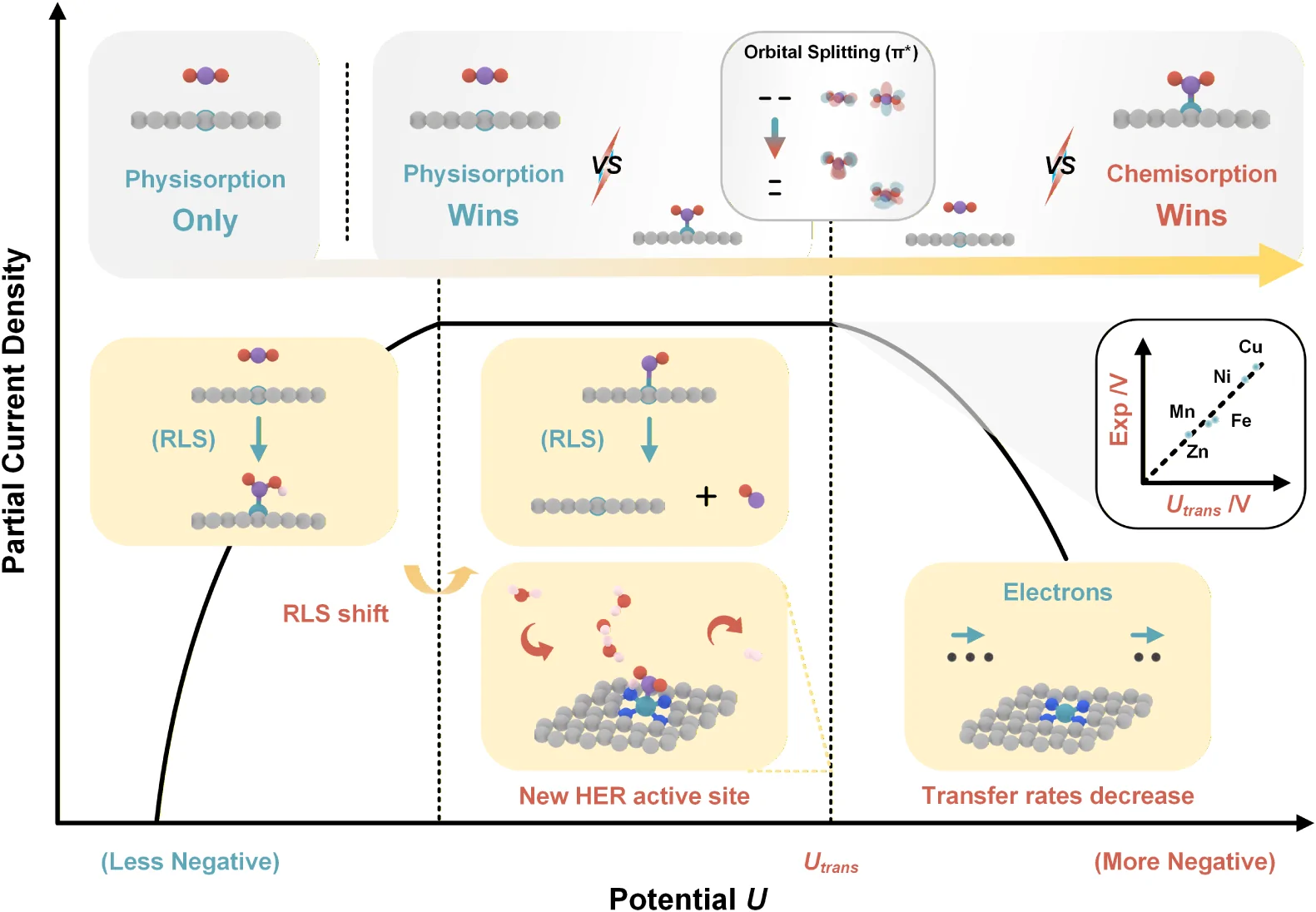
Schematics of competing CO_2_ adsorption modes and CO partial current density (PCD) as a function of electrode potential *U*. The PCD curve exhibits trends of rise, plateau, and decline as the applied potential *U* shifts from less negative to more negative values. These trends are driven by the shift in RLS, the emergence of new active sites for competing hydrogen evolution reactions (HER), and the decrease in electron transfer rates. The observed trends were extracted from a comprehensive experimental data set, which reveals the sequential rise, plateau, and eventual decline in performance (Figure S1). The C atoms in the graphene substrate are colored gray, while those in CO_2_ are rendered in purple. The N, O, H, and Ni atoms are shown in blue, red, pink, and cyan, respectively. Electrons are shown as black dots.

## Computational Methods

Density functional theory (DFT) calculations were conducted using the Vienna Ab initio Simulation Package (VASP).^47,48^ The interaction between ion cores and valence electrons was described using the projector-augmented wave (PAW) method.^49^ Exchange-correlation effects were treated using the Perdew–Burke–Ernzerhof (PBE) functional within the generalized gradient approximation (GGA).^50^ To improve accuracy for 3d TMs, spin polarization was included and on-site Coulomb interactions were treated with the Hubbard U corrections (values in Table S1).^51–53^ As TM–N_4_ SACs exhibit potential-dependent spin reorganization and metastable spin manifolds,^54,55^ multiple initial magnetic-moment configurations (Tables S2–S7) were examined for each reaction intermediate. The energetics reported in this work correspond to the lowest-energy state identified among the spin configuration examinations. Additional occupation-matrix-controlled (OMC) calculations^56^ using different initial Ni 3*d*-orbital occupations further confirmed the robustness of the identified electronic states over the investigated potential range (Table S8).

The catalyst models were constructed from a (5 × 5 × 1) graphene supercell (Figure S2) with a Gamma-centered 3 × 3 × 1 *k*-points mesh for Brillouin zone sampling during structural optimizations. The plane-wave energy cutoff was set to 550 eV, and the convergence criteria were 1 × 10^–5^ eV for electronic energy and 0.01 eV/Å for atomic forces. The lattice vector along the *z*-axis was fixed at 25 Å to ensure a large vacuum, thereby eliminating spurious interactions between periodic images. The DFT-D3 method, incorporating Becke–Johnson damping, was employed to account for van der Waals (vdW) forces, a technique commonly used in recent CO_2_RR studies.^57,58^ The “accurate” precision setting was selected to reduce wrap-around errors, improving numerical stability. To accelerate convergence, we employed Gaussian smearing with a smearing width of 0.1 eV, which enhances numerical stability and ensures smooth electronic convergence for metallic systems.

The catalytic reactions under applied potentials were modeled with the FCP method within the grand canonical ensemble interfaced with VASP.^59^ In this framework, the electron number *N_e_* is optimized at each ionic step so that the calculated Fermi level *E*_f_ converges to the prescribed electron chemical potential *μ_e_*. For each trial *N_e_*, VASP performs a conventional fixed-*N_e_* self-consistent calculation, and *N_e_* is subsequently updated using the polynomial-fitted, damped Newton scheme until the target potential is reached. This procedure effectively allows electron exchange with an external reservoir under constant-potential conditions.

The electron chemical potential is defined as

$$\mu_{e}=\mu -\left|e\right|U$$ 1

where *μ_e_*, *μ*, and *U* are the chemical potential of electrons, the work function of the standard hydrogen electrode (SHE), and the value of applied potential referenced to SHE, respectively. In this calculation, the absolute SHE work function (*μ*) value is taken as −4.44 eV.^60–62^ All applied potentials reported in this manuscript are referenced to SHE, unless otherwise specified. Experimental potentials reported versus RHE were converted to SHE using the relevant pH values. Furthermore, continuum solvation and electrolyte screening were included in the FCP calculations using the VASPsol implicit electrolyte model,^63,64^ in which the dielectric constant was set as 78.4 for the water solvent. Although more sophisticated nonlinear/nonlocal electrolyte models may refine absolute values, the adsorption-mode crossover is expected to remain qualitatively robust because all compared states are treated within the same CP-solvated framework.

Due to the grand-canonical treatment, the relevant energy descriptor is the Landau free energy (Ω, also known as the grand potential). The Ω values of reactants, intermediates, and products in the CO_2_RR processes were calculated as

2 $$\Omega =E_{DFT}+ZPE-TS-\mu_{e}N+E_{vib}+E_{tran}+E_{rot}$$

*E_DFT_* represents the DFT-calculated total energy, *ZPE* is the zero-point energy, *TS* is the entropic term at room temperature (298.15 K). *N* denotes the net excess electron number of the entire system relative to its charge-neutral reference. At each applied potential, *N* is adjusted self-consistently until the Fermi level matches the imposed electron chemical potential, *μ_e_*, of an electron reservoir. Note that *N* is a global property of the calculated system rather than a local adsorbate charge obtained from Bader or other charge-partitioning analyses. The *μ_e_N* term accounts for the energetic contribution associated with electron exchange between the simulated cell and the electron reservoir. The *E_vib_*, *E_tran_*, and *E_rot_* terms represent the thermal corrections associated with the vibrational, translational, and rotational energy contributions for free molecules; for adsorbates only vibrational contributions were considered. The second, third, and fifth to seventh correction terms were postprocessed with the VASP toolkit.^65^ To reflect varying electrolyte pH values in experiments, we added the correction term ΔΩ*_pH_* = *RT*·In (10) × *pH* to electrochemical steps involving proton–electron transfers, where *R* is the gas constant.

The CO_2_ adsorption and reduction sequence can be represented as follows:

(*e1)+CO2+etrans,1→C*O2(e2) 3a

C*O2(e2)+H++etrans,2→C*OOH(e3) 3b

C*OOH(e3)+H++etrans,3→C*O(e4)+H2O 3c

C*O(e4)+etrans,4→(*e1)+CO 3d

where *CO_2_, *COOH, and *CO denote the intermediates adsorbed on the active site *, *e_i_* (*i* = 1–4) denotes the state-dependent electron population of the electrode/adsorbate system at the applied potential *U*, whereas *e_trans_*_,_ *_i_* stand for the number of electrons exchanged with the external electron reservoir during the elementary step *i*. *H* ^+^ represents the proton participating in the corresponding protonation step. Electron-number balance between successive states and the electron reservoir is given by

4a $$e_{2}=e_{1}+16+e_{trans,1}$$

4b $$e_{3}=e_{2}+e_{trans,2}$$

4c $$e_{4}=e_{3}+e_{trans,3}-8$$

4d $$e_{1}=e_{4}+e_{trans,4}-10$$

The corresponding Landau adsorption free energies at potential *U* are identified as

5a $$\Delta \Omega_{*CO_{2}}=\Omega_{*CO_{2}}-\Omega_{CO_{2}}-\Omega_{*}$$

5b $$\Delta \Omega_{*COOH}=\Omega_{*COOH}-\Omega_{*CO_{2}}-\frac{1}{2}\Omega_{H_{2}}+eU$$

5c $$\Delta \Omega_{*CO}=\Omega_{*CO}+\Omega_{H_{2}O}-\Omega_{*COOH}-\frac{1}{2}\Omega_{H_{2}}+eU$$

5d $$\Delta \Omega_{CO}=\Omega_{*}+\Omega_{CO}-\Omega_{*CO}$$

where $\Omega_{CO_{2}}$ , $\Omega_{H_{2}}$ , $\Omega_{H_{2}O}$ , and Ω*_CO_* represent the Landau free energies of the free gas-phase molecules, while Ω_*_, $\Omega_{*CO_{2}}$ , $\Omega_{*COOH}$ , and Ω_*_ *_CO_* denote those of the active site and adsorbed intermediates.

To evaluate the thermal stability of intermediates during the CO_2_RR processes, *ab initio* molecular dynamics (AIMD) simulations were conducted in the canonical (NVT) ensemble with a Nose–Hoover thermostat.^66,67^ The system temperature was maintained at 300 K, corresponding to room temperature relevant for experimental conditions. Each trajectory ran for 10 ps with a time step of 1 fs, providing adequate sampling of dynamic fluctuations between intermediates and active centers (TM–N_4_). Explicit water molecules were not included in AIMD calculations since their incorporation would substantially increase the computational cost; consequently, these simulations are intended to reveal qualitative trends in the finite-temperature stability, rather than to quantitatively resolve specific solvent-mediated interactions.

The AIMD calculations were performed at fixed electron number, with the total electron number initialized from the corresponding CP-DFT calculation at *U*_trans_ and subsequently held constant during the trajectory. These simulations were used only to evaluate the finite-temperature structural stability of the CP-derived chemisorbed states and should not be interpreted as fully grand-canonical dynamics. As an *a posteriori* check, the CO_2_-chemisorbed state on Ni–N_4_ was examined by evaluating the electrode potentials inferred from snapshots along the AIMD trajectory, which showed only minor deviations from the target value (Figure S3). This treatment is also consistent with related fixed-charge strategies previously used to approximate CP energetics in electrocatalysis.^68–70^

Microkinetic simulations were performed with the MKMCXX program to evaluate the CO/H_2_ selectivity.^71^ The simulations utilized a mean-field, well-mixed surface reactor model and did not include explicit mass-transport terms. Fixed reservoir conditions were employed, in which reactants (CO_2_ and H_2_O) were treated as constant external reservoirs. At t = 0, the surface was initialized with one adsorbed CO_2_ species and four vacant sites, consistent with the site normalization used in the MKMCXX input. The coupled ordinary differential equations governing surface coverages were integrated using the deterministic SEQUENCERUN solver, with both the relative and absolute tolerances set to 10^–8^ (dimensionless).

Reaction rate constants were evaluated within transition-state theory using the Eyring equation under the harmonic approximation, based on the CI-NEB barriers and free-energy corrections described above. All the transition states needed in the microkinetic simulations were determined using the climbing image nudged elastic band (CI-NEB) method.^72^ For CI-NEB calculations, an initial water-containing configuration was generated using Packmol by placing 25 H_2_O molecules around the catalytic surface.^73^ The resulting structure was then equilibrated via AIMD for 5 ps to obtain a more representative solvation environment. After equilibration, 13 H_2_O molecules near the active site were retained to construct the initial and final states with explicit solvation for the subsequent CI-NEB calculations. Following structural relaxation of the initial and final states, CI-NEB calculations along the reaction pathway were carried out using a quasi-Newton algorithm with a 5.0 eV/Å^2^ spring constant. To balance computational cost and accuracy, the force convergence threshold was set to 0.05 eV/Å, while all other convergence settings were kept identical to those used in the geometry optimizations.

To quantify material descriptors controlling *U*_trans_, we employed an ML workflow based on the Sure Independence Screening and Sparsifying Operator (SISSO),^74^ a compressed sensing symbolic regression method used to identify the physically interpretable descriptors governing the crossover from CO_2_ physisorption to chemisorption. The initial feature pool consisted of 21 primary descriptors spanning atomic properties, bulk electronic characteristics, and surface orbital features. Models were constructed in both one-dimensional (1D) and two-dimensional (2D) descriptor spaces with a maximum expression complexity of 5. Leave-one-out cross-validation (LOOCV) was performed to assess model robustness.

## Results and Discussion

### Physisorption and Chemisorption under Constant Potential

To predict CO_2_ adsorption on TM–N_4_ SACs under varying applied potentials, we first analyzed the symmetry characteristics of the adsorbate-catalyst ensemble. Figure 2a illustrates the possible adsorption modes, and Figure 2b summarizes the corresponding orbital interactions between CO_2_ and TM *d*-orbitals. Free CO_2_ is linear with *D*_∞*h*_ symmetry, while a pristine, square-planar TM–N_4_ center adopts *D*_2*h*_ symmetry. In the physisorption state, CO_2_ remains linear and interacts with the TM center mainly through van der Waals forces with negligible charge transfer, essentially preserving the *D*_∞*h*_ (CO_2_) and *D*_2*h*_ (TM–N_4_) symmetries. However, chemisorbed CO_2_ assumes bent *C*_2*v*_ symmetry, activating symmetry-allowed overlap with metal *d*-orbitals and enabling charge transfer. Two possible chemisorption geometries then arise: (i) planar, in which the TM atom remains nearly coplanar with the N_4_ ligand (site symmetry ∼*D*_2*h*_); and (ii) pyramidal, in which the TM atom protrudes from the N_4_ plane toward CO_2_, lowering the site symmetry to *C*_2*v*_, and increasing orbital overlap. Therefore, potential-dependent CO_2_ adsorption can be viewed as a competition between one physisorption configuration and two distinct chemisorption configurations (planar vs pyramidal), whose relative stabilities are governed by symmetry and metal-CO_2_ orbital matching.

**Figure 2:**
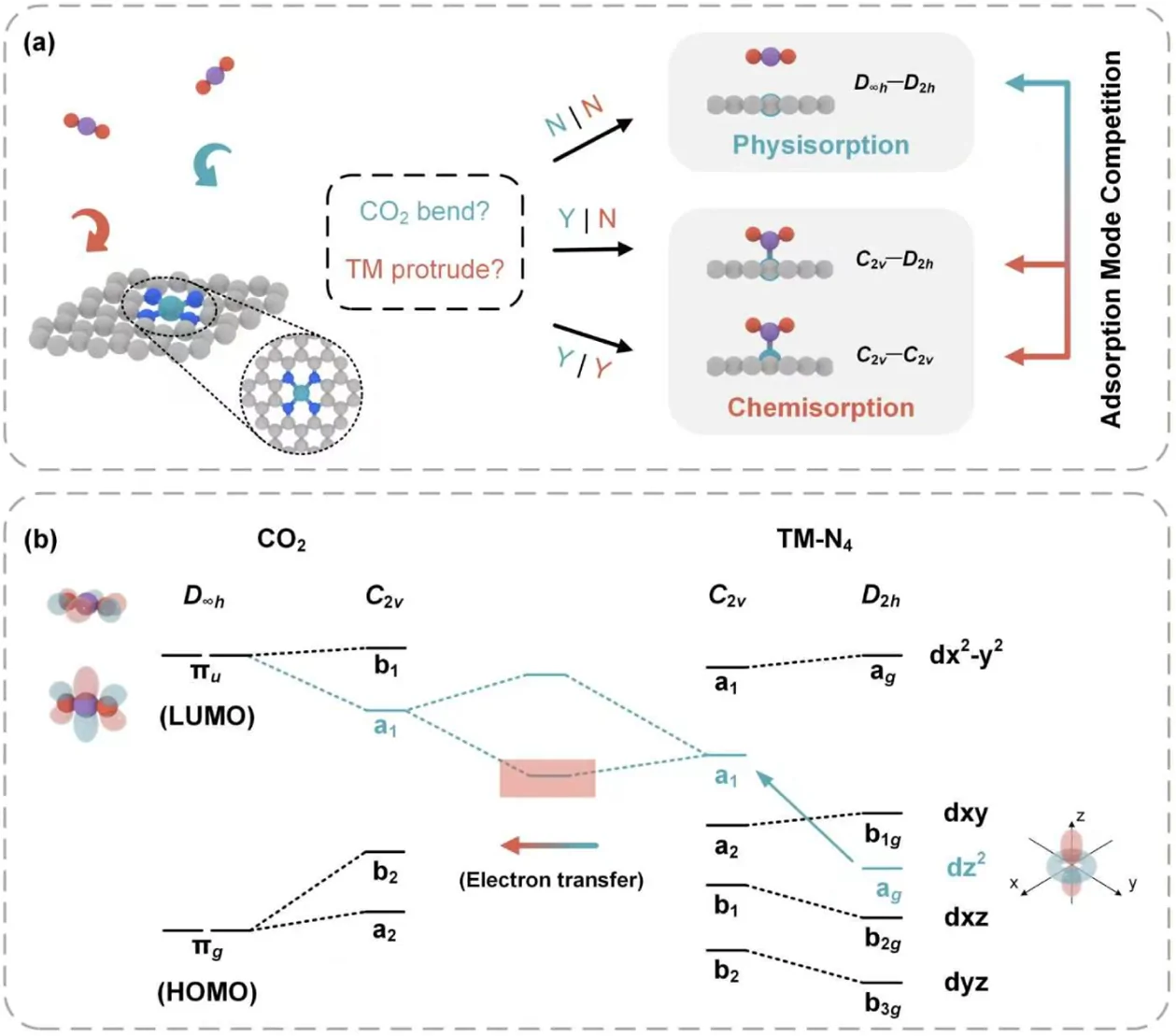
(a) Competition of CO_2_ adsorption modes on TM–N_4_ SAC, including one physisorption and two chemisorptions, depending on CO_2_ bending and TM protrusion. (b) Orbital energy level diagrams of CO_2_ and TM–N_4_ SAC, illustrating how symmetry changes affect orbital splitting and interaction during adsorption. The C atoms in the graphene substrate are colored gray, while those in CO_2_ are rendered in purple. The N, O, and Ni atoms are shown in blue, red, and cyan, respectively.

We next systematically investigated CO_2_ adsorption modes across 3d TM–N_4_ SACs under varying applied potentials, using Ni–N_4_ SAC as a representative case due to its experimentally reported highest CO_2_RR performance.^20–25^ Beyond physisorption, we considered four possible chemisorption configurations, namely, η^1^-C, η^1^-O, η^2^-C,O, and η^2^-O,O, as shown in Figure S4. The η notation is used to describe the adsorption mode of CO_2_ on the TM center, where η^1^ indicates that only one atom is connected to the active site, and η^2^ indicates that two atoms are connected to the active site. For Ni–N_4_, η^1^-C is the only stable chemisorbed structure, corresponding to the bent *C*_2*v*_-type state predicted from symmetry considerations, whereas the other three motifs converge to the η^1^-C or physisorption upon geometry optimization. To ensure the stability of the lowest-energy state and avoid the metastable spin manifolds, we tested multiple initial magnetic moment configurations for each adsorption mode (Tables S2–S7) and adopted the results corresponding to the lowest energy state for the subsequent analysis. Note that though metastable manifolds exist, the lowest-energy-state ordering still preserves the physisorption → chemisorption crossover and does not change the reported *U*_trans_ trend.

To clarify the CO_2_ adsorption landscape, we examined the potential-dependent Landau free energy profiles on Ni–N_4_ SAC under applied potentials of *U* = −1.5, −2.0, and −2.5 V (vs SHE) (Figure 3a). To generate the physisorption and chemisorption curves, we began with two fully relaxed configurations: one corresponding to physisorption (where the CO_2_-TM distance is around 3 Å) and the other to chemisorption (with a CO_2_-TM distance of approximately 2 Å). We then calculated the adsorption energy profile as a function of CO_2_ adsorption height by constraining the z-coordinate of the C atom while relaxing all other degrees of freedom. This constrained-relaxation approach resulted in two distinct curves: the physisorption branch evolves from a weakly bound, nearly linear CO_2_ state at longer C–Ni distances, whereas the chemisorption branch originates from a bent, charge-transferred CO_2_ state at shorter distances.

**Figure 3:**
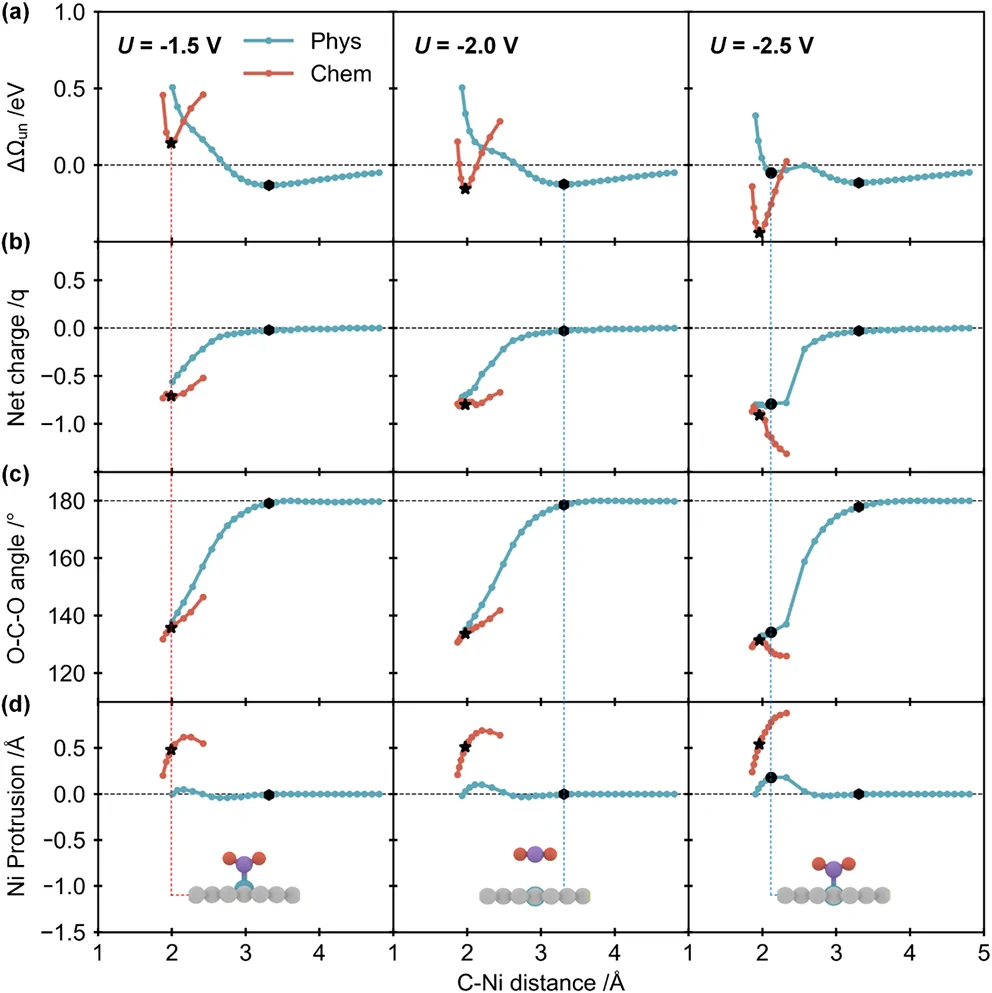
(a) Landau CO_2_ adsorption energy profiles (ΔΩ_un_), (b) net charge transfer to CO_2_, (c) O–C–O angle, and (d) Ni protrusion height as functions of the C–Ni distance for the physisorption (blue) and chemisorption (red) branches on Ni–N_4_ at *U* = −1.5, −2.0, and −2.5 V vs SHE. The distinct curve shapes arise from two different CO_2_ adsorption pathways: the physisorption branch evolves from a weakly bound, nearly linear CO_2_ state at long C–Ni distances, whereas the chemisorption branch corresponds to a bent, charge-transferred CO_2_ state with pronounced Ni out-of-plane distortion. At *U* = −2.5 V, a metastable chemisorption-like configuration appears at near ∼2.2 Å along the physisorption branch. Black symbols mark the local minima on each branch. ΔΩ_un_ denotes the adsorption energy without ZPE, entropy, or thermal corrections. The graphene C atoms are colored gray, the CO_2_ C atoms purple, and the N, O, and Ni atoms blue, red, and cyan, respectively.

Note that both physisorption and chemisorption branches exhibit distinct minima (Figure 3a): a physisorption minimum near ∼3 Å (marked as solid hexagon), corresponding to the *D*_∞*h*_–*D*_2*h*_ configuration, and a chemisorption minimum near ∼2 Å (solid asterisk), associated with the *C*_2*v*_–*C*_2*v*_ configuration (*vide infra* for further details). The physisorption minimum is largely unaffected by the applied potentials. In contrast, the chemisorption minimum stabilizes progressively with increasing overpotential (more negative potentials), transitioning from an unfavorable state at −1.5 V to a competitive state at −2.0 V, and finally becoming the global minimum at −2.5 V.

A notable feature arises at shorter C–Ni distances along the physisorption energy curve: a kink at −1.5 and −2.0 V that develops into a local minimum at −2.5 V (solid circle). This feature corresponds to an alternative metastable chemisorption-like configuration (*C*_2*v*_–*D*_2*h*_), in which CO_2_ becomes partially activated while the Ni–N_4_ unit remains essentially planar. Accordingly, at *U* = −2.5 V, the physisorption branch contains two local minima: a shallow minimum near ∼3 Å, corresponding to a weakly bound, nearly linear CO_2_ state with negligible charge transfer, and a second minimum near ∼2.2 Å, where CO_2_ is bent and partially charge-transferred.

Together, these trends reveal the intrinsic potential dependence of CO_2_ adsorption: physisorption dominates at less negative potentials, while chemisorption becomes thermodynamically preferred as the potential is more negative.

To elucidate the nature of these three energy minima, we examined the net charge transfer to CO_2_ and their geometric characteristics (Figure 3b–d). Note that the net charge shown for CO_2_ in Figure 3b represents a local charge-partitioning result for the adsorbate and should not be confused with the global excess electron count *N* in the Landau free energy expression. The physisorption minimum at ∼3 Å exhibits negligible charge transfer, a linear CO_2_, and a planar Ni–N_4_ motif, consistent with a weakly bound *D*_∞*h*_–*D*_2*h*_ configuration. At *U* = −2.5 V, a local minimum emerges along the physisorption curve at ∼2.2 Å, which shows moderate charge transfer and CO_2_ bending while the Ni–N_4_ unit remains essentially planar, indicating a *C*_2*v*_–*D*_2*h*_ chemisorption mode. In contrast, the minimum along the chemisorption curve exhibits the largest charge transfer, significant CO_2_ bending, and a distinct out-of-plane Ni displacement, characteristic of the *C*_2*v*_–*C*_2*v*_ chemisorption mode.

The enhanced stability of the *C*_2*v*_–*C*_2*v*_ chemisorption mode arises from improved symmetry-allowed overlap and energy alignment between the CO_2_ a_1_ orbital and the Ni $d_{z^{2}}$ orbital (Figure 2b). In second-order perturbation theory, better energy alignment lowers the electronic energy and strengthens the chemisorption interaction. Although pyramidalization of the Ni–N_4_ site incurs some strain penalty, the concurrent electronic stabilization due to stronger C–Ni interaction outweighs this cost, yielding a net thermodynamic gain for the CO_2_–Ni–N_4_ system. In addition, electron donation from Ni into the a_1_ orbital promotes additional bending, further reinforcing the chemisorption via a pseudo-Jahn–Teller effect.

To better understand how adsorption reshapes the CO_2_ electronic structures, we analyzed the Walsh diagrams (shown in Figures S5–S7) that track the evolution of orbital energies as the molecule bends. Prior work by Wang et al. examined orbital splitting in isolated CO_2_,^75^ whereas our analysis explicitly includes surface-molecule interactions and charge transfer. Among all the orbitals examined, only the a_1_ orbital stabilizes (lowers in energy) upon bending, consistent with a reduction in nodal planes from 3 to 2. As CO_2_ distortion increases, the degenerate π_u_ orbitals split into a higher-energy b_1_ orbital and multiple lower-energy a_1_-derived states (Figures S8–S13). These a_1_-derived states appear exclusively in chemisorbed configurations and span the Fermi level, enabling electron donation into CO_2_ that stabilizes adsorption.

Compared to the C_2*v*_–D_2*h*_ chemisorption found along the physisorption curve, the C_2*v*_–C_2*v*_ state on the chemisorption curve exhibits deeper a_1_ orbital penetration and stronger stabilization, driven by symmetry reduction of the Ni–N_4_ site. This pyramidal distortion shortens the C–Ni distances, enhances orbital overlap (Ni $d_{z^{2}}$ → CO_2_ *a*_1_), strengthens CO_2_ binding, and shifts the global minimum toward chemisorption at more negative potentials, highlighting the critical role of symmetry breaking in CO_2_ activation.

To quantify the potential-dependent crossover in CO_2_ adsorption modes, we define the “transorption” potential (*U*_trans_) as the applied potential at which the thermodynamic minimum switches from physisorption to chemisorption (*C*_2*v*_–*C*_2*v*_). For Ni–N_4_, the physisorption minimum is essentially potential-invariant in Landau free energy (ΔΩ), net charge transfer (≈0), and O–C–O angle (near-linear), whereas the chemisorption minimum shows a strong potential dependence: ΔΩ decreases (stabilizes) with more negative *U*, the magnitude of electron transfer to CO_2_ increases, and the O–C–O angle progressively decreases (greater bending), crossing the physisorption minimum at *U*_trans_ = −1.95 V (Figure 4a). The electron transfer and bond angle characteristics distinguish the two modes and verify the transition. Consistently, the projected density of states (PDOS) analysis reveals substantial electronic restructuring at the Ni center upon chemisorption compared to the pristine surface and physisorption state (Figure 4b).

**Figure 4:**
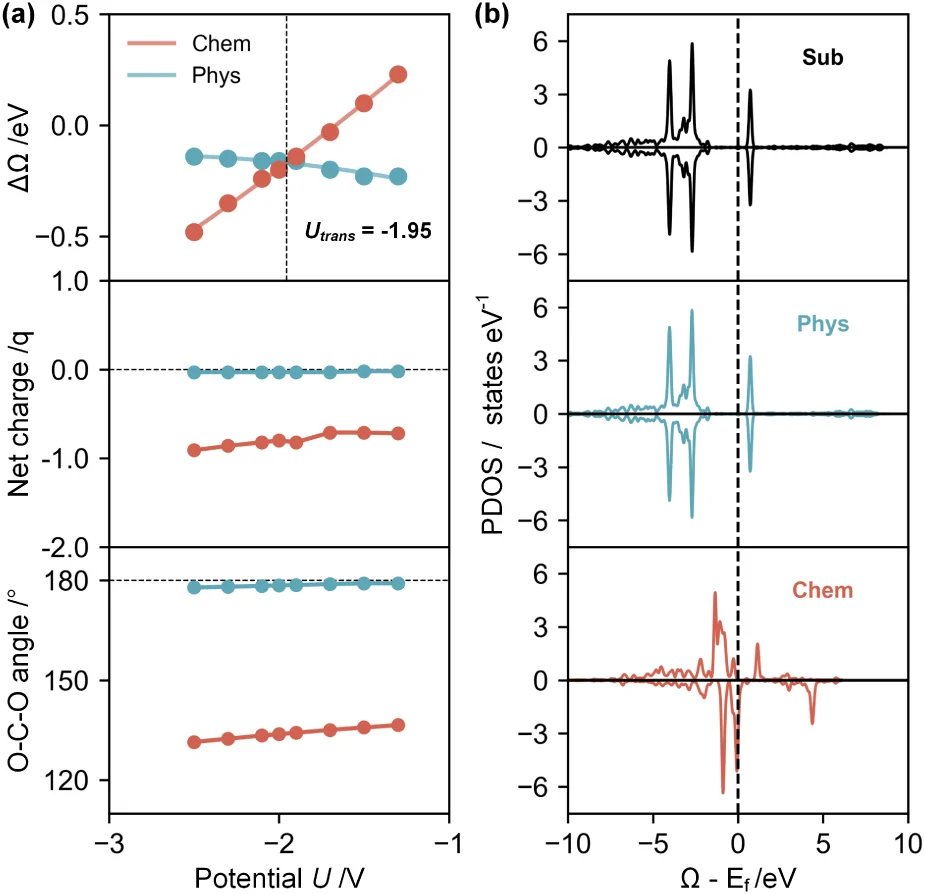
(a) Potential-dependent Landau adsorption free energies (ΔΩ), net charge transfer (*q*), and O–C–O bond angle for the minimum-energy states on the physisorption (blue) and chemisorption (red) curves of Ni–N_4_. The vertical dashed line marks the transorption potential *U*_trans_ = −1.95 V. (b) Partial density of states (PDOS) of the Ni active center for the pristine substrate (Sub), physisorbed (Phys), and chemisorbed (Chem) states at *U*_trans_, referenced to the Fermi level (*E*_f_). Corresponding results for the other nine TMs are given in Figures S14–S22.

With the Ni–N_4_ case established, we then explored whether this crossover holds true across the 3d TM–N_4_ SACs. Surveying all 3d TM–N_4_ SACs (Figures S14–S22) reveals analogous profiles, in which the physisorption minimum is weakly potential dependent, whereas the chemisorption minimum stabilizes with increasing overpotential, yielding a well-defined *U*_trans_ for each metal (the corresponding values are summarized in Table S9).

To assess whether the concept of *U*_trans_ can be extended beyond TM–N_4_ SACs, we applied the same constant-potential framework to the dropping Hg electrode. Notably, applying the same calculation framework to a Hg slab yields a crossover potential of *U*_trans_ = −1.89 V (Figure S23a–b and Table S10), consistent with the experimental value inferred from polarographic measurements (see Part 1 of the Supporting Information), where CO_2_^•–^ formation is observed at −1.98 V vs SHE.^76–78^ At this potential, CO_2_ adopts a bent geometry in the chemisorbed state (Figure S23c), consistent with prior reviews of adsorbed CO_2_.^79,80^ The bent geometry closely resembles that of the solvated CO_2_^•–^,^78,81^ as also reproduced in our calculations (Figure S23d).

Therefore, the CO_2_ adsorption-mode transition emerges as a recurring, potential-dependent feature across the systems examined here; for TM–N_4_ SACs, it is governed by symmetry lowering and electronic reorganization at sufficiently negative potentials. At the same time, any extension of this concept to broader catalyst families or other electrocatalytic reactions should be regarded as a promising future direction rather than an established conclusion of the present work.

### Machine-Learning Analysis

ML provides a data-driven approach to uncovering complex structure–property relationships and identifying key descriptors that may be overlooked in conventional analyses.^82–85^ To probe the factors governing the CO_2_ adsorption-mode crossover across 3d TM–N_4_ SACs, we employed the SISSO method^74^ to isolate the key parameters influencing *U*_trans_. We emphasize that SISSO is used here not as a predictive model for unexplored catalysts, but as a data-driven tool to validate and rationalize the orbital-level mechanisms governing CO_2_ chemisorption identified in the previous section. To this end, we selected 21 features (Table 1): 8 atomic descriptors of the transition metals (Atomic (TMs)), 2 bulk metal properties (Bulk (TMs)), and 11 surface descriptors of the SACs (Surface (SACs)). The atomic and bulk features were obtained from the consistently generated data set of Han et al.,^86^ whereas the surface descriptors were calculated in this work. These features can be further categorized as 17 electronic structures, 2 structural stabilities, and 2 general physical properties.

**Table 1:** Features Used for Machine Learning

	Feature	Symbol (formula)	Label (plot)
Atomic (TMs)	Electronegativity^a^	*EN*	*F*_1_
	First ionization energy (eV)^a^	*E* *1_a_*	*F*_2_
	Binding energy of metal dimers (eV)^b^	*E* *b_a_*	*F*_3_
	Electron numbers in s and d orbitals^a^	*N*_*s+d*_	*F*_4_
	s and d orbital radii (Å)^a^	*R*_*s*_, *R*_*d*_	*F*_5_, *F*_6_
	Atomic radius (Å)^c^	*R*_*a*_	*F*_7_
	Mass (g/mol)^c^	*M*	*F*_8_
Bulk (TMs)	Cohesive energy (eV)^b^	*E_coh_b__*	*F*_9_
	Energies of *d*-band center (eV)^a^	*E_d_b__*	*F*_10_
Surface (SACs)	Energies of $d_{x^{2}-y^{2-}}$ , $d_{z^{2-}}$ , *d_xy_*-, *d_xz_*- and *d_yz_*-band center (eV)^a^	${E_{x^{2}-y^{2}}}_{s}$ , ${E_{z^{2}}}_{s}$ , ${E_{xy}}_{s}$ , ${E_{xz}}_{s}$ , ${E_{yz}}_{s}$	*F*_11_, *F*_12_, *F*_13_, *F*_14_, *F*_15_
	Fermi level (eV)^a^	${E_{F}}_{s}$	*F*_16_
	Electron numbers in different orbitals^a^	${N_{x^{2}-y^{2}}}_{s}$ , ${N_{z^{2}}}_{s}$ , ${N_{xy}}_{s}$ , ${N_{xz}}_{s}$ , ${N_{yz}}_{s}$	*F*_17_, *F*_18_, *F*_19_, *F*_20_, *F*_21_

a Electronic structures.

b Structure stabilities.

c Physical properties.

To assess whether *U*_trans_ can be captured by a single variable, we first examined its linear correlations with each feature. The resulting R^2^ values (Figure 5a) are uniformly low (0.004–0.39), and the pairwise plots (Figures S24–S48) show weak or highly dispersed trends. Thus, no single descriptor suffices to explain *U*_trans_, and a multifeatured model is required to capture the adsorption-mode crossover. Having established the need for a multifeatured model, we next assessed interdependencies among descriptors. We employed the Pearson correlation matrices (Figure 5b) to visualize pairwise relationships among the features. As expected, when treating individual *d* orbitals as separate descriptors, strong multicollinearity is observed among the orbital-resolved *d*-band centers ( ${E_{x^{2}-y^{2}}}_{s}$ , ${E_{z^{2}}}_{s}$ , ${E_{xy}}_{s}$ , ${E_{xz}}_{s}$ , ${E_{yz}}_{s}$ ) and corresponding occupancies ( ${N_{x^{2}-y^{2}}}_{s}$ , ${N_{z^{2}}}_{s}$ ${N_{xy}}_{s}$ , ${N_{xz}}_{s}$ , ${N_{yz}}_{s}$ ). This correlation reflects their collective participation in TM–CO_2_ bonding. Although correlated, these orbitals contribute through distinct symmetry channels and therefore retain independent mechanistic significance, enabling the differentiation between σ-donation and π-back-donation contributions. Notably, several of these features also emerged as critical in our symmetry/Walsh analysis of adsorption-mode transition. Therefore, keeping these correlated features enhances the interpretability of our ML model and preserves sensitivity to the distinct bonding channels that govern CO_2_ chemisorption.

**Figure 5:**
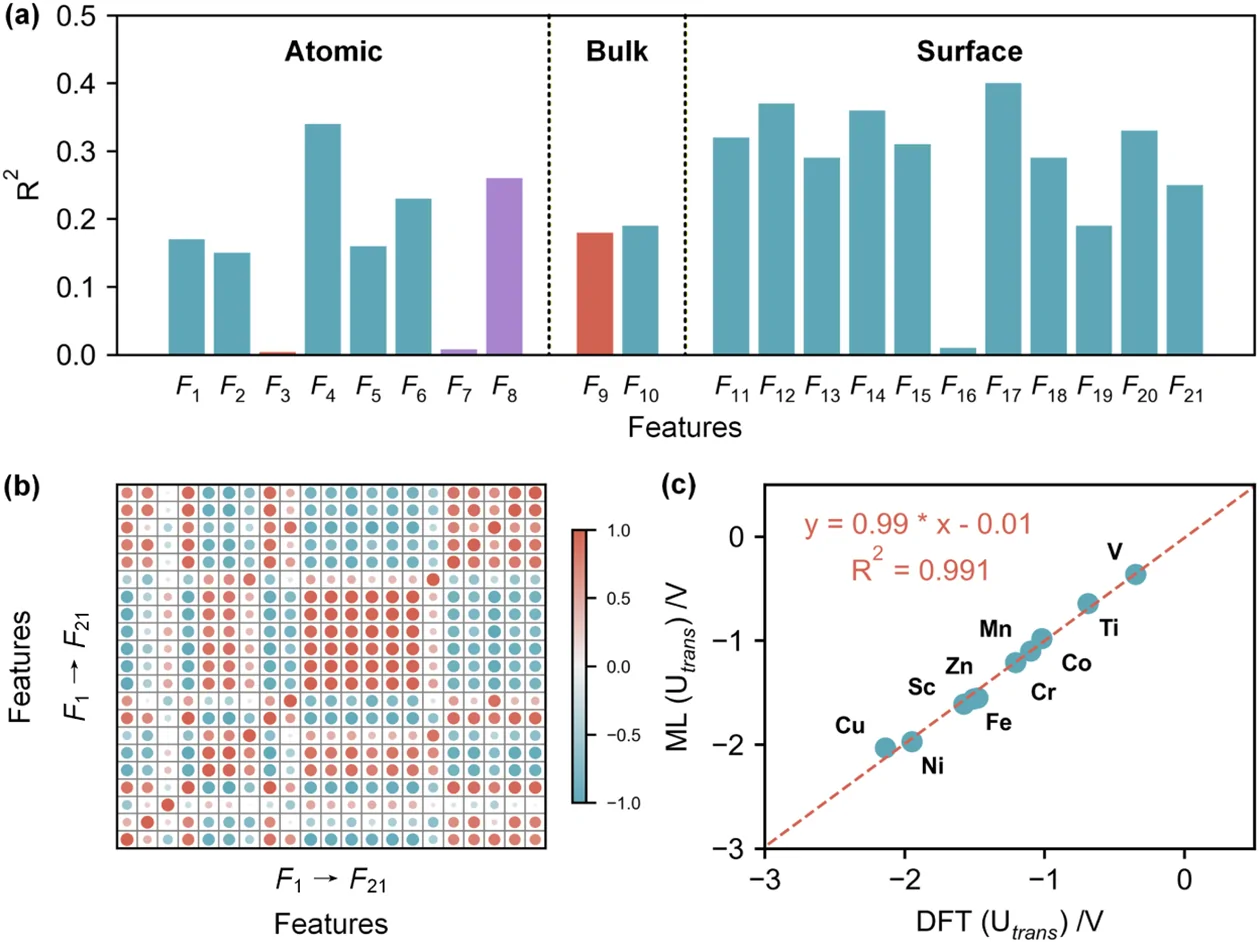
(a) Summary of R^2^ values corresponding to the correlations between *U*_trans_ and each feature. The features of electronic structures, structure stabilities, and physical properties are colored in blue, red, and purple, respectively. (b) Pearson correlations between the selected 21 features. (c) The *U*_trans_ comparison between the results from the ML simulations and DFT calculations.

To construct a compact and physically interpretable model for *U*_trans_, we examined SISSO models with increasing dimensionality. The resulting 1D SISSO model is given by

6 $$U_{\mathrm{trans}}=-0.48\times \ln\left(\left|{E_{d}}_{b}-{E_{x^{2}-y^{2}}}_{s}\right|-\left|{E_{yz}}_{s}-{E_{z^{2}}}_{s}\right|\right)-1.58$$

which combines three surface electronic features and one bulk descriptor. The 2D SISSO model is summarized in Table S11. The 1D model correlates strongly with the DFT-computed *U*_trans_, achieving an R^2^ value of 0.991 (Figure 5c), whereas the 2D model improves the fit only marginally (R^2^ = 0.992) at the cost of substantially greater complexity.

To evaluate the robustness and possible overfitting, we performed LOOCV for both 1D and 2D SISSO models. The resulting expressions for each fold are listed in Tables S12–S13, and the corresponding held-out predictions are summarized in Table S14. For the 1D model, 9 out of 10 folds recover the same four primary descriptors identified in the full-data fit, while the remaining fold yields a closely related expression that still retains two of those four descriptors. This strong recurrence indicates that the 1D descriptors are robust and not controlled by any single data point. By contrast, the 2D SISSO model exhibits notable fold-to-fold variability in the second component, reflecting increased sensitivity to data perturbation and hence lower robustness. The held-out predictions further demonstrate a clear difference in cross-validated performance: the 1D model yields LOOCV MAE, RMSE, and R^2^ values of 0.090 V, 0.136 V, and 0.95, respectively, whereas the corresponding errors for the 2D model deteriorate substantially to 0.520 V, 0.828 V, and 0.23. Thus, the 2D model shows poorer held-out predictive performance and lower descriptor stability. Collectively, these results support the 1D SISSO model as the more robust and interpretable framework adopted in this work.

To quantify the relative importance of each component in the 1D SISSO model, we performed an ablation study, in which each primary descriptor was individually replaced by its data set-averaged value, and the resulting increase in prediction mean square error was used to estimate its contribution. The relative contributions are 31.75%, 26.96%, 24.51%, and 16.78% for ${E_{x^{2}-y^{2}}}_{s}$ , ${E_{yz}}_{s}$ , ${E_{z^{2}}}_{s}$ , and *E_d_b__*, respectively. Thus, the surface electronic descriptors dominate the model performance, together accounting for more than 80% of the total contribution.

With the 1D model established, we next examine the physical origin of the main descriptors in the CO_2_ adsorption process. During chemisorption, the CO_2_ molecule transforms from linear D_∞*h*_ to bent C_2*v*_, reorganizing its frontier molecular orbitals (Figure 2b). The originally degenerate π_u_ and π_g_ sets split into (*b*_1_ and *a*_1_) and (*b*_2_ and *a*_2_), respectively. Based on symmetry considerations, four symmetry-allowed metal–ligand couplings are found: (1) CO_2_-b_1_ with Ni-b_1_ in *C*_2*v*_ (*d_xz_*) and with Ni-b_2g_ in *D*_2*h*_, (2) CO_2_-a_1_ with Ni-a_1_ in *C*_2*v*_ $(d_{z^{2}}$ or $d_{x^{2}-y^{2}})$ and with Ni-a_g_ in *D*_2*h*_, (3) CO_2_-a_2_ with Ni-a_2_ in *C*_2*v*_ (*d_xy_*) and with Ni-b_1g_ in *D*_2*h*_, and (4) CO_2_-b_2_ with Ni-b_2_ in *C*_2*v*_ (*d_yz_*) and with Ni-b_3g_ in *D*_2*h*_. Consistent with the ML analysis, the Ni *d_yz_*, $d_{z^{2}}$ , and $d_{x^{2}-y^{2}}$ orbitals contribute most significantly to the CO_2_ chemisorption, because they engage most strongly with the bent CO_2_-*a*_1_ (new LUMO) and *b*_2_ (new HOMO) states. Thus, CO_2_ binding is governed by HOMO → metal donation (CO_2_-b_2_, lone electron pair to Ni *d_yz_*) and metal → LUMO back-donation (Ni $d_{z^{2}}$ or $d_{x^{2}-y^{2}}$ into CO_2_-a_1_), in line with the observed charge-transfer trends and orbital-energy alignment.

Interestingly, although the bent-CO_2_-a_1_ derives from the linear π_u_ set, its interaction with Ni- $d_{z^{2}}$ is predominantly σ-bonding, with no nodal plane along the Ni–C axis. This interaction presents an unusual σ back-donation, in which electron density flows from Ni $d_{z^{2}}$ into the CO_2_-a_1_ acceptor. The charge transfer map (Figure 3b) and Walsh diagram (Figures S5–S7) both corroborate electron injection into the *a*_1_ orbital upon chemisorption. This σ back-donation motif echoes bonding patterns in TM-ligand complexes,^87–90^ but here is enabled by symmetry lowering of the Ni–N_4_ site from D_2*h*_ to C_2*v*_, which shortens the Ni–C distance and enhances the overlap between CO_2_-a_1_ and Ni- $d_{z^{2}}$ orbitals. The resulting stronger coupling increases orbital splitting and charge transfer, ultimately promoting CO_2_ activation and stabilizing the chemisorbed state. Therefore, the consistency among the electronic structure analysis, the symmetry/Walsh picture, and the SISSO model underscores the critical role of TM *d*-orbitals in stabilizing CO_2_ chemisorption.

### CO_2_RR Processes Based on FCP Computations

In this section, we analyze CO_2_-reduction processes on Ni–N_4_ SAC. The Landau free energy diagrams at selected potentials are shown in Figure 6a, with chemisorption and physisorption states distinguished by colored and black lines, respectively, and the complete reaction pathway is illustrated in Figure S49. Because each free-energy profile in Figure 6a was obtained self-consistently at its own applied potential, the differences between the profiles do not arise from a simple rigid shift by the potential difference; rather, they reflect the potential-dependent electron transfer with each intermediate and scale approximately with −Δ*N_e_* Δ*U* when two potentials are compared.

**Figure 6:**
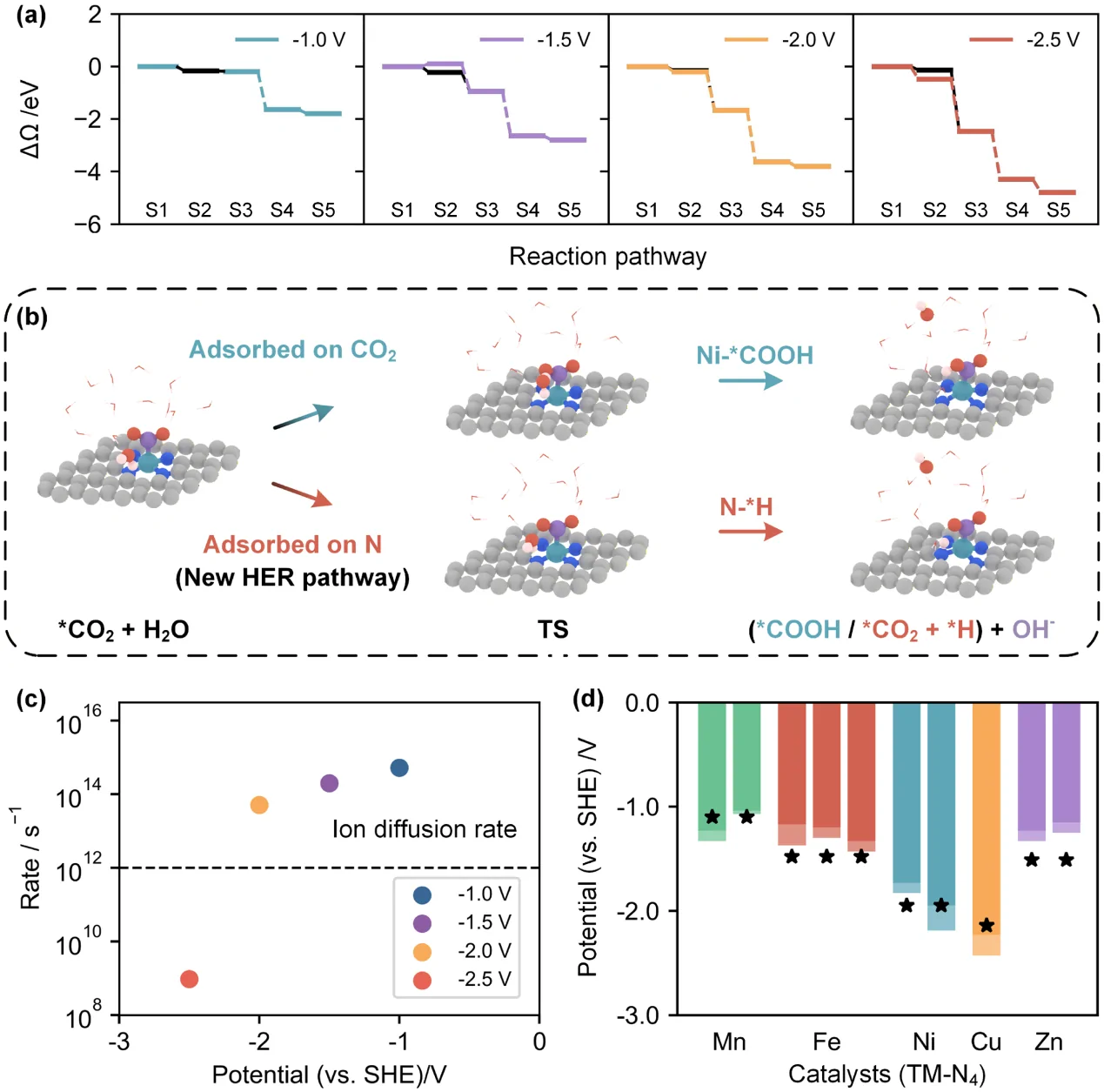
(a) Landau free energy diagram for CO_2_RR on Ni–N_4_ SAC under applied potentials of *U* = −1.0, −1.5, −2.0, and −2.5 V vs SHE. The reaction states are denoted as S1–S5, corresponding to S1 = *+CO_2_, S2 = *CO_2_, S3 = *COOH, S4 = *CO, and S5 = * + CO. The physisorption branch is shown in black, whereas the CO_2_ chemisorption branch is shown in the color corresponding to each applied potential; at *U* = −1.0 V, only physisorption is accessible. (b) Competing hydrogenation pathways on Ni–N_4_ SAC following CO_2_ chemisorption: *COOH formation versus a coordinating-N-mediated HER pathway. The aqueous environment is represented by stick models, while the reaction intermediates are highlighted using ball-and-stick models. (c) Calculated electron-hopping rates at different applied potentials; the dashed line denotes the estimated ion-diffusion rate. (d) Calculated *U*_trans_ values (asterisks) versus experimental onset potential for CO yield decline (bars) across various TM–N_4_ SACs (M = Mn, Fe, Ni, Cu, and Zn). The lighter bars denote the experimental potential intervals after conversion to the SHE scale. This catalyst-by-catalyst representation complements the parity-style comparison shown in the Figure 1 inset, and the dedicated parity plot is shown in Figure S56.

Across potentials, the free-energy profile is generally exergonic, except for the route involving CO_2_ chemisorption at *U* = −1.5 V. Notably, at *U* = −1.0 V, only physisorption occurs, as chemisorption is not observed at this less negative potential. At *U* = −1.5 V, CO_2_ chemisorption becomes reachable but remains less stable than physisorption. At *U* = −2.0 V, close to the computed *U*_trans_ = −1.95 V, chemisorption slightly surpasses physisorption (Figure 4a), and at *U* = −2.5 V, it becomes clearly dominant. Therefore, CO_2_RR proceeds primarily via a physisorption-dominated pathway (with CEPT mechanism) at less negative potentials, and transitions to a chemisorption-dominated pathway (with DEPT mechanism) once the applied potential is more negative than *U*_trans_.

To quantify how applied potential reshapes the CO_2_RR mechanism, we tracked the transition of the RLS, the elementary step with the largest free-energy span ΔΩ per the Bell–Evans–Polanyi principle.^91,92^ As shown in Figure 6a, at −1.0 V, the RLS is *COOH formation on the physisorption pathway (black segment at the second step; chemisorption is absent at this potential), whereas upon driving the potential more negative, the CO release free-energy span surpasses that of *COOH formation, resulting in the RLS shift between −1.0 and −1.5 V. Beyond this transition, at more negative potentials of −2.0 to −2.5 V, the energy differences for most steps decrease; however, the CO release span consistently maintains the largest and thus the RLS. Notably, the CO release free-energy change is nearly constant at *U* = −1.0, −1.5, and −2.0 V, then drops at −2.5 V, foreshadowing CO production change at the most reducing potentials.

We next examine how these RLS transitions relate to the CO yield behavior depicted in Figure 1. At potentials less negative than −1.0 V, prior CP studies showed that the *COOH step is endergonic but progressively stabilizes as the potential becomes more negative,^38,43^ consistent with its role as the RLS and with the experimentally observed initial rise in CO yield.^93–95^ At −1.0 V, the RLS is *COOH formation on the physisorption pathway (black segment at the second step; chemisorption is absent at this potential). As the potential becomes more negative, the free-energy span for CO release surpasses that of *COOH formation, resulting in an RLS shift between −1.0 and −1.5 V. Once CO release becomes rate-limiting, its free-energy span remains nearly unchanged up to −2.0 V. Within the Bell–Evans–Polanyi framework, this corresponds to a nearly constant kinetic bottleneck, which naturally gives rise to the experimentally observed plateau in CO yield.^23,31^ Beyond −2.0 V, the production plateau terminates as the CO-release free energy span changes and additional factors, including HER onset at N sites and slower electron-transfer, begin to depress activity, leading to the eventual decline in CO yield. Taken together, these mechanistic elements reproduce the CO yield trend observed experimentally, including the initial rise, the intermediate plateau, and the eventual decline (Figures 1 and S1).

To rationalize the experimentally observed postplateau decline in CO yield, we first note that its onset potential closely coincides with *U*_trans_ (Figure S1), where CO_2_ adsorption on Ni–N_4_ SAC shifts from physisorption to chemisorption. This correspondence suggests a mechanistic link, inspiring us to propose a new HER pathway at more negative potentials: the coordinated N atoms may become active for HER once CO_2_ chemisorbs, introducing a competing pathway that consumes protons and electrons (Figure 6b) and thereby suppresses CO production. To probe this possibility, we computed the reaction profiles for the first branching hydrogenation after CO_2_ adsorption, specifically, *COOH formation and competing *H adsorption on one of the four coordinating N sites, namely, Ni–N_4_ SAC, at *U*_trans_ using the CI-NEB method (Figure S50), and incorporated the resulting activation barriers and energetics into microkinetic selectivity analyses (Figure S51). At *U*_trans_, the *COOH formation and competing N-site H adsorption have comparable activation barriers of 0.17 and 0.20 eV, respectively; because *COOH formation remains thermodynamically more favorable, the N-site HER pathway is kinetically accessible but not dominant under these conditions. These representative steps were selected because the first hydrogenation step determines whether the reaction branch proceeds toward CO_2_RR or HER at *U*_trans_. The preliminary trends agree well with experiment, supporting the plausibility of the N-site HER pathway. Nevertheless, these microkinetic results should be viewed as preliminary mechanistic insights rather than a complete treatment of the reaction environment. A more comprehensive constant-potential DFT study, ideally combined with targeted experiments, will be needed to further validate this hypothesis. In addition, coupling microkinetic simulations with a continuum transport model will be essential for rigorously assessing hydrodynamic diffusion and other transport effects.

Notably, this unique potential-dependent, CO_2_-chemisorption-triggered HER mechanism proposed here differs fundamentally from conventional N-doping strategies for HER, where N primarily modulates the electronic structure of adjacent metal^96–98^ or carbon^99^ sites without serving as the direct H-adsorption center. In contrast, our results indicate that the coordinating N atom itself is an active site for HER once CO_2_ is chemisorbed at the Ni–N_4_ site, diverting protons/electrons and suppressing CO formation. In addition, Boppella et al. recently demonstrated experimentally that N atoms proximal to the catalytic TM center can participate in H adsorption,^100^ providing direct evidence that nearby N motifs can serve as proton-binding sites. This observation supports the mechanistic relevance of our prediction, suggesting that the N atoms surrounding Ni–N_4_ may indeed act as additional HER-active sites under CO_2_RR conditions

Beyond thermodynamics, we examined electron transfer (ET) as a second factor that can affect CO yield, particularly at strongly negative potentials. We evaluated the electron hopping rate between the adsorbate and Ni–N_4_ using the method proposed by Nørskov, Strasser, Chan, and coworkers,^16,101^ which was originally formulated for chemisorbed species with an adsorbate PDOS peak at the Fermi level. Here, we generalize this approach to systems where the adsorbate PDOS peak is offset from *E*_f_ or the adsorbate has a finite band gap, thereby enabling its application to adsorbates with metallic or semiconducting behavior (derivation provided in Part 2 of the Supporting Information). Since CO_2_ prefers physisorption at less negative potentials, we selected *COOH, a chemisorbed intermediate, as the probe species. The half-width (Δ) was obtained from the *COOH PDOS near *E*_f_ (Figures S52–S55), and the corresponding DOS height was normalized to the maximum DOS at the same energy across comparison cases. The ET rate was then estimated using Fermi’s Golden Rule (for details, please refer to Part 2 of the Supporting Information).

To assess whether ET limits activity at high overpotentials, we quantified ET rates versus applied potentials. Figure 6c summarizes ET rates, in which the dashed line represents the estimated ion diffusion rates proposed by Nørskov’s group on the Pt(111) facet and Fe–N_4_ SAC.^16,102^ From −1.0 to −2.0 V, ET rates exceed the ion diffusion rate by roughly 2 orders of magnitude, indicating that ET is not rate-limiting at modest overpotentials. In contrast, between −2.0 and −2.5 V, the ET rate drops sharply and falls below the ion diffusion benchmark, suggesting that ET may contribute to the decline in CO production. This observation aligns with experimental findings, where the CO yield significantly decreases at more negative potentials following a production plateau.^23,31^ Thus, while ET does not hinder CO_2_ reduction performance at mild potentials, it can contribute to the observed decline in CO yield under strongly reducing conditions.

Building on the finding that the *U*_trans_ coincides with the experimentally observed onset of CO yield decline on Ni–N_4_ SAC, we further examined whether this correlation between computations and experiments holds across 3d series. Figure 6d compares five TM–N_4_ SACs (TM = Mn, Fe, Ni, Cu, and Zn): experimental decline potentials are shown as bars,^23,31,103–110^ while computational *U*_trans_ data appear as asterisks. To facilitate direct comparison between experiment and theory across catalysts, we present the computed *U*_trans_ values and the experimentally observed onset potentials for CO-yield decline side by side in Figure 6d. This catalyst-by-catalyst representation makes the agreement in both absolute range and relative ordering more transparent. For a complementary global view, the corresponding parity-style comparison is also shown in the inset of Figure 1, and the dedicated parity plot is shown in Figure S56. The experimental data sets for each element are detailed in Figures S1 (Ni) and S57 (Mn, Fe, Cu, Zn). To reflect the discrete potential steps used in the experiments, we employ a lighter color range in the diagram to indicate the measurement step size and the associated uncertainty. The exact optimal potential in experiments remains unknown, but is expected to fall within these intervals. It should be noted that, although the experimental catalysts are generally assigned to TM–N_4_ motifs, their local coordination environments may deviate from the idealized TM–N_4_ structures considered in our calculations. Moreover, the experimental data were collected from catalysts prepared and evaluated under different conditions. Recent round-robin testing has shown that appreciable variability in catalytic performance can persist even when identical catalyst batches and standardized testing protocols are used across laboratories, highlighting the uncertainty associated with cross-study comparisons of literature-derived experimental data.^111^ Accordingly, the comparisons in Figures 6d and S56 are intended as a semiquantitative cross-catalyst assessment rather than a strict one-to-one comparison among structurally identical TM–N_4_ sites.

Within these limitations, the computed *U*_trans_ trend, Mn < Zn < Fe < Ni < Cu, well matches the experimental findings, which highlights the critical role of CO_2_ chemisorption and supports *U*_trans_ as a reliable parameter for predicting CO production trends across TM–N_4_ catalysts. We note that previous studies have explored geometry-based descriptors for CO_2_ activation and catalytic performance, focusing on structural features such as bond elongation and bending on metal^112^ and metal oxide surfaces.^113^ In contrast, our work introduces *U*_trans_ as a potential-based descriptor, providing an alternative approach with experimentally measurable quantities.

In addition to the correlation between *U*_trans_ and CO yield decline, the free energy diagram revealed another agreement with experiments. Specifically, only Fe– and Cu–N_4_ SACs exhibit an endergonic CO release process (Figures S58 and S59), whereas the other three TM–N_4_ SACs (Mn, Ni, and Zn) show exergonic CO release (Figures S60 and S61). Since CO is a key precursor for C_2_ product formation, strong CO binding on Fe– and Cu–N_4_ SACs may inhibit CO desorption, favoring CO poisoning while simultaneously promoting the formation of C_2_ products. Notably, our results align well with the experimentally observed CO poisoning on Fe–N_4_ SAC^114^ and the presence of C_2_ products on Cu–N_4_ catalysts.^108,115^ In contrast, CO remains the primary product for Mn–,^103,116^ Ni–,^27,30^ and Zn–N_4_ SACs,^109,117,118^ consistent with their weaker CO binding and facile desorption.

To evaluate the thermal stability of key intermediates, we performed fixed-electron-number AIMD simulations across the five TM–N_4_ SACs using electron numbers initialized from the corresponding CP-DFT calculations at *U*_trans_. The energy evolution profiles and C–TM distances of the adsorbed intermediates are presented in Figures S62–S71. Despite thermal fluctuations in orientations and C–TM separation during the simulations, chemisorbed *CO_2_ remained stably bound on all systems, confirming the viability of the chemisorption mode at *U*_trans_ and supporting a mechanistic shift from CEPT to DEPT. In contrast, CO exhibits metal-dependent behavior: spontaneous desorption events appear on Mn–, Ni–, and Zn–N_4_ SACs, whereas CO remains persistently adsorbed on Cu– and Fe–N_4_ SACs. These findings are consistent with our previous thermodynamic analysis (endergonic CO release on Fe/Cu) and align well with experimental observations, where Fe–N_4_ SAC tends to suffer from CO poisoning, while Cu–N_4_ SAC presents good performance for C_2_ product formation.

To delineate the scope, we restricted cross-metal comparisons to five representative TM–N_4_ SACs. Although Co–N_4_ has been studied experimentally, we did not include it here because of its CO_2_RR performance is markedly inferior to Ni–N_4_.^21,22,119^ Moreover, while Co-based molecular complexes have shown promising CO_2_ reduction activity,^120^ their architectures differ fundamentally from graphene-anchored SACs: they lack a robust carbon matrix as a secondary coordination shell, and their coordinating N atoms are embedded within the 5-membered carbon rings rather than a graphene lattice. Consequently, performance data from Co-containing molecular systems are not directly transferable to the TM–N_4_ models investigated in this work.

Recent constant-potential studies have highlighted that the electronic configuration of the metal center can evolve under bias. In particular, Cui et al. examined CO_2_RR–HER competition on Ni–N_4_ under an OH-ligated Ni assumption, mapping Faradaic-efficiency (FE) changes over −0.4 to −0.9 V (vs RHE).^46^ Their study highlights ligand effects at the Ni center and a potential-induced hybridization-state transition of Ni–N_4_ (from an inert dsp^2^ to an active d^2^sp^3^ configuration), and stands among the earliest CP works to directly simulate FE diagrams to rationalize CO–H_2_ selectivity. Our work is complementary and adsorbate-centric: with the CP framework, we uncover a potential-driven transition of CO_2_ adsorption modes that sets the onset of Ni-site occupation by chemisorbed CO_2_ and predicts the decline of CO partial current density beyond *U*_trans_. This crossover naturally activates neighboring N atoms as HER sites, providing a mechanistic route for CO_2_RR-HER competition at high overpotentials. Thus, while Cui et al. rationalize selectivity via Ni-center hybridization and ligand effects, we explain the full PCD evolution (rise → plateau → decline) via adsorption-state switching and its kinetic consequences. Together, these two views offer orthogonal, mutually reinforcing levers for understanding and optimizing TM–N_4_ SACs under working conditions.

Recent experimental studies have expanded the description of SACs beyond the local TM–N_4_ motif, highlighting the importance of both extended electronic interactions and potential-induced structural evolution. Wang et al. showed that long-range π-electron delocalization beyond the second coordination shell can substantially influence the electronic structure of the Ni center and stabilize the key *COOH intermediate.^121^ Shen et al. further demonstrated that an initially defined CuN_4_ site can undergo electrochemically induced coordination reconstruction to a self-healed CuN_1_O_2_ configuration under working conditions.^122^ These findings emphasize that experimentally relevant SAC active sites may involve electronic and structural degrees of freedom that are not captured by an idealized local TM–N_4_ model. Accordingly, because the present work focuses on idealized graphene-based TM–N_4_ structures, the calculated *U*_trans_ values are not expected to be directly and quantitatively transferable to such more complex environments. Nevertheless, the physical concept underlying *U*_trans_ could be tested in these systems by explicitly incorporating their extended electronic environments and operando coordination structures into constant-potential calculations. Such extensions may provide a useful route toward connecting potential-dependent adsorption-state transitions with realistic catalyst structures and experimentally observed catalytic behavior.

## Conclusion

In summary, we employed the fully converged constant-potential method to investigate the potential-dependent transition of CO_2_ adsorption mode across ten 3d TM–N_4_ SACs, with detailed analysis for Ni–N_4_ and extensions to Mn, Fe, Ni, Cu, and Zn where experimental benchmarks exist. Landau free energy profiles exhibit three CO_2_ adsorption modes, including one physisorption (*D*_∞*h*_–*D*_2*h*_) and two chemisorption (*C*_2*v*_–*D*_2*h*_ and *C*_2*v*_–*C*_2*v*_) states. Walsh diagrams and ML analyses indicate that increasingly negative potential stabilizes CO_2_ chemisorption by enhancing the σ-type back-donation and shortening the C–TM distances via symmetry lowering at the TM–N_4_ center.

We further introduced the concept of the “transorption” potential (*U*_trans_), defined as the threshold potential at which chemisorption becomes thermodynamically preferred over physisorption, and used it to map CO yield evolution on Ni–N_4_ SAC across three regimes: (1) At low overpotentials, CO_2_ is physisorbed and *COOH formation is the RLS and its progressive stabilization with increasing overpotential accounts for the initial rise in CO yield; (2) At moderate overpotentials, CO_2_ still prefers physisorption thermodynamically, but the RLS shifts to CO desorption with a nearly potential-invariant span, giving a CO yield plateau; (3) Beyond *U*_trans_, CO_2_ prefers chemisorption, which activates N-site for HER and reduces electron transfer rates, jointly suppressing the CO yield.

By integrating constant-potential thermodynamics, orbital-symmetry reasoning, machine learning, and microkinetics, this work establishes *U*_trans_ as a predictive, DFT-derived descriptor that bridges computational and experimental investigation of TM–N_4_ SACs. Critically, the framework quantitatively rationalizes the experimentally measured CO yield trend, capturing the rise-plateau-decline behavior and pinpointing the onset of the drop at *U*_trans_. Practically, *U*_trans_ provides a screenable operating handle to maximize CO production while anticipating selectivity losses. Conceptually, the σ-back-donation/symmetry-lowering paradigm offers general design principles for activating linear molecules on atomically dispersed sites. Looking forward, integrating *U*_trans_ into ML/AI pipelines may offer a promising route for high-throughput, bias-aware screening of SACs and may inspire future exploration of related electrocatalytic transformations beyond CO_2_RR.

## Supplementary Material





## Notes

Experimental data set of partial current density as a function of applied potential on Ni–N_4_ SAC; structure model of TM–N_4_ SACs; assessment of the fixed-electron-number AIMD treatment; four possible CO_2_ chemisorption configurations; CO_2_ Walsh diagram at *U* = −1.5, −2.0, and −2.5 V vs SHE on Ni–N_4_ SAC; Walsh diagram with corresponding PDOS of CO_2_ along the chemisorption and physisorption curves at *U* = −1.5, −2.0, and −2.5 V vs SHE on Ni–N_4_ SAC; adsorption energy diagram, net charge transfer, and CO_2_ angle variation for the minimum position on the physisorption and chemisorption curves on TM–N_4_ SAC as a function of applied potential *U*, as well as PDOS of TM active center before and after the CO_2_ physisorption/chemisorption at *U*_trans_; adsorption energy diagram and CO_2_ angle variation of the CO_2_ physisorption and chemisorption on Hg-(100) facet, as well as the CO_2_ chemisorption configuration on Hg-(100) facet and CO_2_ radical anion configuration in solvation environment; linear relationships between the *U*_trans_ of CO_2_ and 21 features; illustration of the CO_2_RR process on Ni–N_4_ SAC; illustration of CO_2_RR through CO_2_ chemisorption and the new HER mechanism pathway; CI-NEB diagram of *COOH formation and competing *H adsorption; comparison of Faradaic efficiencies between computation and experiment; PDOS projected onto COOH s- and p-states on Ni–N_4_ SAC at −1.0, −1.5, −2.0, and −2.5 V; free energy diagram of TM–N_4_ SACs at *U*_trans_; parity plot comparing the calculated *U*_trans_ value with the experimental onset potential for CO yield decline; energy evolution and C–Ni distance variation on TM–N_4_ SACs at *U*_trans_ under 300 K; values of *U* parameters for DFT calculations; spin examination for CO_2_ adsorption on Ni–N_4_ SAC; OMC-constrained energies of Ni–N_4_ SAC with CO_2_ chemisorption and physisorption; *U*_trans_ value of 3d TM–N_4_ SACs; surface energy of seven low-index Hg facets; 2D SISSO model for full-set data; 1D SISSO model from LOOCV; 2D SISSO model from LOOCV; held-out prediction for each TM–N_4_ system from LOOCV examinations (PDF)
Optimized structures (POSCAR) and trajectory files (XDATCAR) from fixed-electron-number AIMD simulations initialized from the corresponding CP-DFT states at *U*_trans_ (ZIP)

## ¶

Department of Chemical and Biomolecular Engineering, University of Tennessee, Knoxville, TN 37996, United States
