# Supplementary material for: Decoding CO2 Adsorption Modes and CO Yield Variation on TM–N4 Single-Atom Catalysts via Constant-Potential Computation and Machine-Learning Investigation
Source: J Am Chem Soc. 2026 Sep 23;148(39):42455–70. doi: 10.1021/jacs.6c13568 (PMC13651630; doi:10.1021/jacs.6c13568)
Supplement: Supplementary file 1 [file ja6c13568_si_001.pdf]

Decoding CO_2 Adsorption Modes and CO Yield Variation on TM-N_4 Single-Atom Catalysts *via* Constant-Potential Computation and Machine-Learning Investigation

Linguo Lu,[1,+] Weibin Chen,[2,+] Jingsong Huang,[3,*] Ian Street,[4] Bobby G. Sumpter,[3] Alexey Serov,[5] Robert L. Sacci,[6] Gabriel M. Veith,[6,‡] William E. Mustain,[4] Ju Li,[7,*] Zhongfang Chen[8,*]

[1] Department of Physics, University of Puerto Rico, Rio Piedras, San Juan, PR 00931, United States

[2] Department of Nuclear Science and Engineering, Massachusetts Institute of Technology, 77 Massachusetts Avenue, Cambridge, MA 02139, United States

[3] Center for Nanophase Materials Sciences, Oak Ridge National Laboratory, Oak Ridge, TN 37831, United States

[4] Department of Chemical Engineering, University of South Carolina, Columbia, SC 29208, United States

[5] Electrification and Energy Infrastructure Division, Oak Ridge National Laboratory, Oak Ridge, TN 37831, United States

[6] Chemical Sciences Division, Oak Ridge National Laboratory, Oak Ridge, TN 37831, United States

[7] Department of Nuclear Science and Engineering and Department of Materials Science and Engineering, Massachusetts Institute of Technology, Cambridge, MA 02139, USA

[8] Department of Chemistry, University of Puerto Rico, Rio Piedras, San Juan, PR 00931, United States

[+] These authors contributed equally to this work.

[‡] Present address: Department of Chemical and Biomolecular Engineering, University of Tennessee, Knoxville, TN 37996, United States

Part 1. Stable Hg surface and potential-dependent CO_2 adsorption

To place our analysis in experimental context, classical studies combining polarography and pulse radiolysis relevant to CO_2 activation are first summarized. Because the one-electron reduction of CO_2 to $CO_2^{\bullet-}$ is irreversible and involves substantial structural reorganization from a linear to a bent geometry,[1-4] its thermodynamic reduction potential cannot be determined exactly from polarographic wave positions.[2] Classical polarographic studies reported an irreversible reduction wave for CO_2 at -2.22 V vs. SCE,[5] which serves as an experimental reference rather than a true thermodynamic value. Complementary pulse-radiolysis experiments generated $CO_2^{\bullet-}$ independently and, when combined with polarographic detection, revealed an oxidation wave for $CO_2^{\bullet-}$ at -1.34 V vs. SCE.[6] On this basis, Koppenol and Rush estimated that the reduction potential of the $CO_2/CO_2^{\bullet-}$ couple lies between -2.22 and -1.34 V vs. SCE, corresponding to -1.98 and -1.10 vs. NHE (≈SHE).[2] These measurements provide an experimental reference for assessing theoretical descriptions of CO_2 activation on mercury (Hg) surface under a strongly reducing potential.

Since CO_2 reduction in polarographic measurements is generally understood to occur on the freshly renewed surface of the dropping Hg electrode, we next modeled CO_2 adsorption on polarized Hg surfaces to evaluate the corresponding reduction potential. Although Hg is liquid at room temperature, herein we employed a crystalline Hg structure from the Materials Project database[7,8] (MP-123, space group R-3m) as a simplified model for the local short-range order in liquid Hg.

We first determined the most stable Hg surface by evaluating the surface energies of seven low-index facets, including (100), (010), (001), (110), (101), (011), and (111). The surface energy (E_{sur}) was evaluated as the sum of the cleavage energy (E_{cleave}) and relaxation energy (E_{relax}). The cleavage energy corresponds to the energy required to cleave a surface slab from the bulk structure, while the relaxation energy represents the energy decrease obtained after structural relaxation of the cleaved slab. The E_{sur}, E_{cleave}, and E_{relax} were therefore calculated as:

$$E_{sur} = E_{cleave} + E_{relax} \quad (s1)$$

$$E_{cleave} = \left(E_{scf} - n\times E_{bulk}\right)/(2S) \quad (s2)$$

$$E_{relax} = \left(E_{opt} - E_{scf}\right)/S \quad (s3)$$

where S is the surface area, n is the number of atoms in the slab, the factor of 2 accounts for the two exposed surfaces, while E_{bulk}, E_{scf}, and E_{opt} denote the energies of bulk Hg per atom, and of the cleaved slab before and after geometry optimizations, respectively.

The calculated surface energies for the seven Hg facets are summarized in **Table S9**. Among the examined surfaces, the Hg-(100) facet exhibits the lowest surface energy, indicating that it is the most stable termination. Accordingly, the Hg-(100) surface was selected for subsequent CO_2 adsorption energy calculations. To simulate the CO_2 activation potential observed in polarographic measurements on the dropping Hg electrode, constant-potential calculations were carried out to evaluate the Landau free energies of CO_2 physisorption and chemisorption on the Hg-(100) surface at various applied potentials.

The calculated results shown in **Figure S22a** indicate that the physisorption energy of CO_2 remains nearly constant across the investigated potential range, whereas the chemisorption energy progressively stabilizes as the electrode potential becomes more negative. This stabilization is accompanied by progressive CO_2 bending (**Figure S22b**), with the molecule adopting a bent geometry in the chemisorbed state (**Figure S22c**), resembling the geometry of the free $CO_2^{\bullet-}$ radical stabilized by solvation (**Figure S22d**). Specifically, the transition from an unfavorable to a favorable chemisorption state occurs at a crossover potential defined as U_{trans}= -1.89 V (vs. SHE), which falls within the experimentally inferred range of -1.98 to -1.10 V for the $CO_2/CO_2^{\bullet-}$ couple.

This agreement supports the interpretation that dropping Hg electrode acts as the catalytic surface for CO_2 activation in polarographic measurements and further establishes U_{trans} as a physically meaningful descriptor.

Part 2. Derivation of electron hopping rate using the Fermi Golden Rule and the Newns-Anderson model

The use of Fermi's golden rule (FGR) combined with the Newns-Anderson (NA) model to estimate electron transfer (hopping) rates in electrocatalysis was first introduced by Nørskov and coworkers and later refined by Strasser, Chan, and coworkers.[9,10] However, those treatments did not provide a full derivation and primarily focused on adsorbates with projected density of states (PDOS) peaks at the Fermi level, and did not consider cases of off-Fermi DOS peaks and gapped (semiconducting) adsorbates. Here, we present a complete and transparent derivation to bridge this gap and generalize the formalism to both conducting and semi-conducting adsorbates.

2.1 Fermi Golden Rule

To quantify the electron hopping rate in electrocatalysis, we begin with the FGR, which provides the transition rate (k) for an electron moving from an initial state $|i\rangle$ to a final state $|j\rangle$. The general expression is:

$$k = \frac{2\pi}{\hbar}\left|\langle j|\hat{H}'|i\rangle\right|^2 \rho(\varepsilon_j) = \frac{2\pi}{\hbar}\left|V_{ij}\right|^2 \rho(\varepsilon_j) \tag{s4}$$

where $\hat{H}'$ is the perturbation Hamiltonian responsible for the transition, V_{ij} is the coupling matrix element between the two states, and $\rho(\varepsilon_j)$ is the density of final states at the energy ε_j. Since FGR is derived from time-dependent perturbation theory, it is applicable in weak-coupling systems, typically associated with physisorption or weak chemisorption.

2.2 Combined with Newns-Anderson Model

To evaluate the coupling matrix element V_{ij}, we then employ the NA model, which describes the interaction between a localized adsorbate orbital and the continuum of metallic states on the catalyst. In this framework, the total Hamiltonian is decomposed into three components:

$$\hat{H} = \hat{H}_{ads} + \hat{H}_{cat} + \hat{H}_{coupling} \tag{s5}$$

with

$$\hat{H}_{ads} = \varepsilon_a |a\rangle\langle a| \quad \text{(s6)}$$

$$\hat{H}_{cat} = \sum_k \varepsilon_k |k\rangle\langle k| \quad \text{(s7)}$$

$$\hat{H}_{coupling} = \sum_k (V_k |k\rangle\langle a| + V_k^* |a\rangle\langle k|) \quad \text{(s8)}$$

where $\hat{H}_{ads}$ represents the isolated adsorbate, $\hat{H}_{cat}$ accounts for the electronic structure of the catalyst, and the $\hat{H}_{coupling}$ captures the interaction between adsorbate and catalyst. $|a\rangle$ and $|k\rangle$ are the adsorbate state and orthonormal basis for the catalyst states, respectively; ε_a and ε_k are the energy level of adsorbate and each metal states on catalyst; V_k and V_k^* are the coupling matrix between adsorbate and catalyst. Physically, the Hamiltonian must be Hermitian and satisfy the $\hat{H} = \hat{H}^\dagger$, therefore, $\hat{H}_{coupling}$ should be composed by two parts: $V_k |k\rangle\langle a|$ and $V_k^* |a\rangle\langle k|$. In electrocatalysis, the $\sum_k V_k |k\rangle\langle a|$ accounts for the case that taking an electron from the adsorbate $|a\rangle$ and move it into the catalyst $|k\rangle$, while the $\sum_k V_k^* |a\rangle\langle k|$ represents the electron transfer direction from catalyst to adsorbate. Their combination represents the electron donation and back-donation in chemistry. In contrast to the FGR, Eq. (s5) treats coupling non-perturbatively, allowing it to capture the full range of chemisorption, from weak to strong.

To investigate the adsorbate level change with catalyst interaction, we employed the Green's function operator:

$$G(\varepsilon) = \left(\varepsilon + i\eta - \hat{H}\right)^{-1} \quad \text{(s9)}$$

where ε is the energy variable and $\eta \to 0^+$ is a small positive infinitesimal introduced to ensure convergence. The projected Green's function onto the adsorbate is

$$G_a(\varepsilon) = \langle a \left| \left(\varepsilon + i\eta - \hat{H}\right)^{-1} \right| a\rangle \quad \text{(s10)}$$

Define:

$$|G_a\rangle = G(\varepsilon)|a\rangle \quad \text{(s11)}$$

Since the Hilbert space of the system (adsorbate + catalyst) is spanned over the states $|a\rangle$ and all catalyst states $|k\rangle$, we can expand the vector $|G_a\rangle$ as:

$$|G_a\rangle = \alpha|a\rangle + \sum_k \beta_k|k\rangle \tag{s12}$$

Combine the Eqs. (s9) and (s11), we can get:

$$\left(\varepsilon + i\eta - \widehat{H}\right)|G_a\rangle = |a\rangle \tag{s13}$$

Combine the Eqs. (s5-s8) and (s11-s12), then:

$$(\varepsilon + i\eta - \varepsilon_a)\alpha - \sum_k V_k^* \beta_k = 1 \tag{s14}$$

$$(\varepsilon + i\eta - \varepsilon_k)\beta_k - V_k \alpha = 0 \tag{s15}$$

Therefore,

$$\beta_k = \frac{V_k \alpha}{\varepsilon + i\eta - \varepsilon_k} \tag{s16}$$

Insert β_k into Eq. (s14):

$$(\varepsilon + i\eta - \varepsilon_a)\alpha - \sum_k \frac{|V_k|^2 \alpha}{\varepsilon + i\eta - \varepsilon_k} = 1 \tag{s17}$$

$$\alpha = \frac{1}{\varepsilon + i\eta - \varepsilon_a - \sum(\varepsilon)} \tag{s18}$$

where

$$\sum(\varepsilon) = \sum_k \frac{|V_k|^2}{\varepsilon + i\eta - \varepsilon_k} \tag{s19}$$

Thus, the projected Green function is:

$$G_a(\varepsilon) = \langle a|G(\varepsilon)|a\rangle = \frac{1}{\varepsilon + i\eta - \varepsilon_a - \sum(\varepsilon)} \tag{s20}$$

It described how the adsorbate state is modified due to the interaction with catalyst. Therefore, based on the quantum many-body theory, the PDOS on the adsorbate can be obtained from the imaginary part of the projected Green function as:

$$\rho_a(\varepsilon) = -\frac{1}{\pi}\mathrm{Im}G_a(\varepsilon) = \frac{1}{\pi} \cdot \frac{\Delta(\varepsilon)}{\left(\varepsilon - \varepsilon_a - \Lambda(\varepsilon)\right)^2 + \Delta(\varepsilon)^2} \tag{s21}$$

where $\Lambda(\varepsilon)$ and $\Delta(\varepsilon)$ are the real and imaginary part of $\sum(\varepsilon)$ (self-energy), presenting the energy shift and level broadening of adsorbates, respectively. The $\Delta(\varepsilon)$ can be expressed as:

$$\Delta(\varepsilon) = -\mathrm{Im}\left[\sum(\varepsilon)\right] = \pi \sum_k |V_k|^2 \delta(\varepsilon - \varepsilon_k) \tag{s22}$$

For the ideal adsorbate-catalyst systems with weak to moderate coupling, $\Lambda(\varepsilon)$ is mostly negligible, thus Eq. (s21) can be simplified as:

$$\rho_a(\varepsilon) \approx \frac{1}{\pi} \cdot \frac{\Delta(\varepsilon)}{(\varepsilon - \varepsilon_a)^2 + \Delta(\varepsilon)^2} \tag{s23}$$

This result takes the Lorentzian form:

$$L(x) = \frac{1}{\pi} \cdot \frac{\gamma}{(x - x_0)^2 + (\gamma)^2} \tag{s24}$$

where γ is the width at half maximum of Lorentzian.

Therefore, we can relate peak broadening to hopping rate, if the adsorbate level ε_a is aligned with the Fermi level ε_F. Then, the peak width at ε_F reflects the coupling strength, and the electron hopping rate expression becomes:

$$k = \frac{2}{\hbar} \Delta(\varepsilon_F) \tag{s25}$$

where $\Delta(\varepsilon_F)$ is the width at half maximum of the PDOS peak evaluated at ε_F.

In this derivation based on the NA model, two key assumptions are made to arrive at a simplified expression for the electron hopping rate. First, the coupling between the localized adsorbate orbital and the catalyst states is assumed to be weak to moderate, allowing the real part of the hybridization function ($\Lambda(\varepsilon)$) to be neglected. Second, the analysis focuses on a single localized orbital near the ε_F, assuming it dominates the electron transfer process and neglecting contributions from other orbitals or thermal activated transitions.

Under these assumptions, the electron hopping rate simplifies to Eq. (s25). However, this simplified expression is only valid when the center of adsorbate DOS peak aligns with ε_F. When the peak is shifted away from the ε_F while still overlapping via its tail, the simplified formula significantly overestimates the actual hopping rate. In addition, this expression cannot be applied to systems with semiconducting adsorbates, where ε_F lies within a band gap and no available DOS exists to support direct hopping. These limitations motivate us to develop a more generalized framework to describe electron transfer in such off-resonant or gapped systems.

2.3 Energy-shift-corrected electron hopping rate

To address the limitation of the standard hopping rate expression, for systems where the adsorbate DOS is not centered at the Fermi level, we re-consider the effect of energy shift on the electron transfer process. In more general cases, the center of projected DOS on adsorbate may not be fully aligned with ε_F, but instead appears as a broadened peak centered at an energy ε_0 with an energy shift Λ from the ε_F:

$$\varepsilon_F = \varepsilon_0 + \Lambda \tag{s26}$$

For a Lorentzian-shaped DOS with maximum height ρ_0 and half-width Δ, the DOS form can be re-written as:

$$\rho(\varepsilon) = \frac{\rho_0 \cdot \Delta^2}{(\varepsilon - \varepsilon_0)^2 + \Delta^2} \tag{s27}$$

the value of the DOS at the Fermi level is then given by:

$$\rho(\varepsilon_F) = \frac{\rho_0 \cdot \Delta^2}{\Lambda^2 + \Delta^2} \tag{s28}$$

This result highlights that even when the tail of the adsorbate DOS overlaps with ε_F, the actual DOS at the ε_F is significantly reduced compared to the case with $\Lambda = 0$. Consequently, the electron hopping rate is suppressed in proportion to this reduced overlap. Substituting $\rho(\varepsilon_F)$ into the FGR expression, we can renew it as:

$$k = \frac{2\pi}{\hbar}|V|^2 \rho(\varepsilon_F) = \frac{2\pi}{\hbar}|V|^2 \frac{\rho_0 \cdot \Delta^2}{\Lambda^2 + \Delta^2} \tag{s29}$$

Alternatively,

$$\Delta(\varepsilon) = \pi |V|^2 \rho_0 \tag{s30}$$

The final expression of electron hopping rate can be re-written as:

$$k = \frac{2}{\hbar}\Delta \cdot \frac{\Delta^2}{\Lambda^2 + \Delta^2} \tag{s31}$$

This generalized expression reduces to the standard result Eq. (s25) when $\Lambda = 0$, and captures the suppression of hopping that occurs as the adsorbate state becomes energetically misaligned with ε_F.

Compared with the full derivation above, the electron hopping rate can also be expressed in a more compact but physically transparent form as:

$$k = \frac{2}{\hbar}\Delta \cdot \frac{\rho(\varepsilon_F)}{\rho_0} \tag{s32}$$

where $\rho(\varepsilon_F)$ is the adsorbate PDOS directly evaluated at the Fermi level and ρ_0 is the maximum value of the adsorbate peak.

Combine with an ideal Lorentzian line shape:

$$\frac{\rho(\varepsilon_F)}{\rho_0} = \frac{\Delta^2}{\Lambda^2 + \Delta^2} \tag{s33}$$

Eq. (s32) can reduce to Eq. (s31). More generally, Eq. (s32) remains valid for non-ideal Lorentzian line shapes, in which the ratio $\frac{\rho(\varepsilon_F)}{\rho_0}$ can be directly obtained from DFT-calculated PDOS without assuming any specific functional form. Therefore, it provides a simpler and more model-independent correction in actual calculations.

2.4 Semiconducting electron hopping rate

The derivation above assumes a localized adsorbate orbital with finite DOS at the ε_F, allowing direct electron hopping. However, in systems where the adsorbate is semiconducting, the DOS vanishes at the ε_F, and direct hopping is suppressed. In such cases, electron transport must proceed through a thermally activated mechanism, which therefore requires an improved rate expression that includes thermal effects.

To account for the thermal effect, we extend the FGR integrating over the energy spectrum with incorporating the Fermi-Dirac distribution, defined as:

$$k = g \cdot \frac{2\pi}{\hbar} \int_{-\infty}^{+\infty} |V_\varepsilon|^2 \rho(\varepsilon) f(\varepsilon, T) d_\varepsilon \tag{s34}$$

where $g = 2$ is the spin degeneracy factor, and $f(\varepsilon, T)$ is the Fermi-Dirac distribution:

$$f(\varepsilon, T) = \frac{1}{e^{\varepsilon-\mu/k_B T} + 1} \tag{s35}$$

Here, μ is the chemical potential of electrons and T is the temperature. In the metallic limit, where $\rho(\varepsilon_F)$ is non-zero, Eq. (s34) reduces to:

$$k \approx \frac{2\pi}{\hbar} |V|^2 \rho(\varepsilon_F) \tag{s36}$$

recovering the expression of Eq. (s4). However, in the semiconducting case, the integral is

dominated by the tail of the valence band close to ε_F, requiring thermal excitation across the band gap ΔE. Assuming the 1st peak below ε_F is centered at the valence band maximum (VBM), the electron hopping rate can be renewed as:

$$k \approx \frac{2}{\hbar}\Delta(\varepsilon_{VBM}) \cdot e^{-\frac{\Delta E}{k_B T}} \cdot k_B T \tag{s37}$$

which follows Arrhenius-type behavior:

$$k \propto e^{-\frac{\Delta E}{k_B T}} \tag{s38}$$

Therefore, the electron hopping rate can be summarized as:

$$k = \begin{cases} \frac{2}{\hbar}\Delta \cdot \frac{\rho(\varepsilon_F)}{\rho_0}, & metallic \\ \frac{2}{\hbar}\Delta(\varepsilon_{VBM}) \cdot e^{-\frac{\Delta E}{k_B T}} \cdot k_B T, & semiconducting \end{cases} \tag{s39}$$

Part 3. Figures and tables of the manuscript

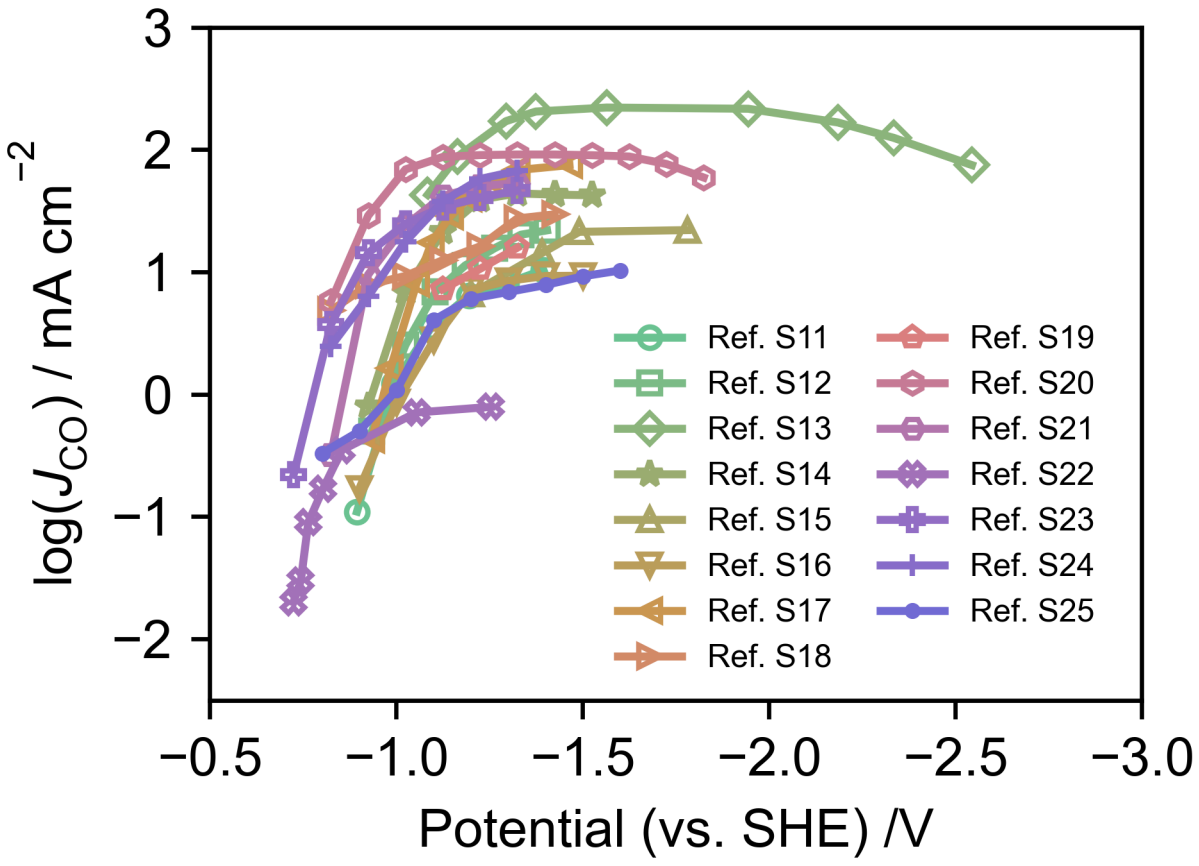

Figure S1. Experimental data set of CO partial current density as a function of applied potential (all converted to values versus SHE to enable comparison across different pH conditions).[11-25] Electrolyte and pH conditions in references are (1) 0.1 M $KHCO_3$ with pH 6.8: Ref. S15, S16, and S25; (2) 0.5 M $KHCO_3$ with pH 7.2: Ref. S11, S12, S14, S17-S21, S23, and S24; (3) 1 M $KHCO_3$ with pH 7.7: Ref. S13, and S22.

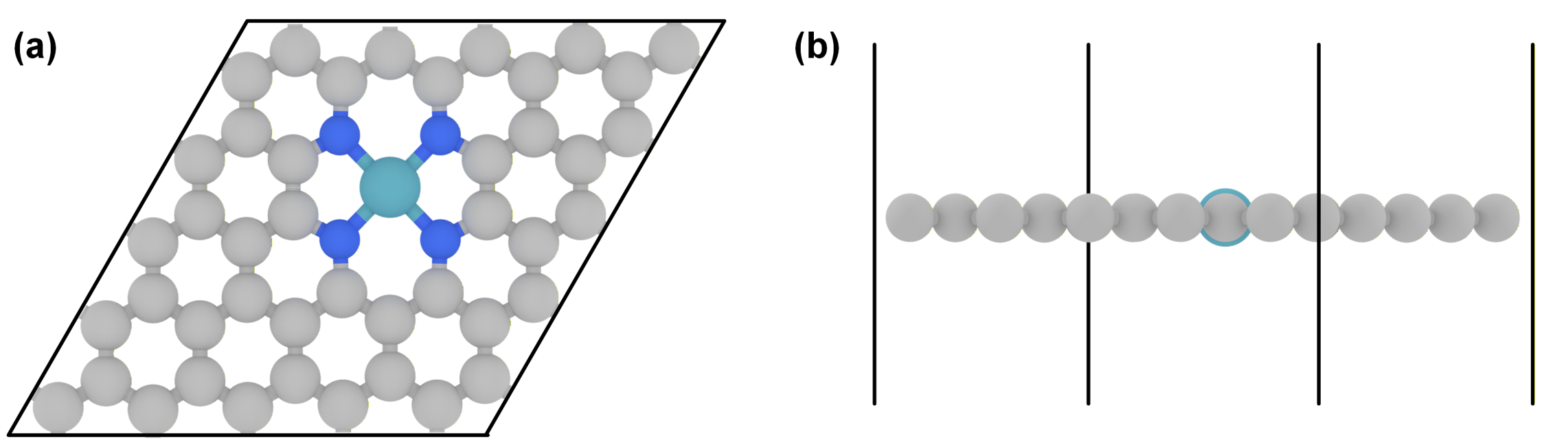

Figure S2. Structure model of TM-N_4 SACs. (a) Top view. (b) Side view. The C, N, and transition metal (TM) atoms are colored in gray, blue, and cyan, respectively.

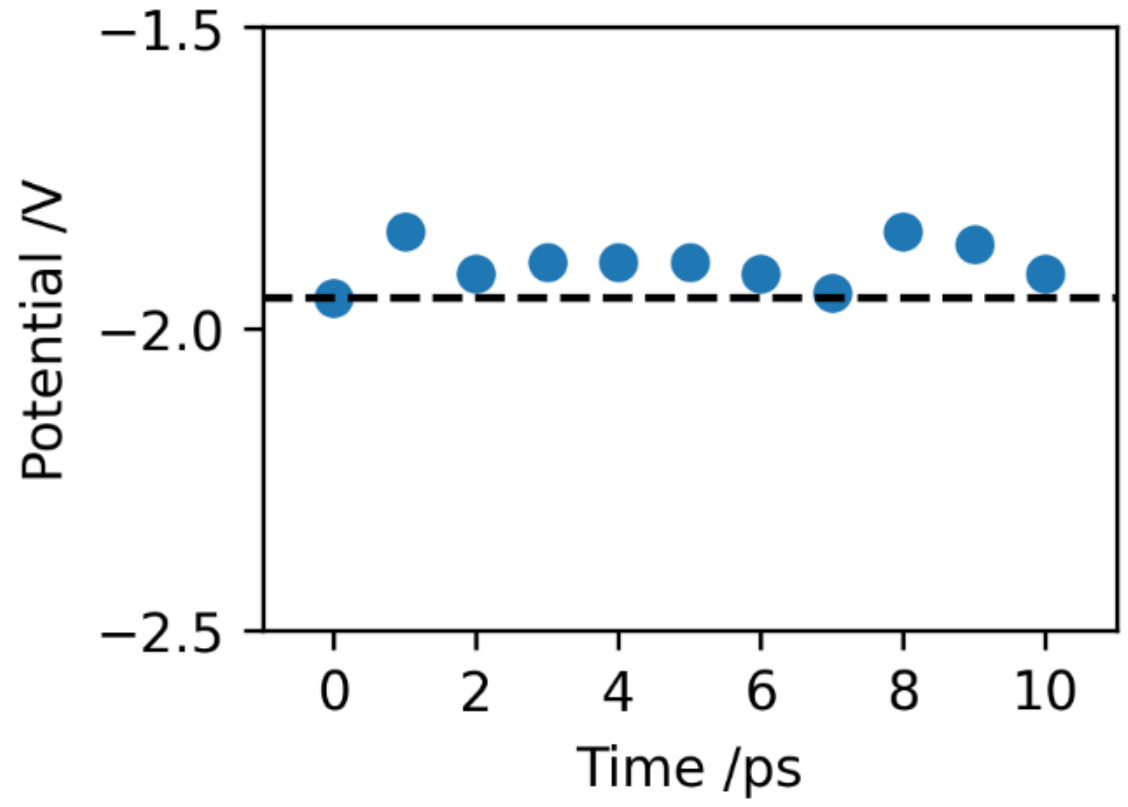

Figure S3. Validation of the fixed-number-electron AIMD treatment for chemisorbed CO_2 on Ni-N_4 at U_{trans}= -1.95 V. Eleven snapshots, extracted at 1 ps intervals from a 10 ps AIMD trajectory, were used to evaluate the corresponding electrode potential. The inferred potential remains close to the target value, fluctuating only within -1.95 to -1.84 V, which supports the use of the CP-derived charge state for assessing the finite-temperature stability of the chemisorbed configuration.

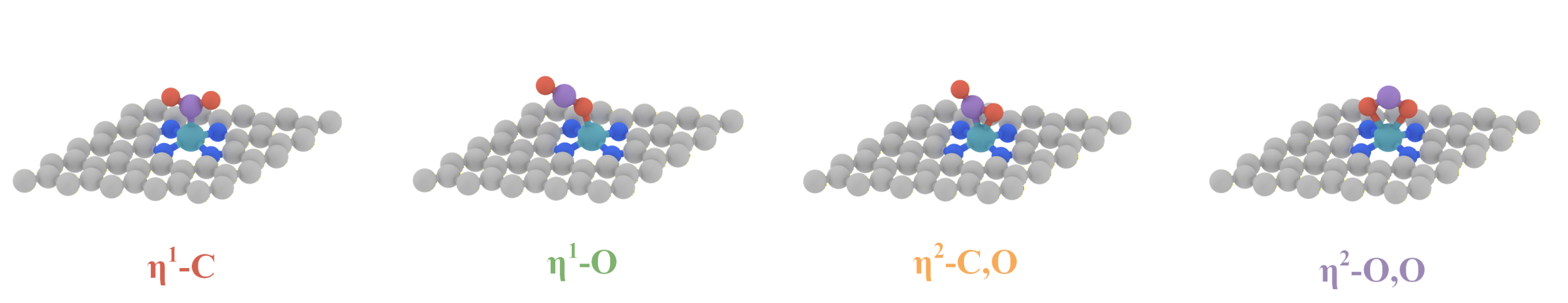

Figure S4. Four possible CO_2 chemisorption configurations, including η^1-C, η^1-O, η^2-C,O, and η^2-O,O. The C atoms in the graphene substrate are colored gray, while those in CO_2 are rendered in purple. The N, O, and TM atoms are shown in blue, red, and cyan, respectively.

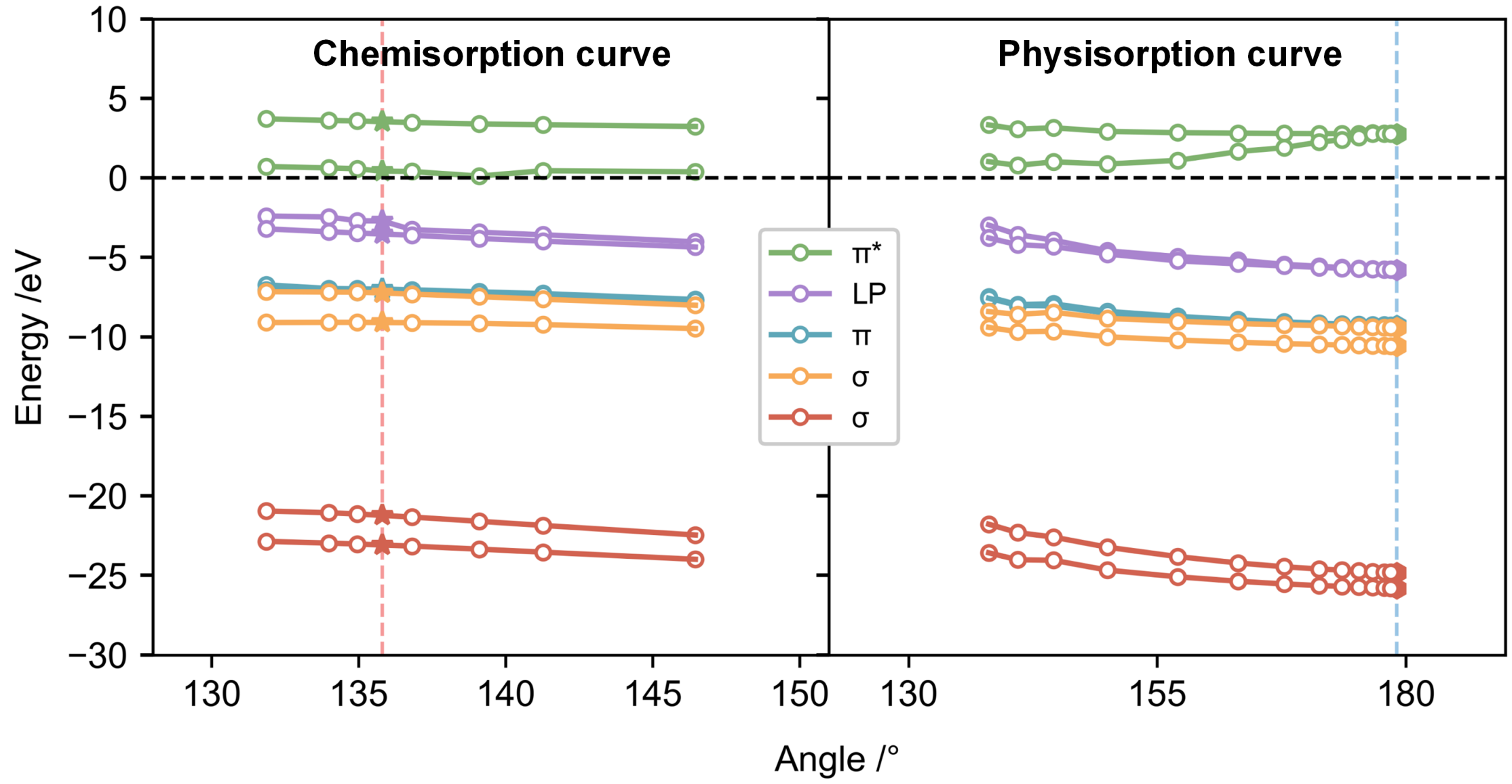

Figure S5. Walsh diagram for CO_2 at U = -1.5 V vs. SHE on Ni-N_4 SAC. The curve for the π* orbitals with relatively lower energy is the average value of multiple states straddling the Fermi level.

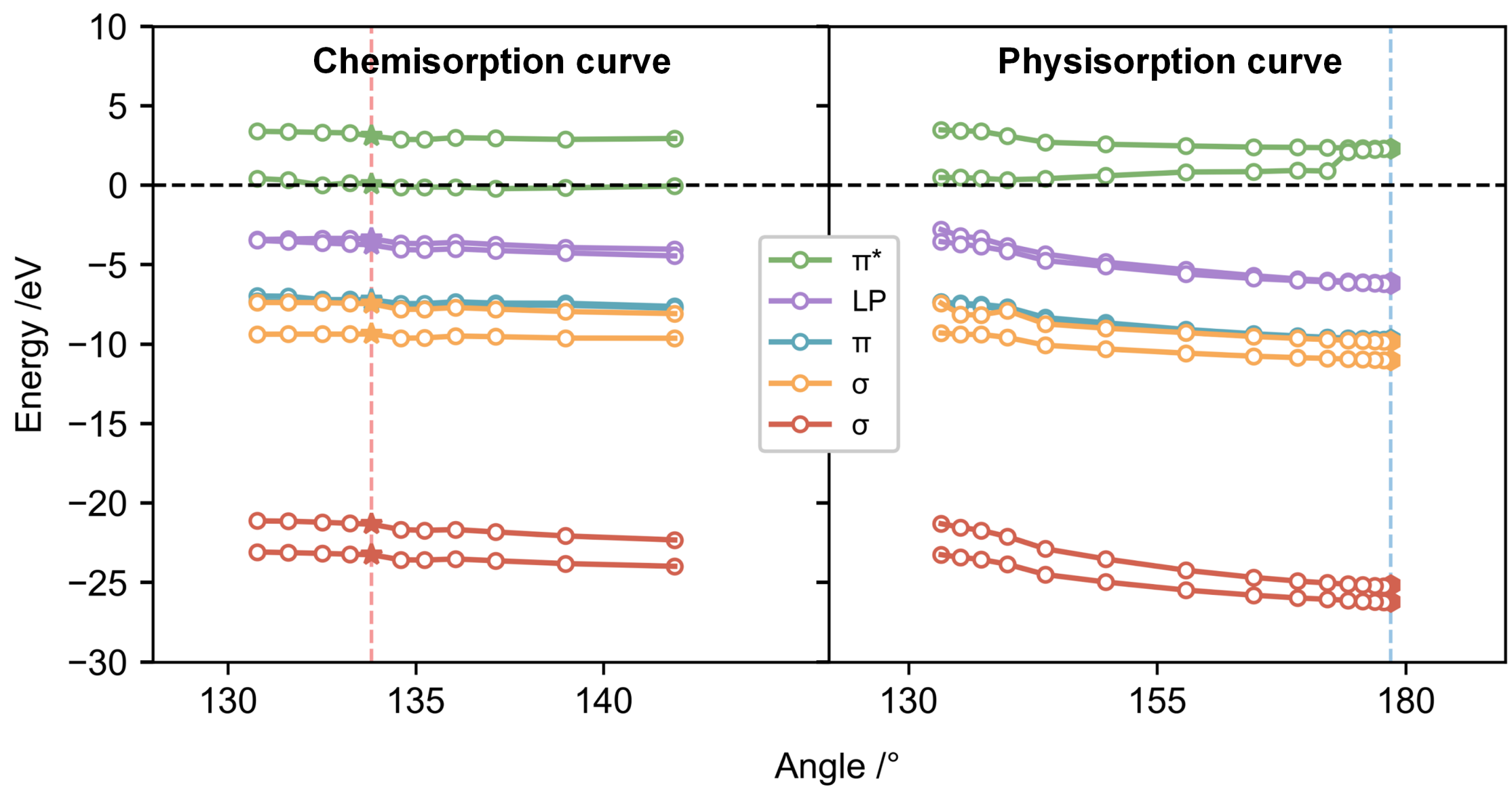

Figure S6. Walsh diagram for CO_2 at U = -2.0 V vs. SHE on Ni-N_4 SAC. The curve for the π* orbitals with relatively lower energy is the average value of multiple states straddling the Fermi level.

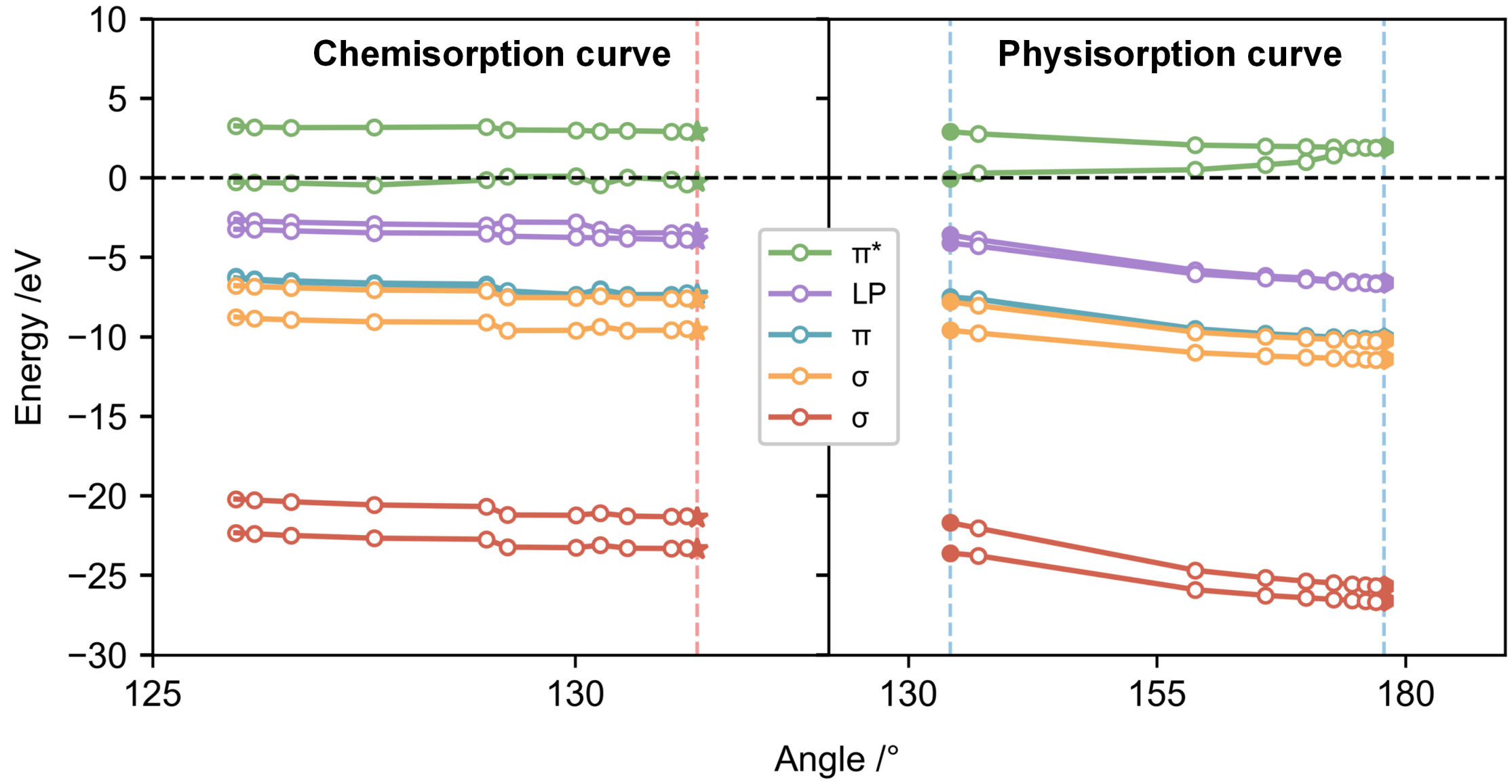

Figure S7. Walsh diagram for CO_2 at U = -2.5 V vs. SHE on Ni-N_4 SAC. The curve for the π* orbitals with relatively lower energy is the average value of multiple states straddling the Fermi level.

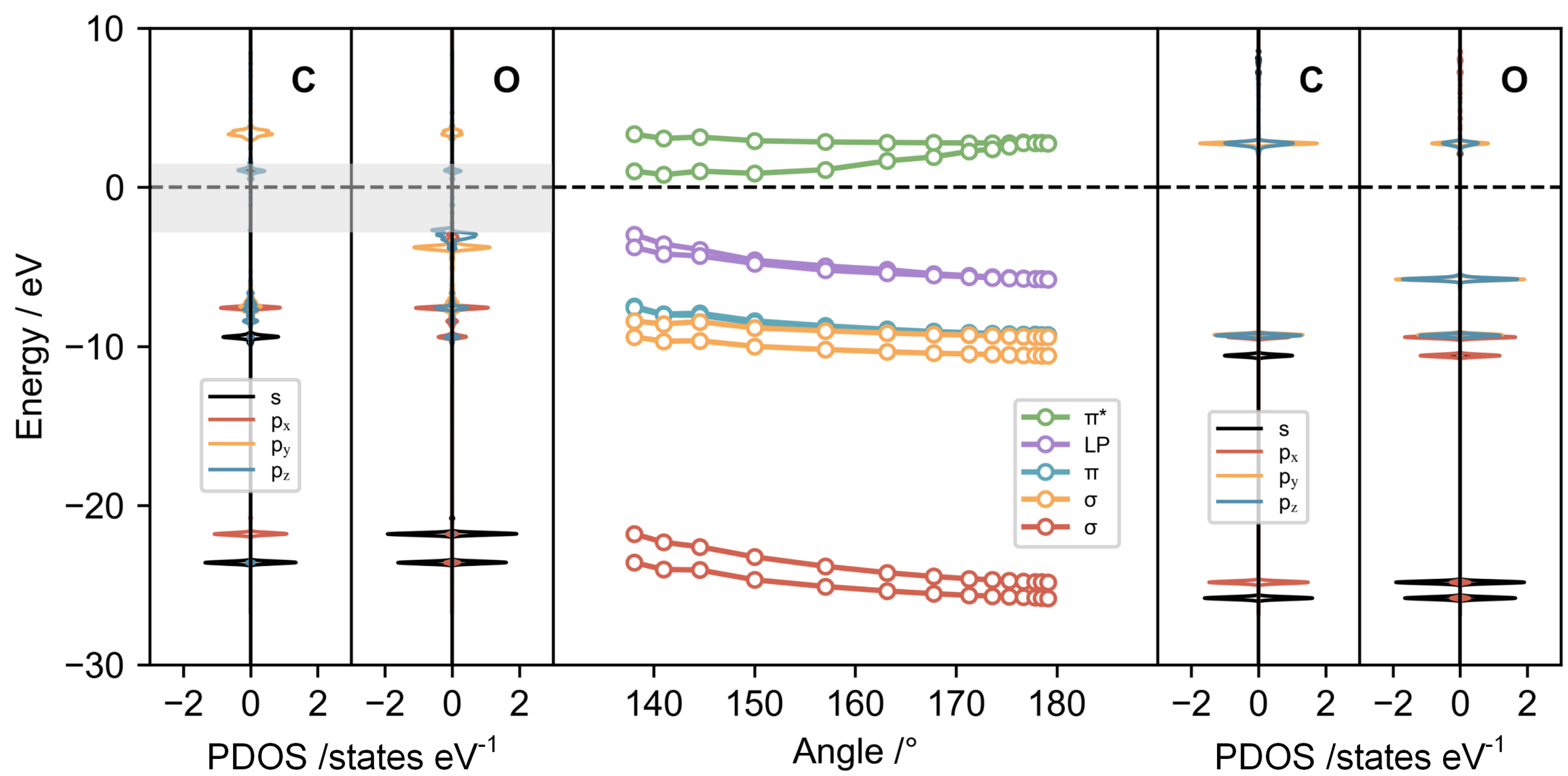

Figure S8. Walsh diagram with corresponding partial density (PDOS) of states of CO_2 along the physisorption curve at U = -1.5 V vs. SHE on Ni-N_4 SAC. The multiple lower-energy π* states are located in the gray ribbon.

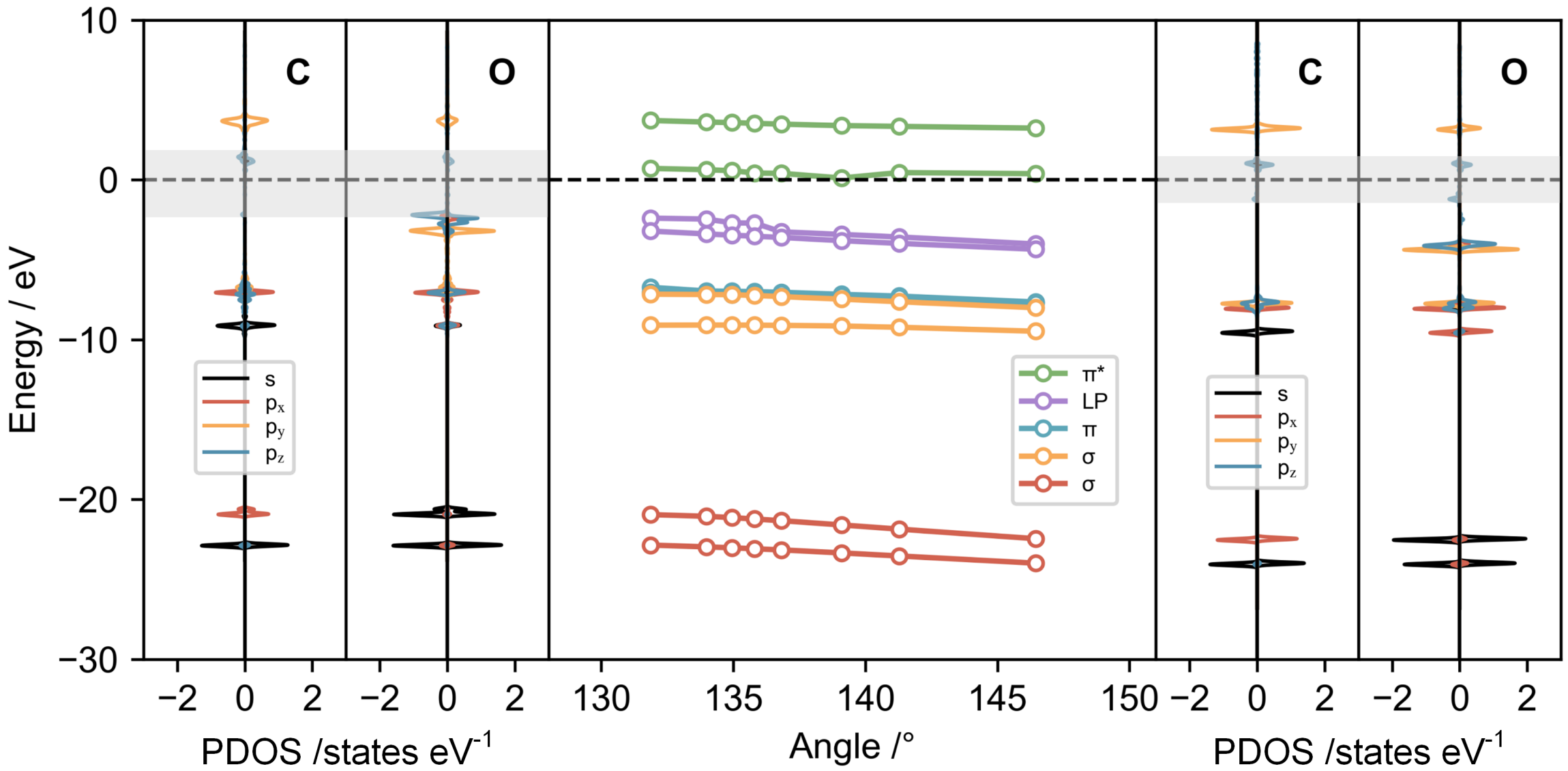

Figure S9. Walsh diagram with corresponding partial density (PDOS) of states of CO_2 along the chemisorption curve at U = -1.5 V vs. SHE on Ni-N_4 SAC. The multiple lower-energy π* states are located in the gray ribbon.

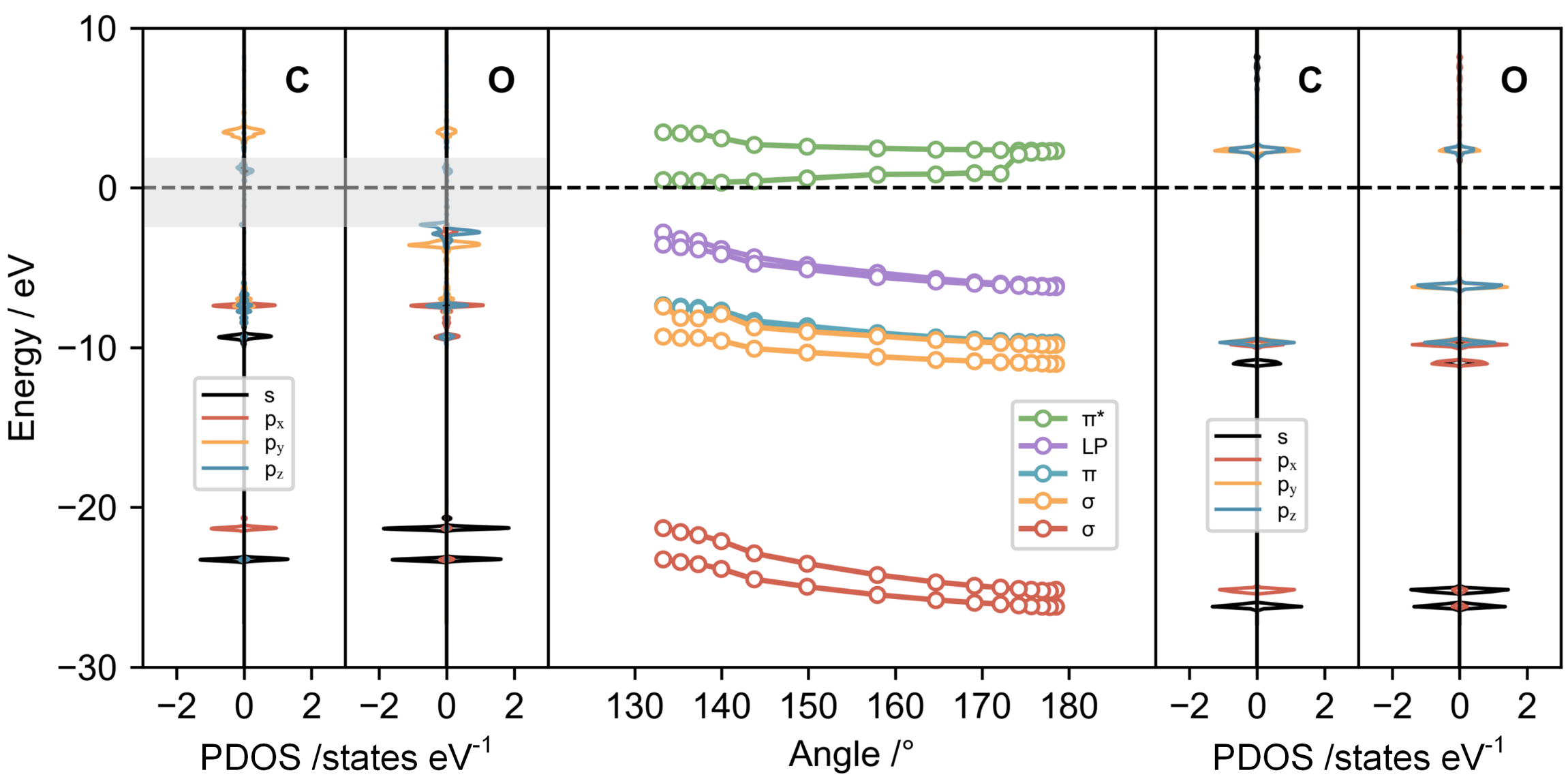

Figure S10. Walsh diagram with corresponding partial density (PDOS) of states of CO_2 along the physisorption curve at U = -2.0 V vs. SHE on Ni-N_4 SAC. The multiple lower-energy π* states are located in the gray ribbon.

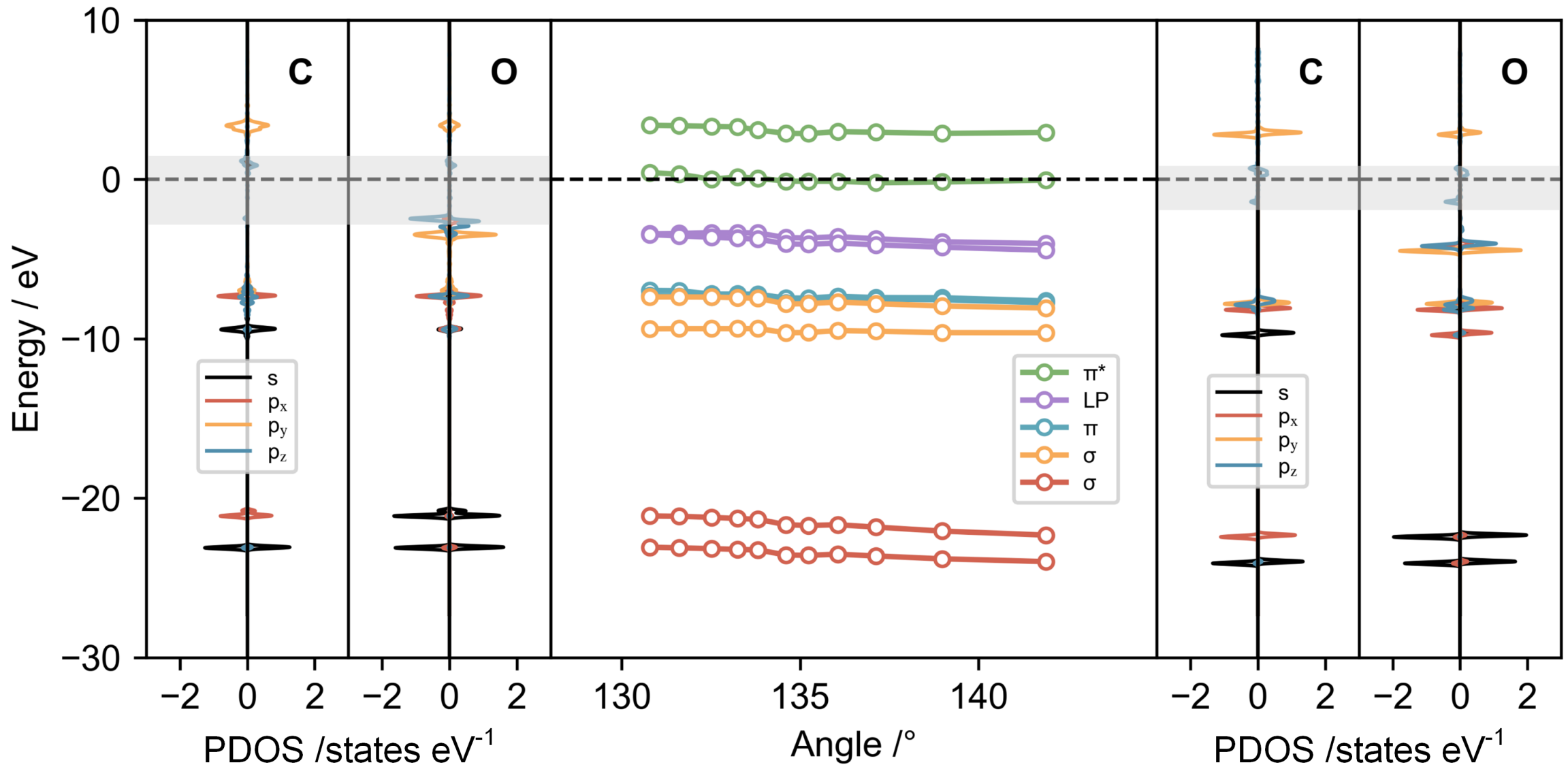

Figure S11. Walsh diagram with corresponding partial density (PDOS) of states of CO_2 along the chemisorption curve at U = -2.0 V vs. SHE on Ni-N_4 SAC. The multiple lower-energy π* states are located in the gray ribbon.

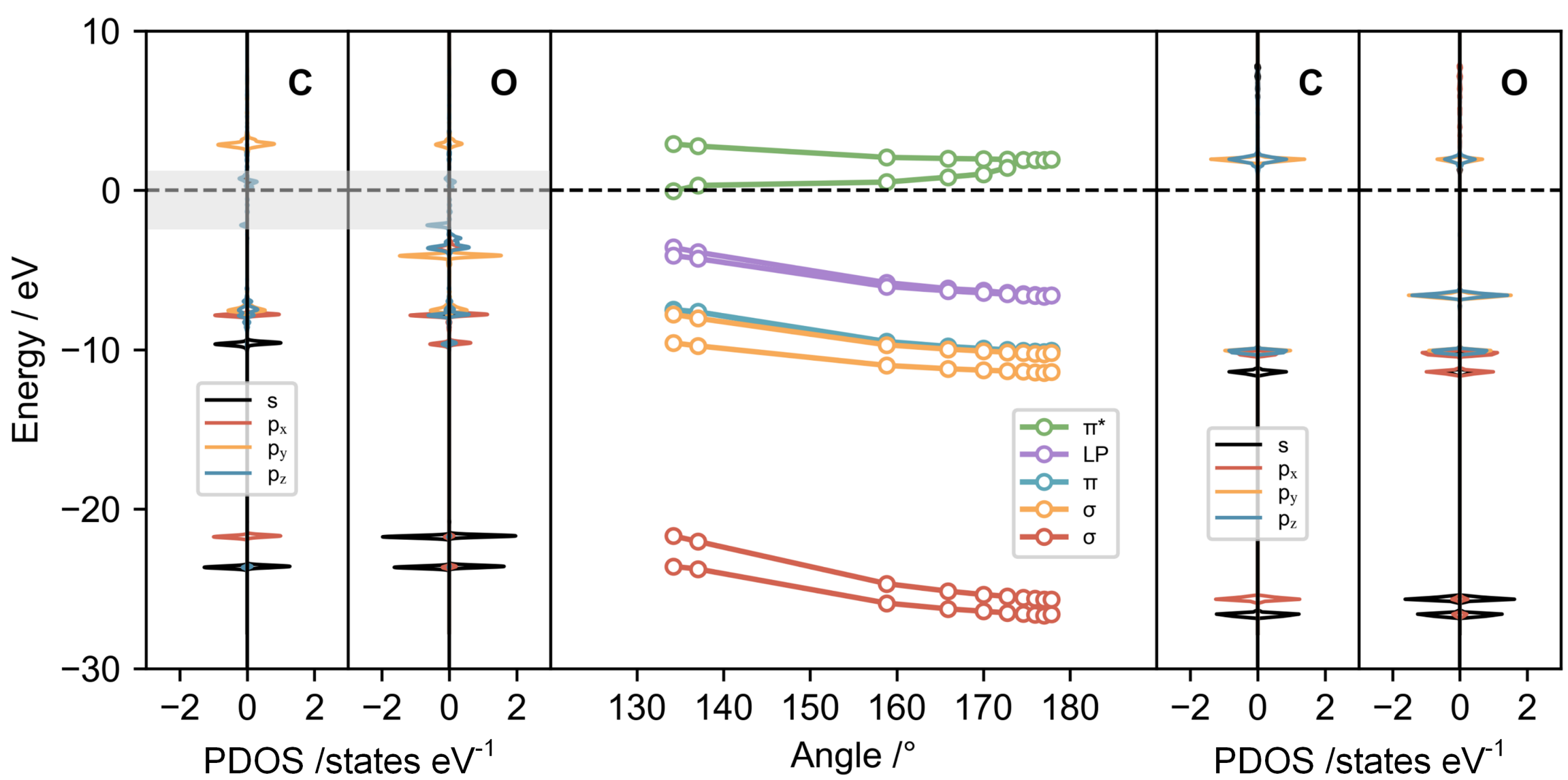

Figure S12. Walsh diagram with corresponding partial density (PDOS) of states of CO_2 along the physisorption curve at U = -2.5 V vs. SHE on Ni-N_4 SAC. The multiple lower-energy π* states are located in the gray ribbon.

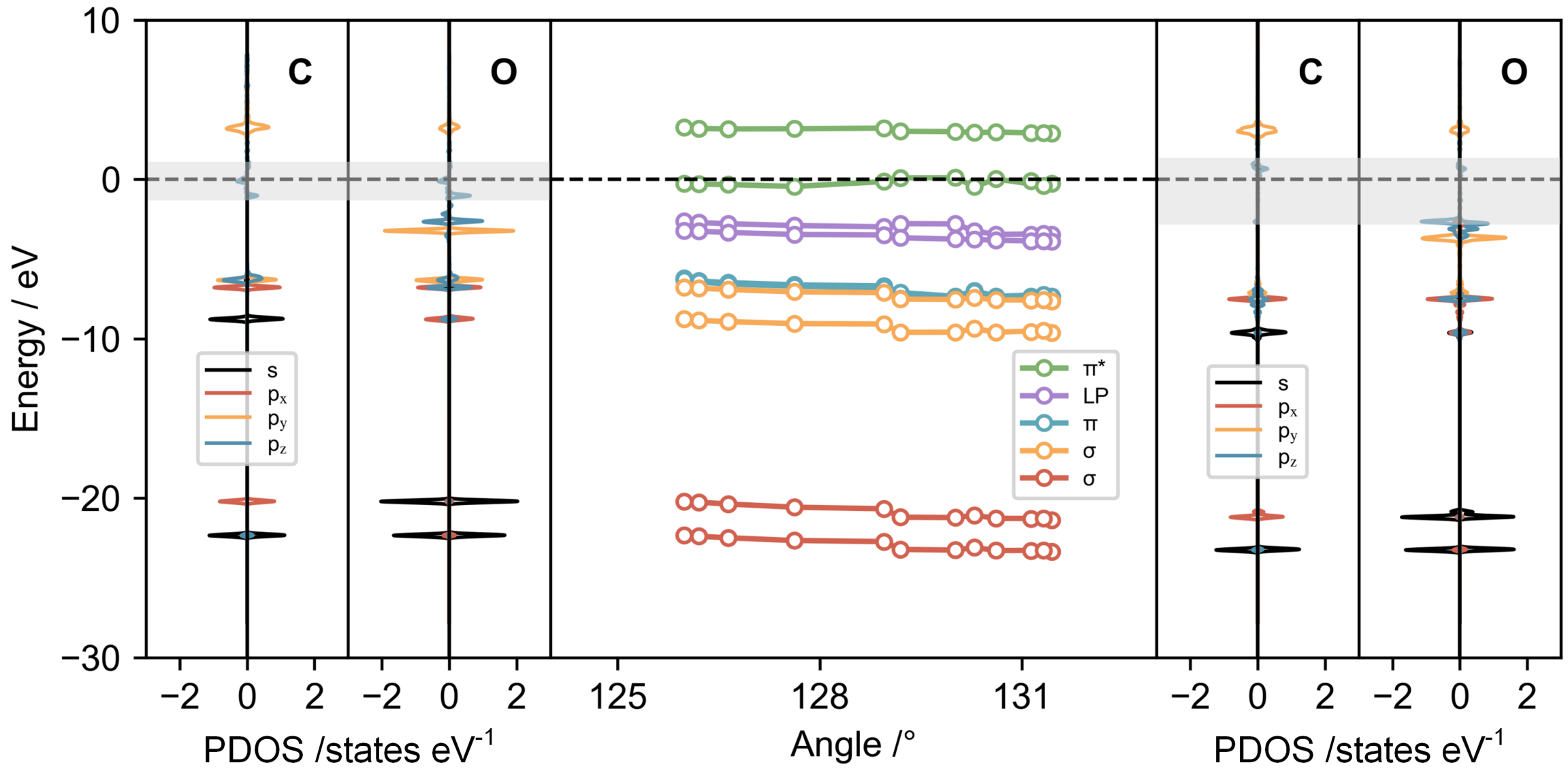

Figure S13. Walsh diagram with corresponding partial density (PDOS) of states of CO_2 along the chemisorption curve at U = -2.5 V vs. SHE on Ni-N_4 SAC. The multiple lower-energy π* states are located in the gray ribbon.

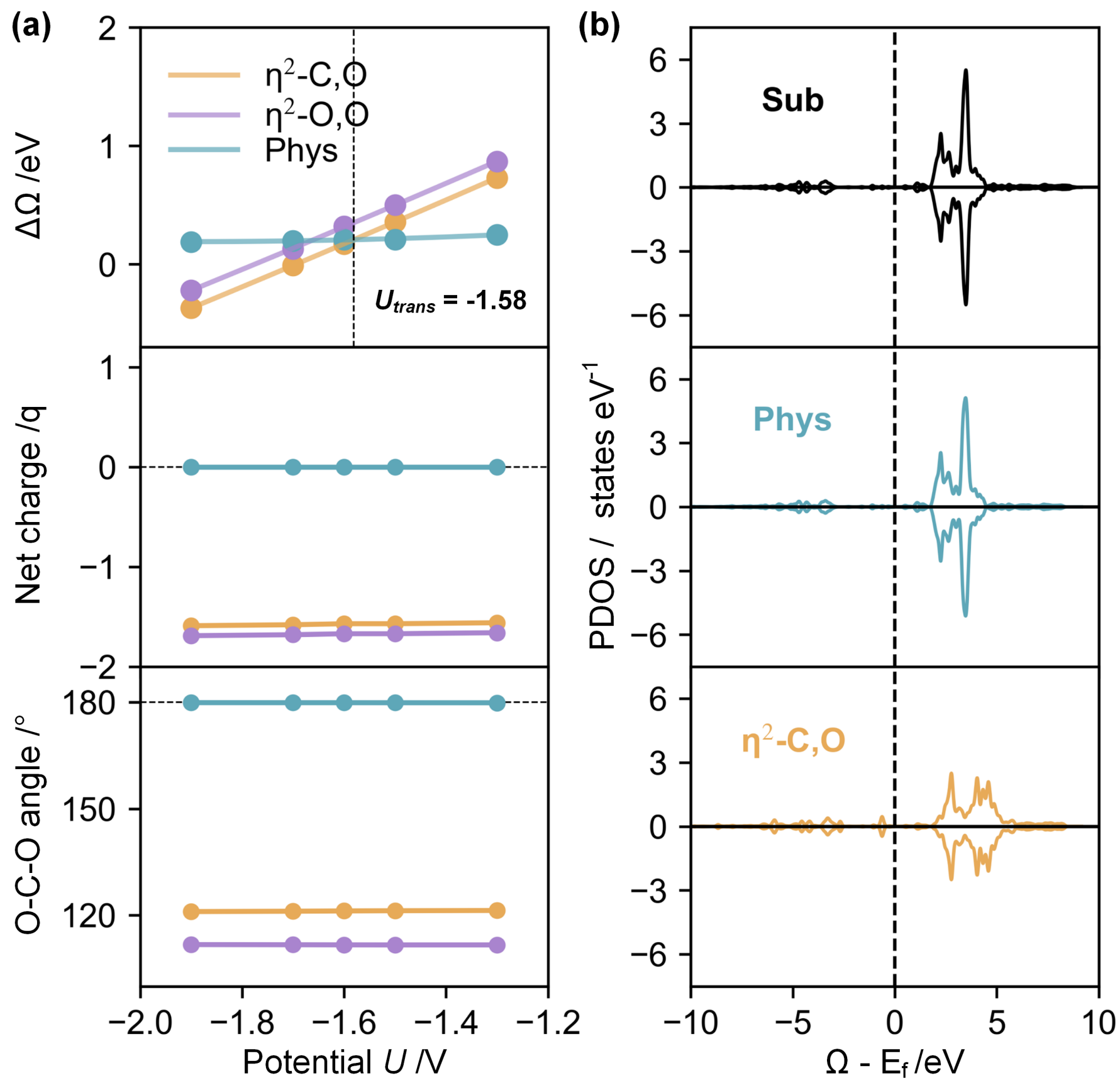

Figure S14. (a) Adsorption energy diagram, net charge transfer, and CO_2 angle variation for the minimum position on the physisorption and chemisorption curves on Sc-N_4 SAC as a function of applied potential U. For the Sc-N_4 SAC, CO_2 chemisorption can occur in η^2-C,O and η^2-O,O configurations, while the η^1-C and η^1-O modes transition to the η^2-C,O or physisorption after geometry optimization. (b) Partial density of states (PDOS) of the Sc active center before and after CO_2 physisorption/chemisorption at "transorption" potential (U_{trans}).

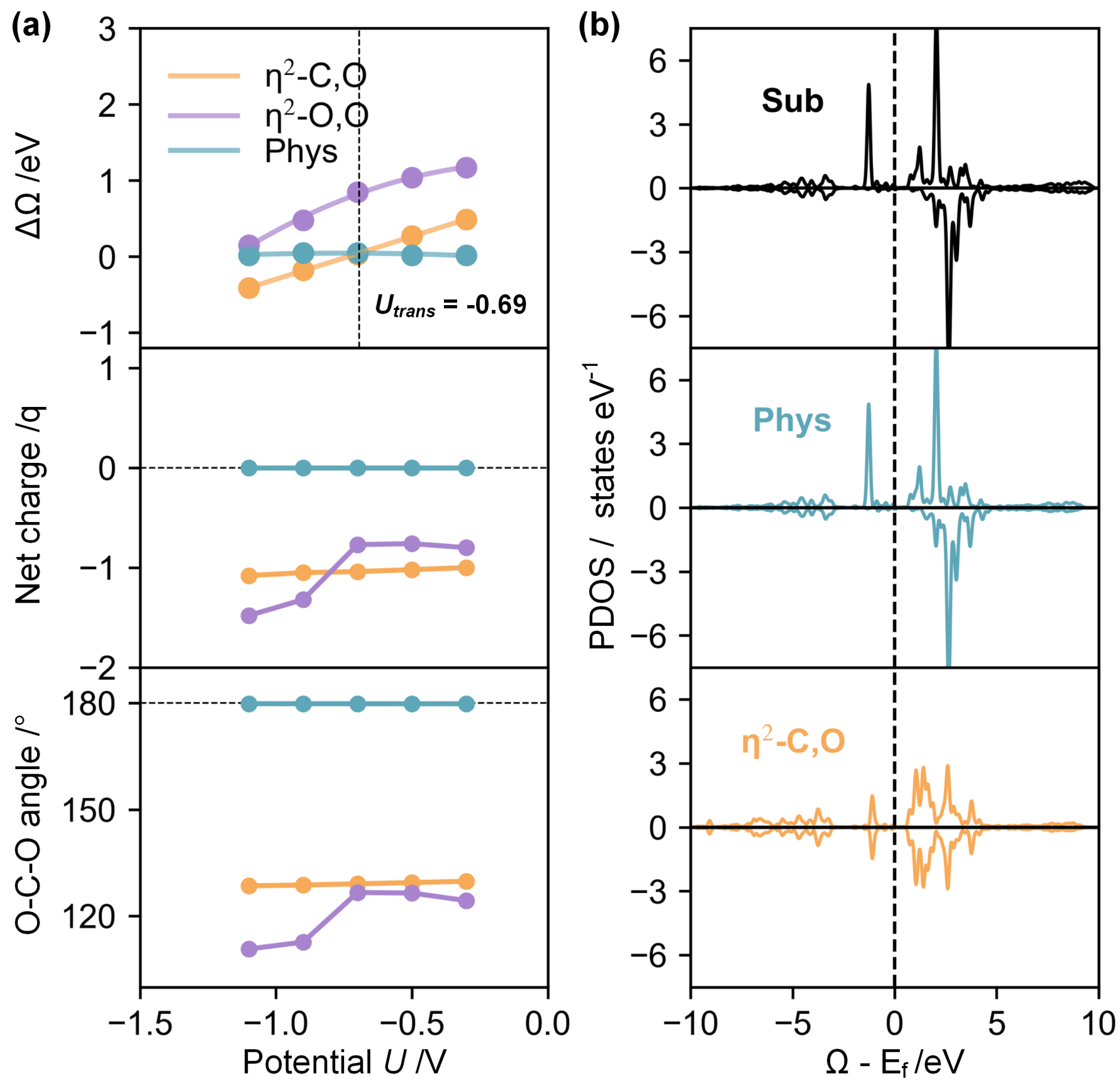

Figure S15. (a) Adsorption energy diagram, net charge transfer, and CO_2 angle variation for the minimum position on the physisorption and chemisorption curves on Ti-N_4 SAC as a function of applied potential U. For the Ti-N_4 SAC, CO_2 chemisorption can occur in η^2-C,O and η^2-O,O configurations, while the η^1-C and η^1-O modes transition to the η^2-C,O or physisorption after geometry optimization. (b) Partial density of states (PDOS) of the Ti active center before and after the CO_2 physisorption/chemisorption at U_{trans}.

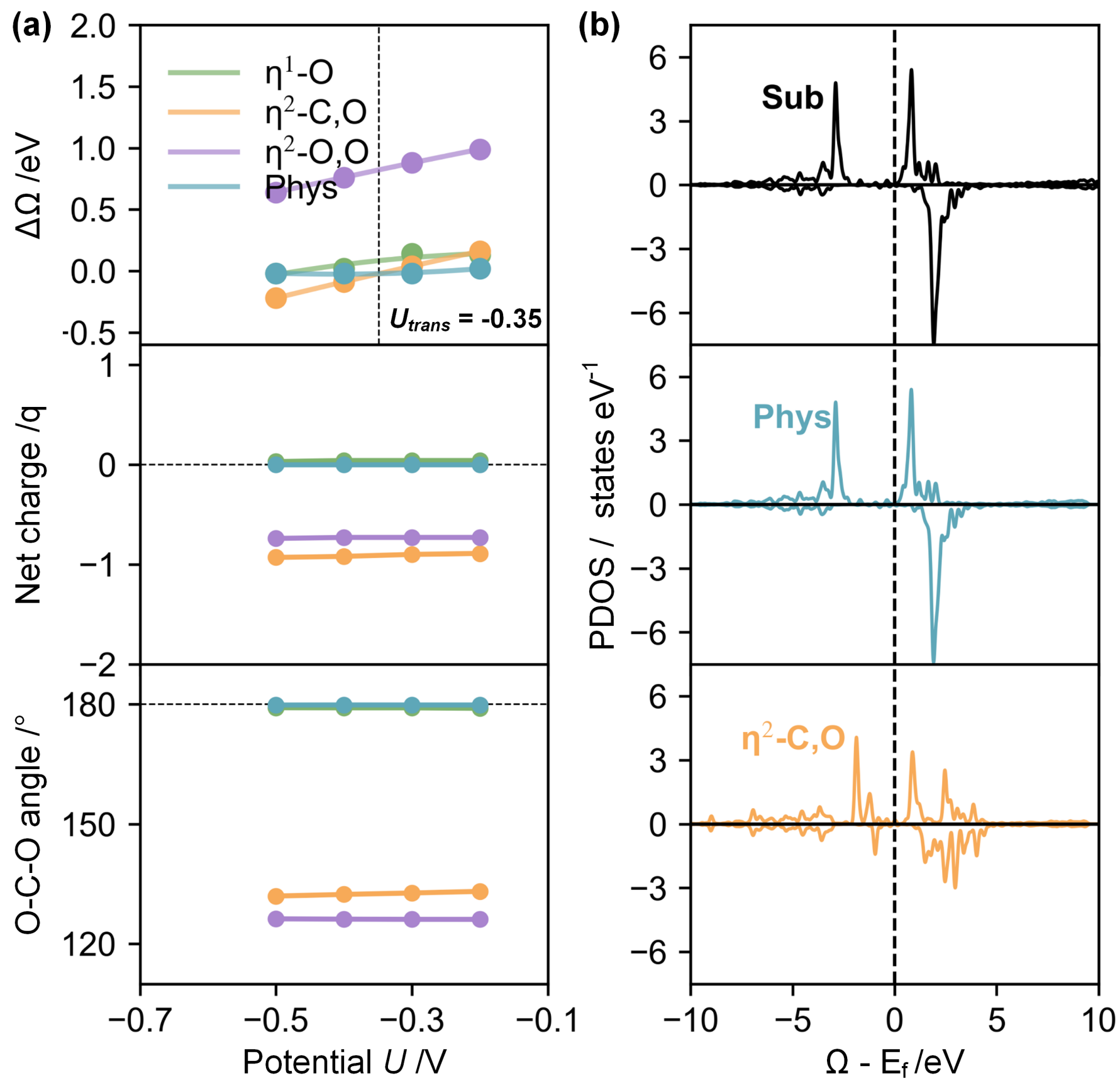

Figure S16. (a) Adsorption energy diagram, net charge transfer, and CO_2 angle variation for the minimum position on the physisorption and chemisorption curves on V-N_4 SAC as a function of applied potential *U*. For the V-N_4 SAC, CO_2 chemisorption can occur in η^1-O, η^2-C,O and η^2-O,O configurations, while the η^1-C mode transitions to the η^2-C,O or physisorption after geometry optimization. (b) Partial density of states (PDOS) of the V active center before and after the CO_2 physisorption/chemisorption at U_{trans}.

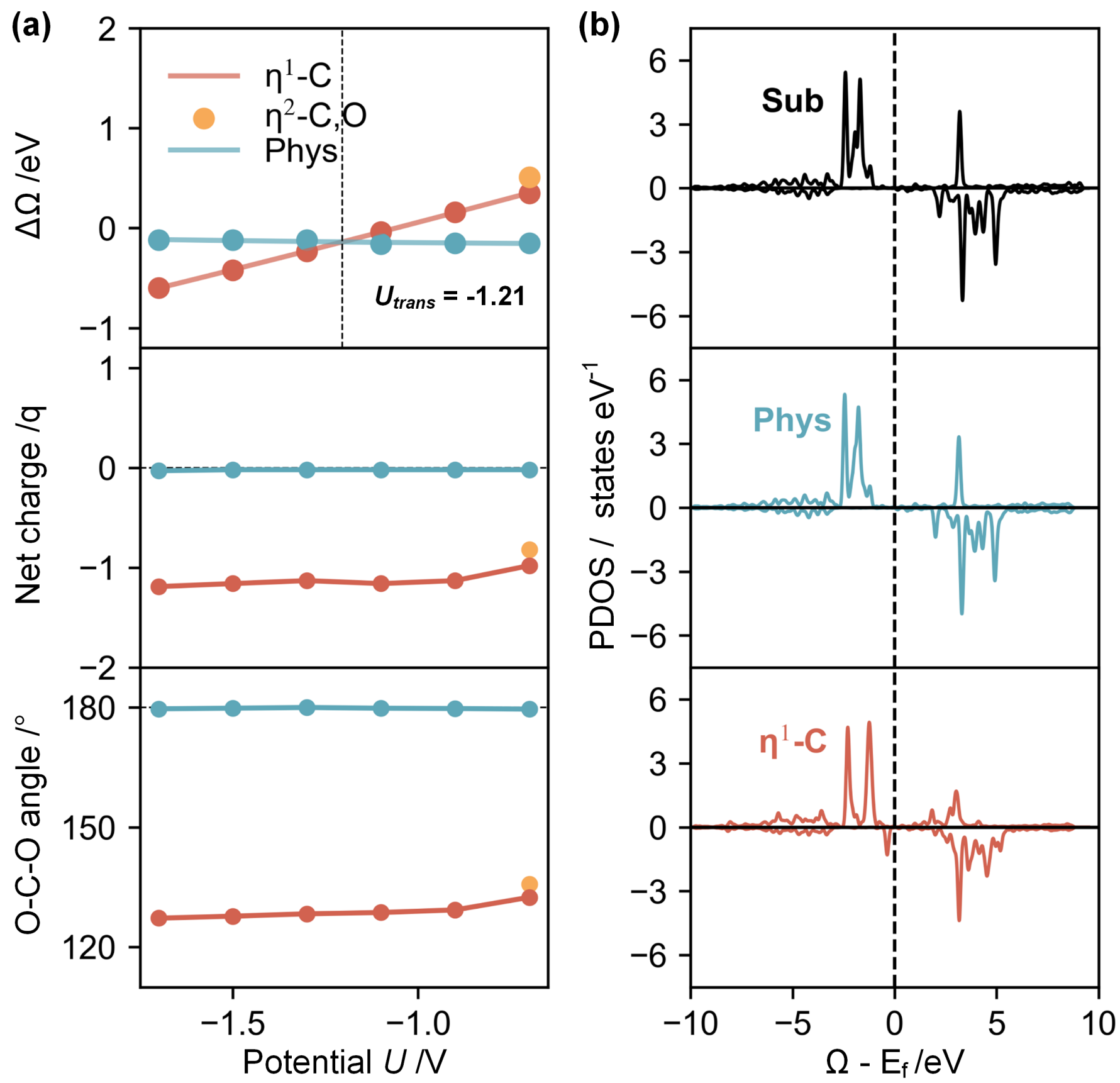

Figure S17. (a) Adsorption energy diagram, net charge transfer, and CO_2 angle variation for the minimum position on the physisorption and chemisorption curves on Cr-N_4 SAC as a function of applied potential *U*. For the Cr-N_4 SAC, CO_2 chemisorption mainly occurs in η^1-C configuration with our examined potential range and the η^2-C,O configuration is the only one other chemisorption mode observed at -0.7 V, while the different initial modes transition to the η^1-C or physisorption at various applied potentials after geometry optimization. (b) Partial density of states (PDOS) of the Cr active center before and after the CO_2 physisorption/chemisorption at U_{trans}.

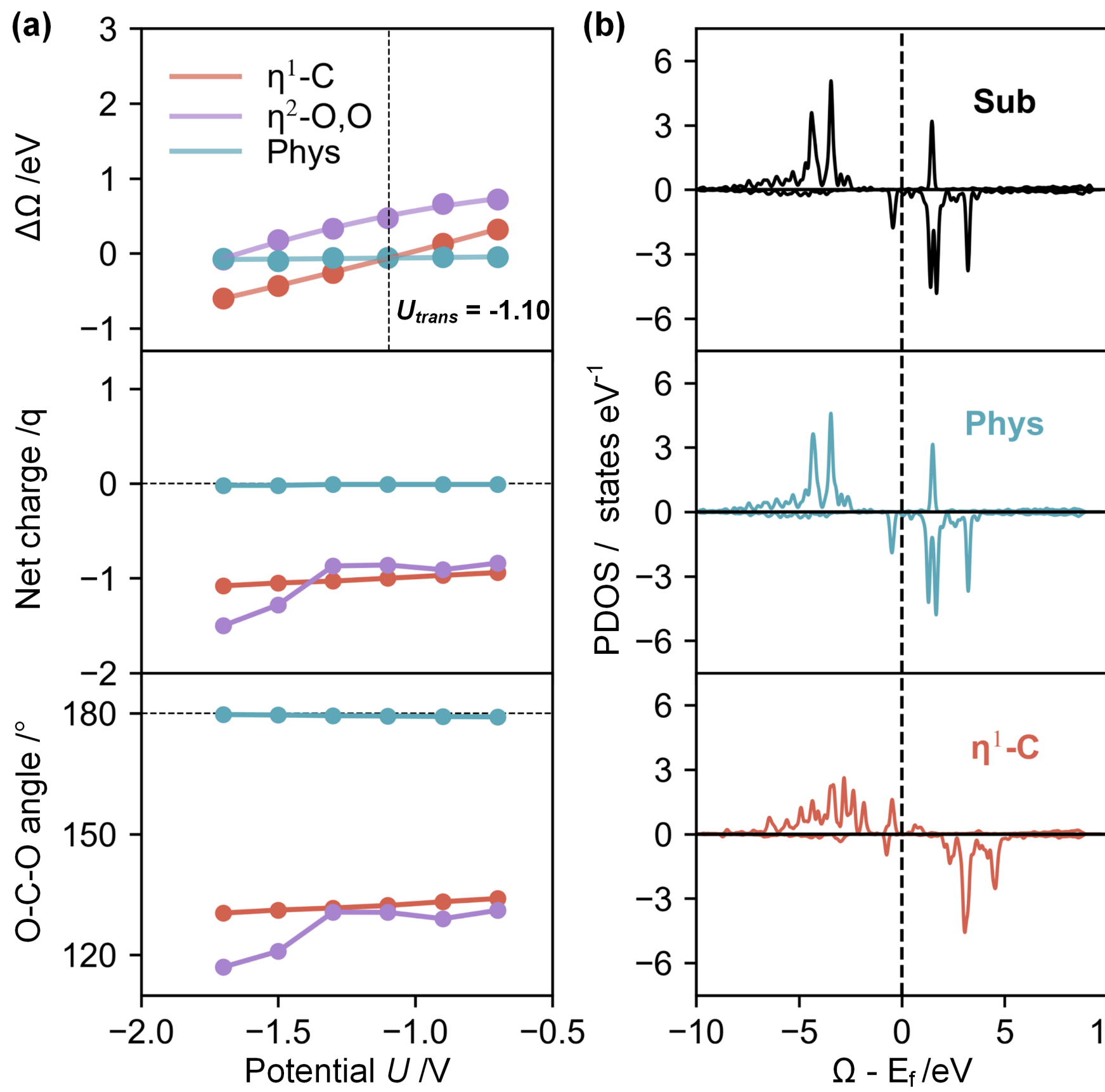

Figure S18. (a) Adsorption energy diagram, net charge transfer, and CO_2 angle variation for the minimum position on the physisorption and chemisorption curves on Mn-N_4 SAC as a function of applied potential U. For the Mn-N_4 SAC, CO_2 chemisorption can occur in η^1-C and η^2-O,O configurations, while the η^1-O and η^2-C,O modes transition to the η^1-C or physisorption after geometry optimization. (b) Partial density of states (PDOS) of the Mn active center before and after the CO_2 physisorption/chemisorption at U_{trans}.

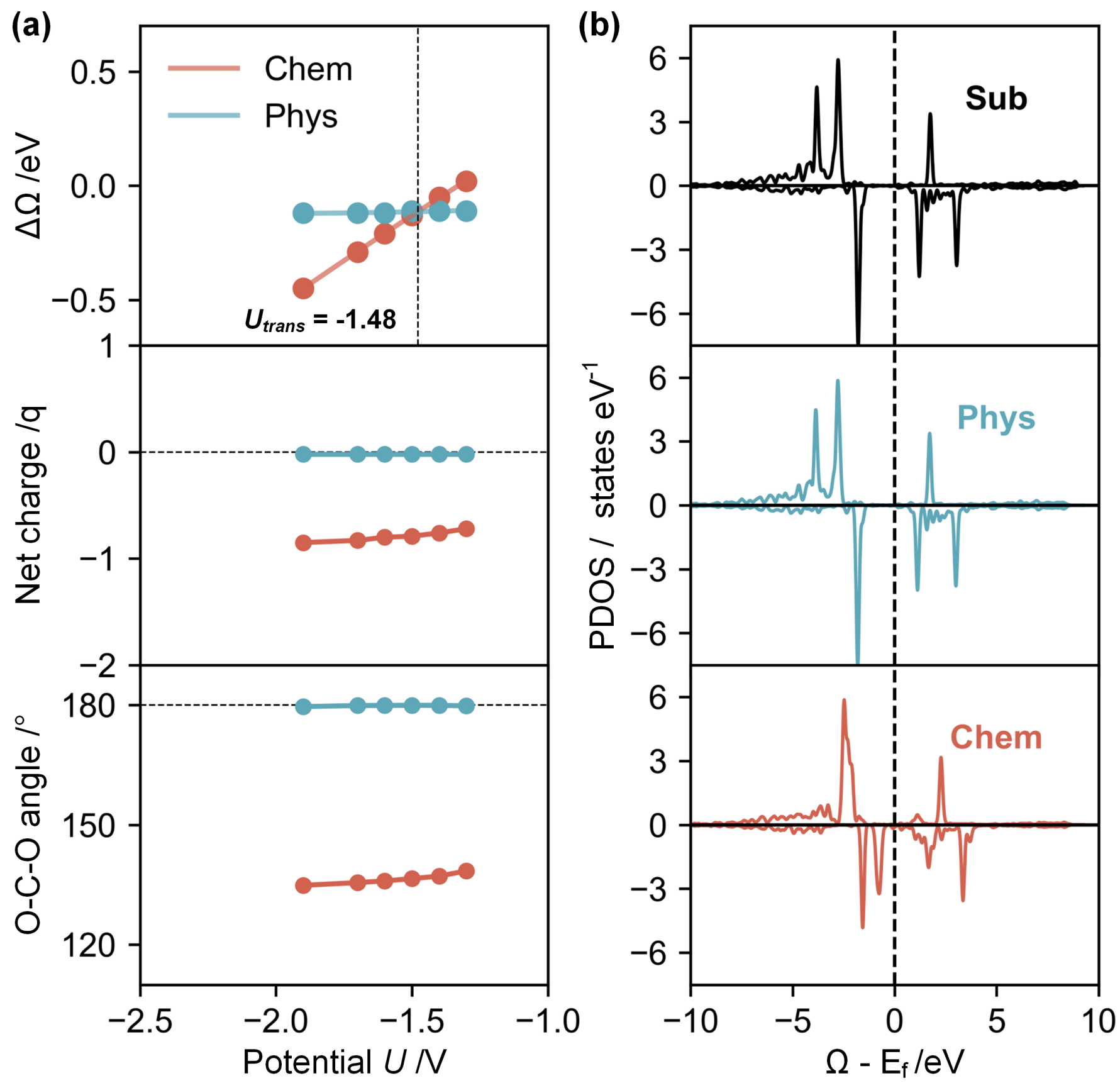

Figure S19. (a) Adsorption energy diagram, net charge transfer, and CO_2 angle variation for the minimum position on the physisorption and chemisorption curves on Fe-N_4 SAC as a function of applied potential *U*. For the Fe-N_4 SAC, CO_2 chemisorption only presents the stable η^1-C configuration, while the other three modes transition to the η^1-C or physisorption after geometry optimization. (b) Partial density of states (PDOS) of the Fe active center before and after the CO_2 physisorption/chemisorption at U_{trans}.

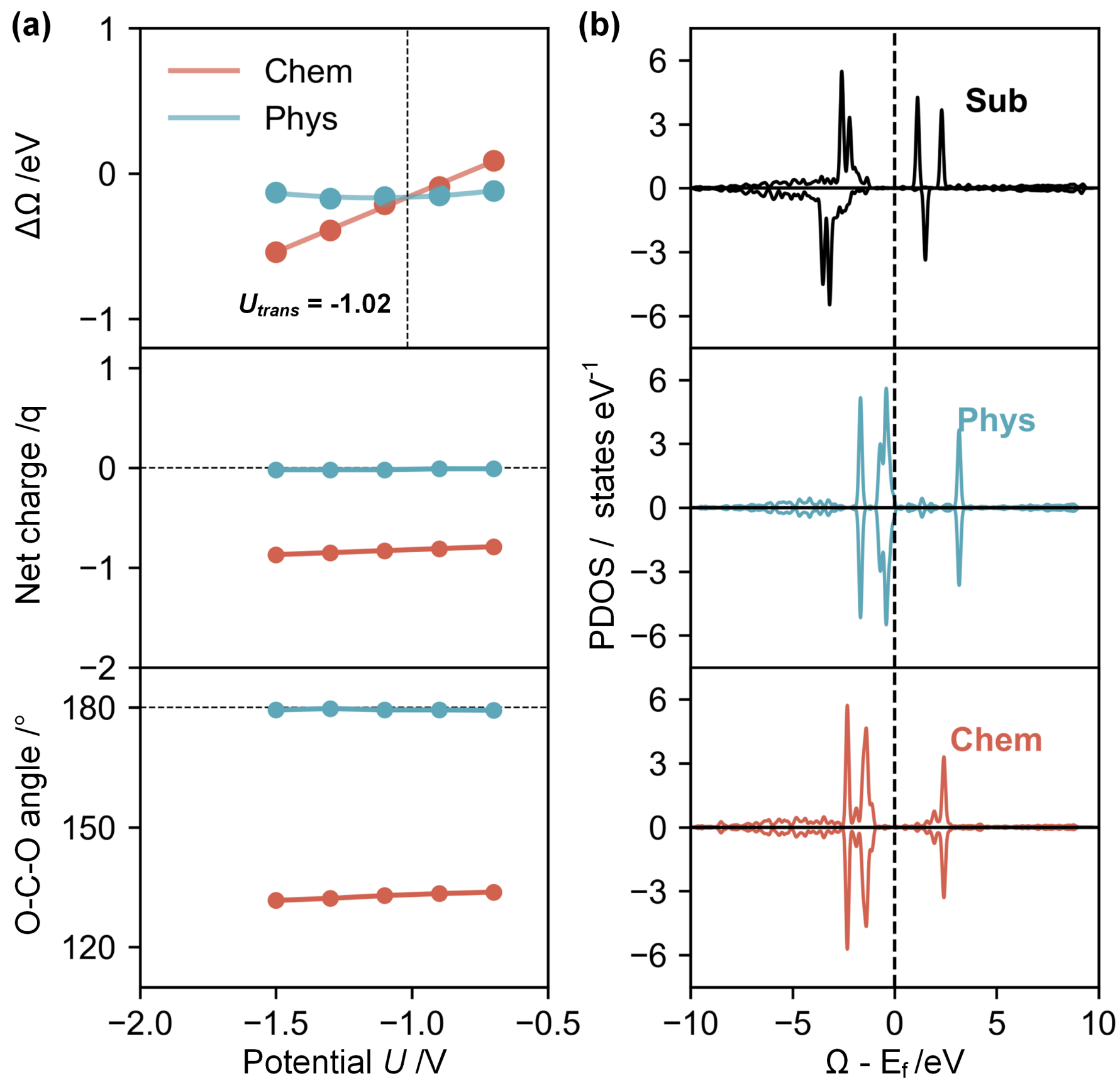

Figure S20. (a) Adsorption energy diagram, net charge transfer, and CO_2 angle variation for the minimum position on the physisorption and chemisorption curves on Co-N_4 SAC as a function of applied potential *U*. For the Co-N_4 SAC, CO_2 chemisorption only presents the stable η^1-C configuration, while the other three modes transition to the η^1-C or physisorption after geometry optimization. (b) Partial density of states (PDOS) of the Co active center before and after the CO_2 physisorption/chemisorption at U_{trans}.

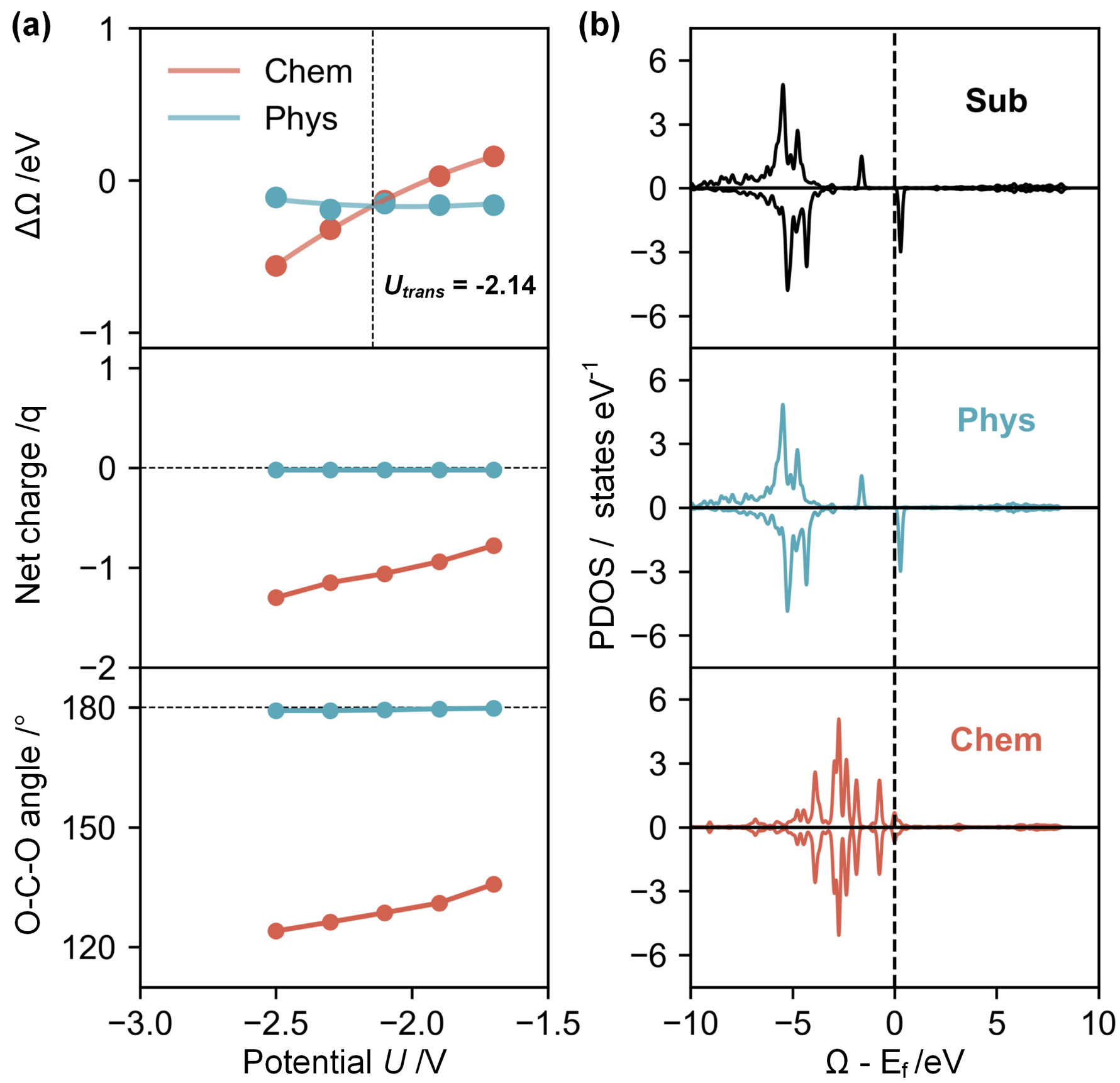

Figure S21. (a) Adsorption energy diagram, net charge transfer, and CO_2 angle variation for the minimum position on the physisorption and chemisorption curves on Cu-N_4 SAC as a function of applied potential U. For the Cu-N_4 SAC, CO_2 chemisorption only presents the stable η^1-C configuration, while the other three modes transition to the η^1-C or physisorption after geometry optimization. (b) Partial density of states (PDOS) of the Cu active center before and after the CO_2 physisorption/chemisorption at U_{trans}.

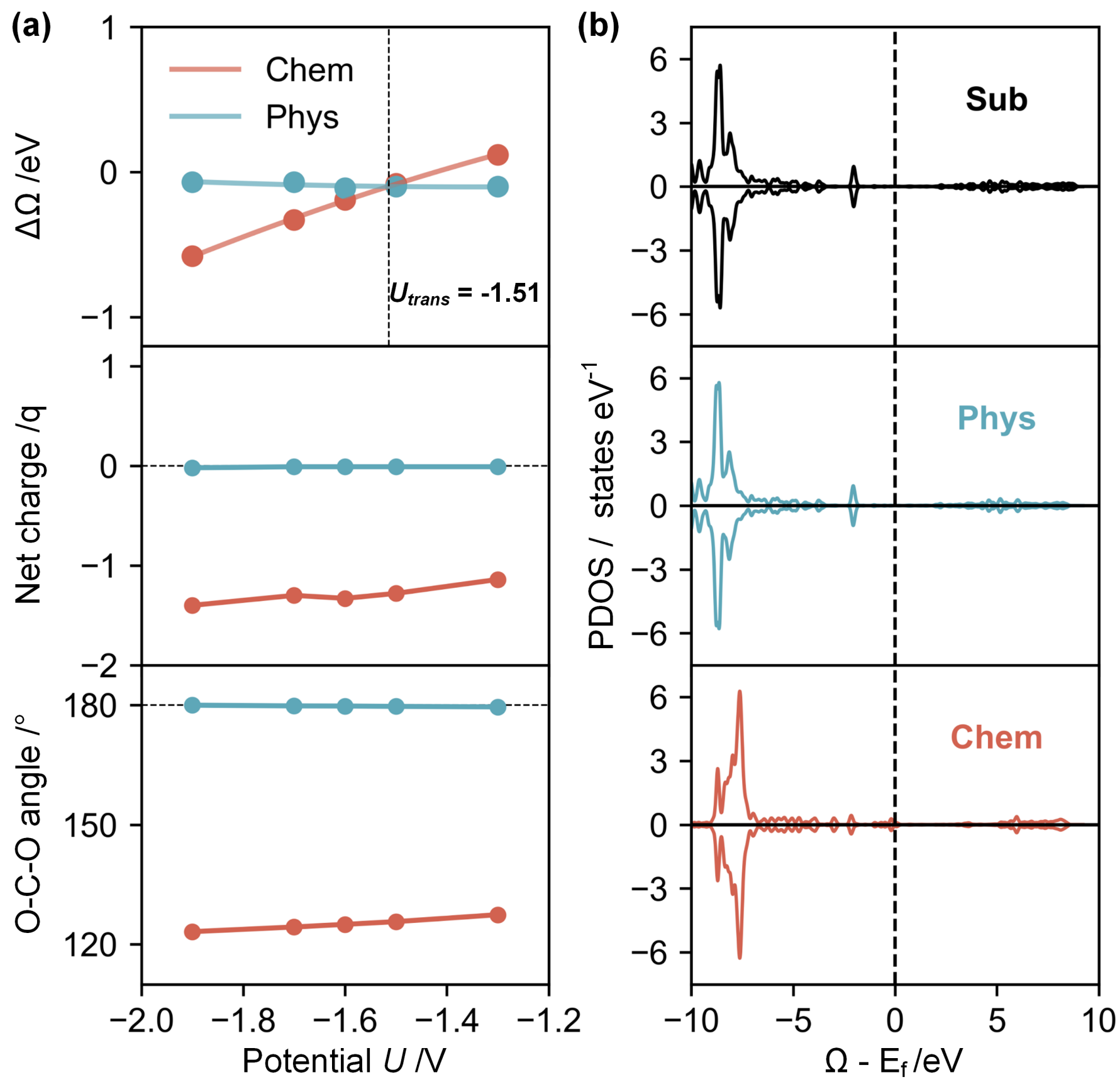

Figure S22. (a) Adsorption energy diagram, net charge transfer, and CO_2 angle variation for the minimum position on the physisorption and chemisorption curves on Zn-N_4 SAC as a function of applied potential *U*. For the Zn-N_4 SAC, CO_2 chemisorption only presents the η^1-C configuration, while the other three modes transition to the η^1-C or physisorption after geometry optimization. (b) Partial density of states (PDOS) of the Zn active center before and after the CO_2 physisorption/chemisorption at U_{trans}.

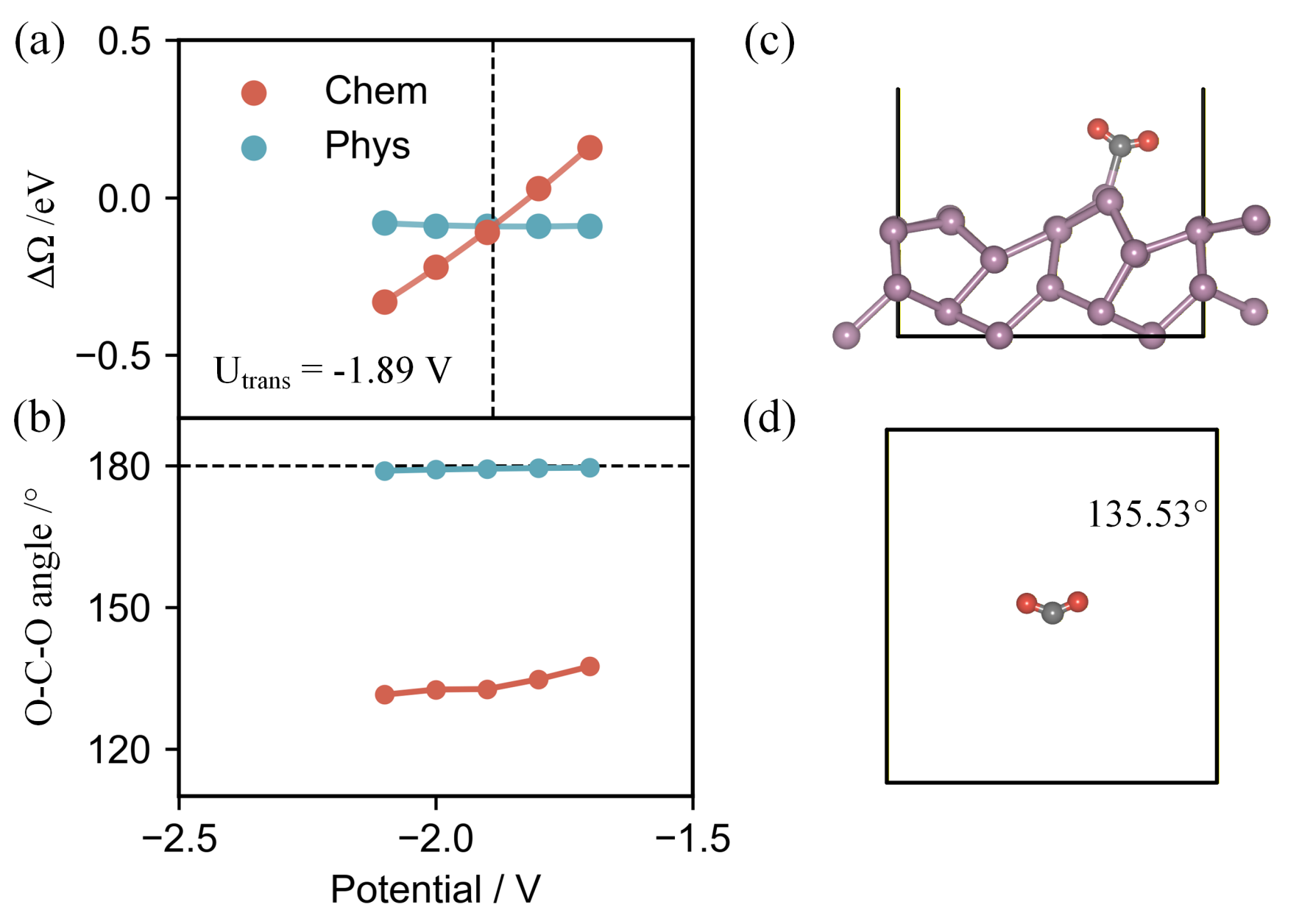

Figure S23. (a) Potential-dependent Landau adsorption free energies (ΔΩ) of the CO_2 physisorption (blue) and chemisorption (red) on Hg-(100) facet. (b) O–C–O bond angle for the minimum-energy states along the physisorption and chemisorption curves. (c) Optimized structure of CO_2 chemisorbed on Hg-(100) facet, shown as a representative configuration at -1.9 V. (d) Optimized structure of CO_2 with one additional electron injected in the solvation environment. The C, O, and Hg atoms are shown in grey, red, and purple, respectively.

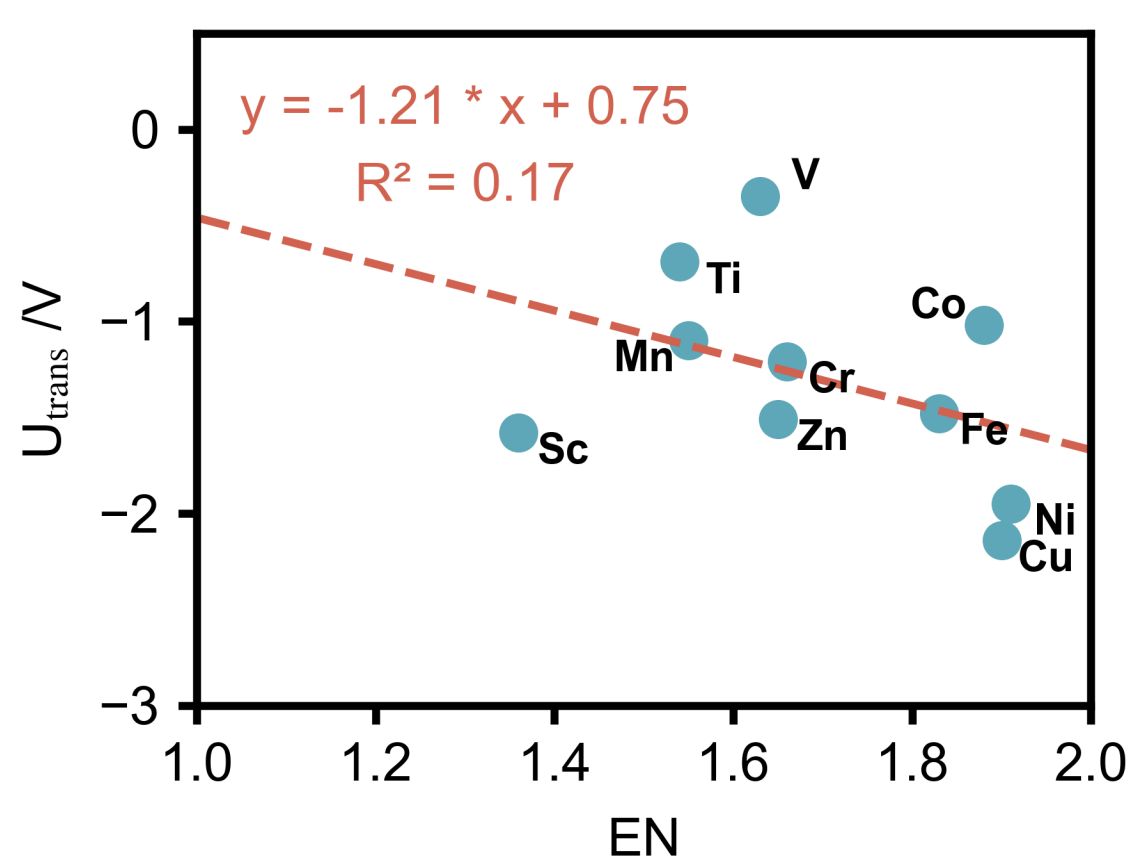

Figure S24. Linear relationship between U_{trans} and electronegativity.

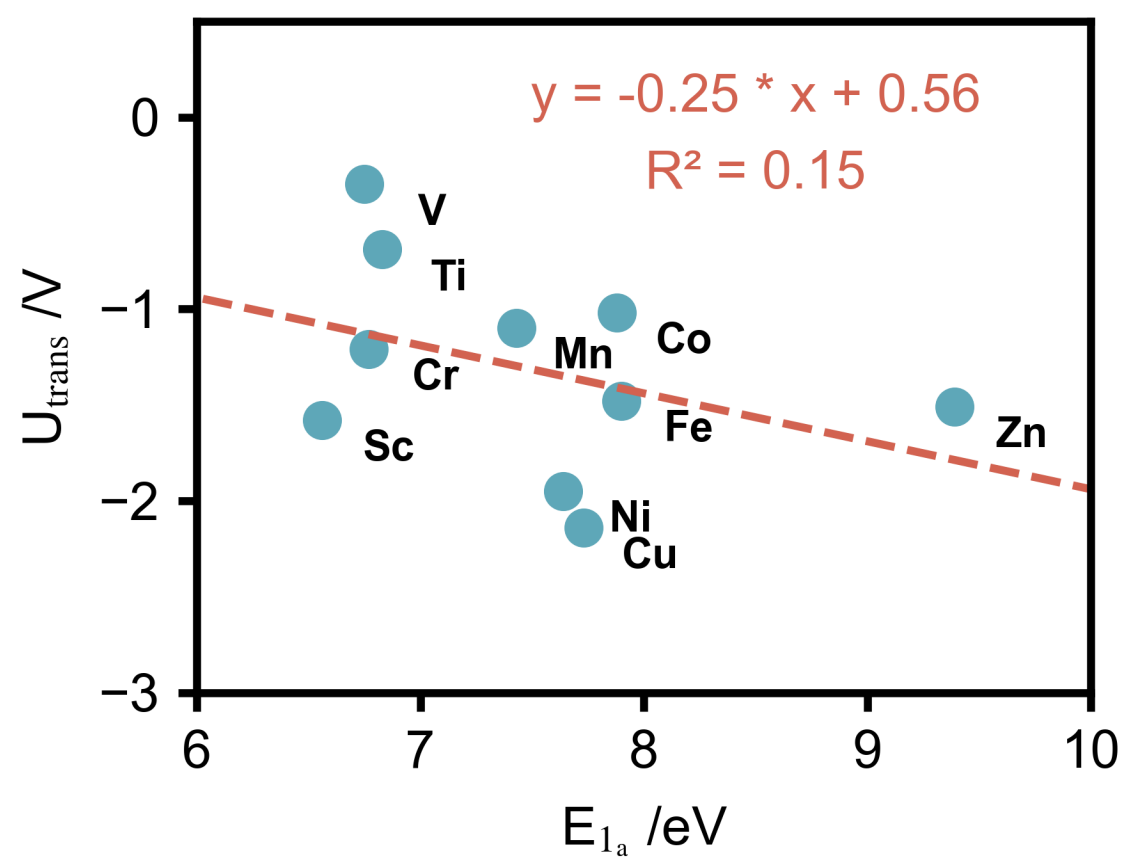

Figure S25. Linear relationship between U_{trans} and the first ionization energy.

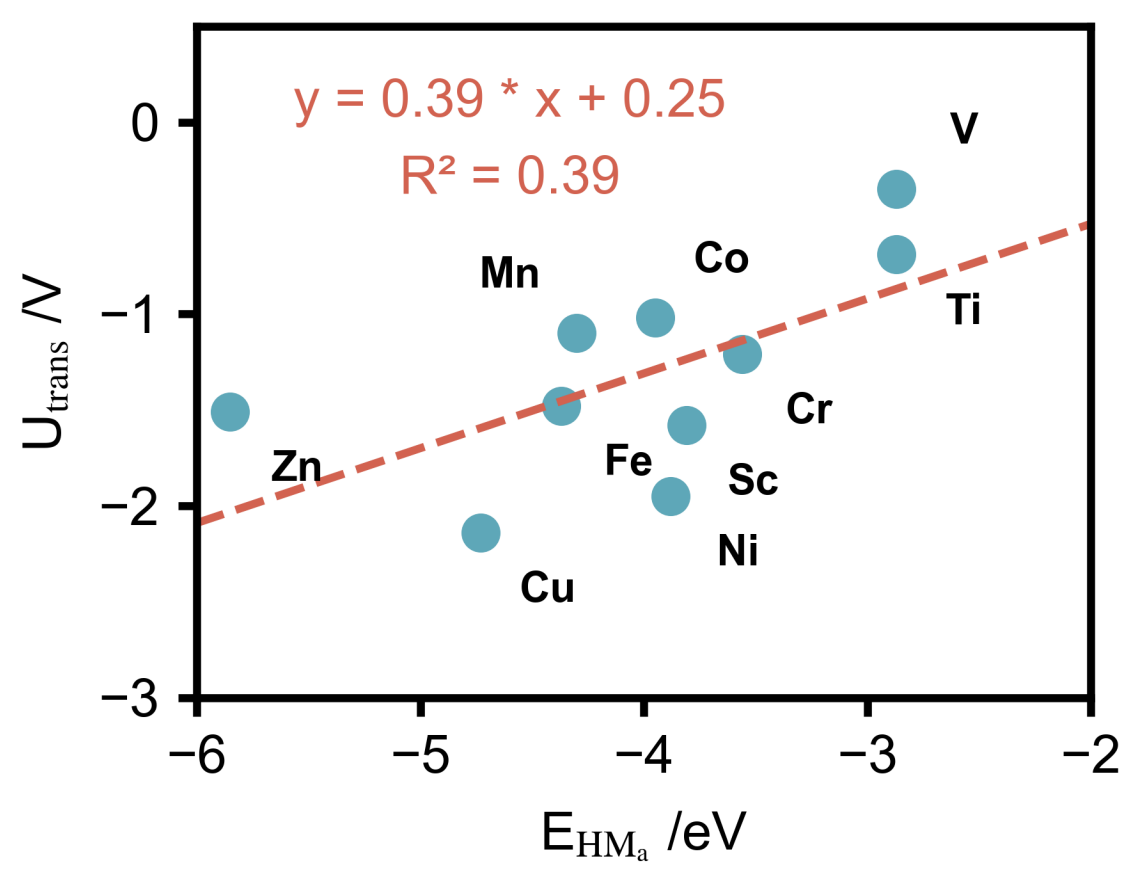

Figure S26. Linear relationship between U_{trans} and the highest occupied molecular orbital.

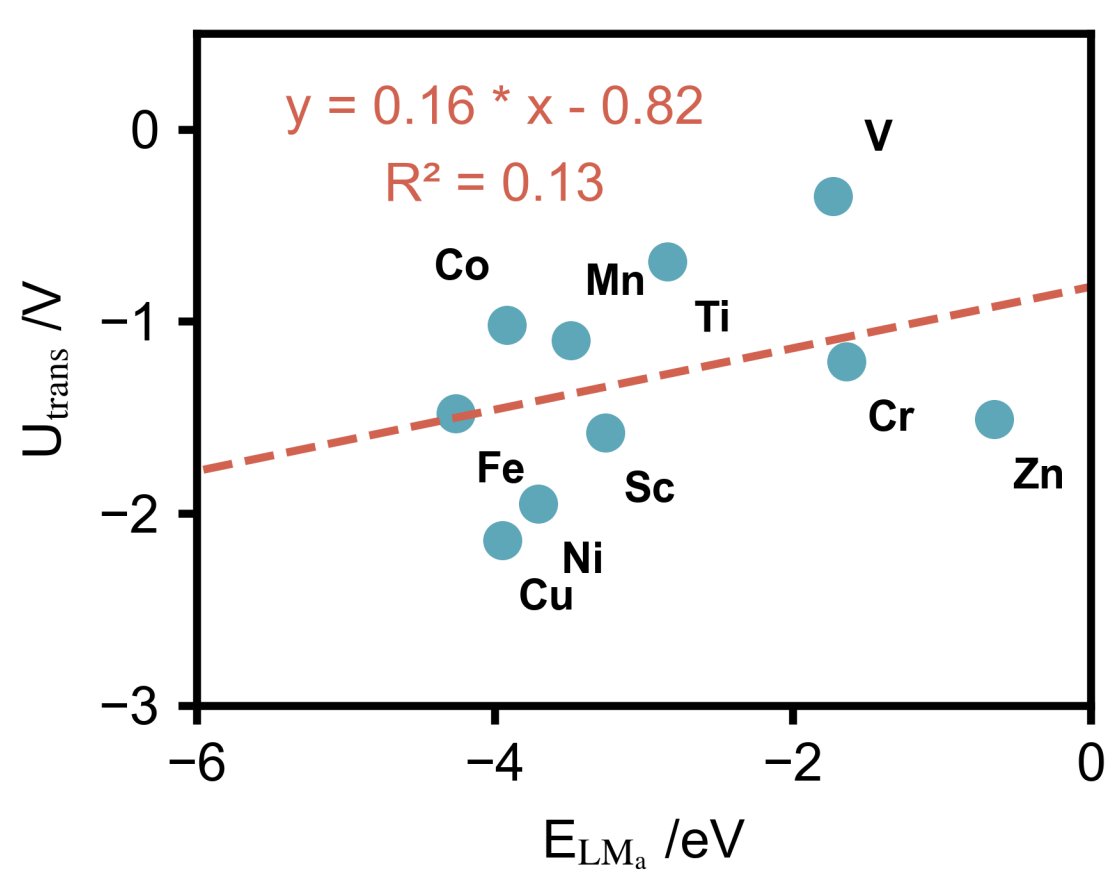

Figure S27. Linear relationship between U_{trans} and the lowest unoccupied molecular orbital.

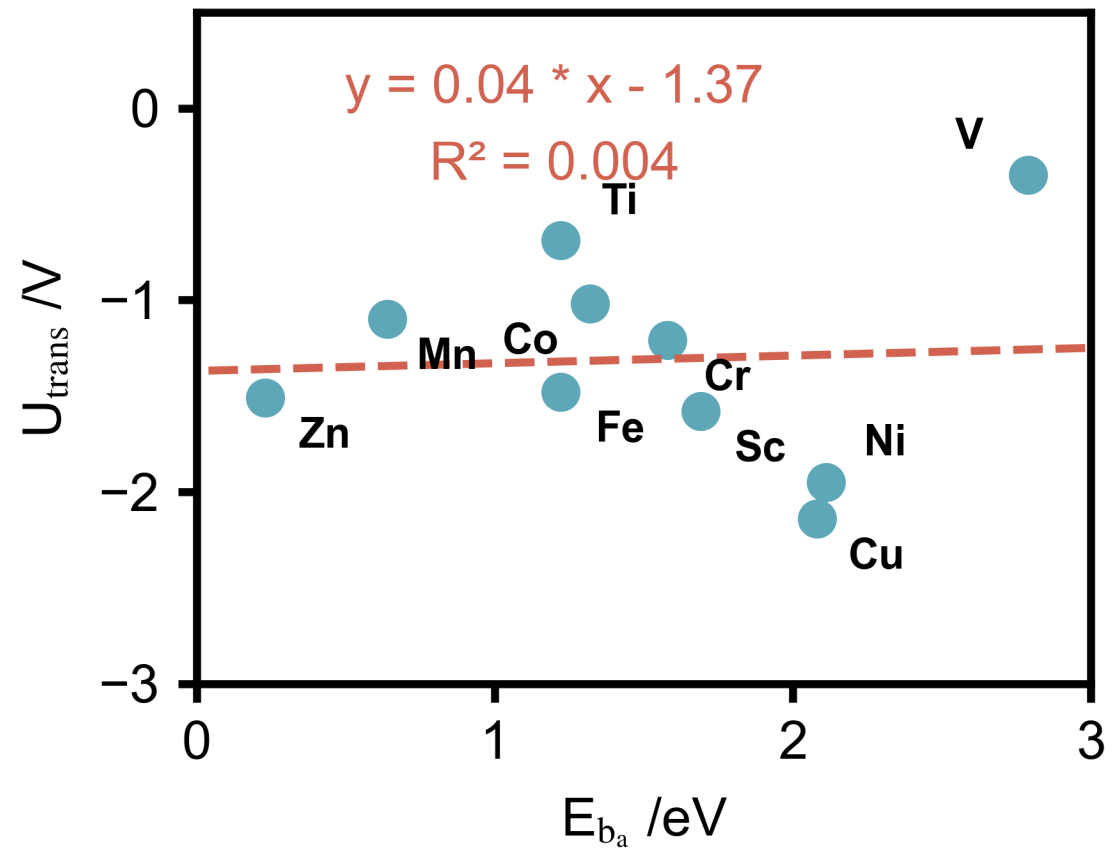

Figure S28. Linear relationship between U_{trans} and binding energy of metal dimer.

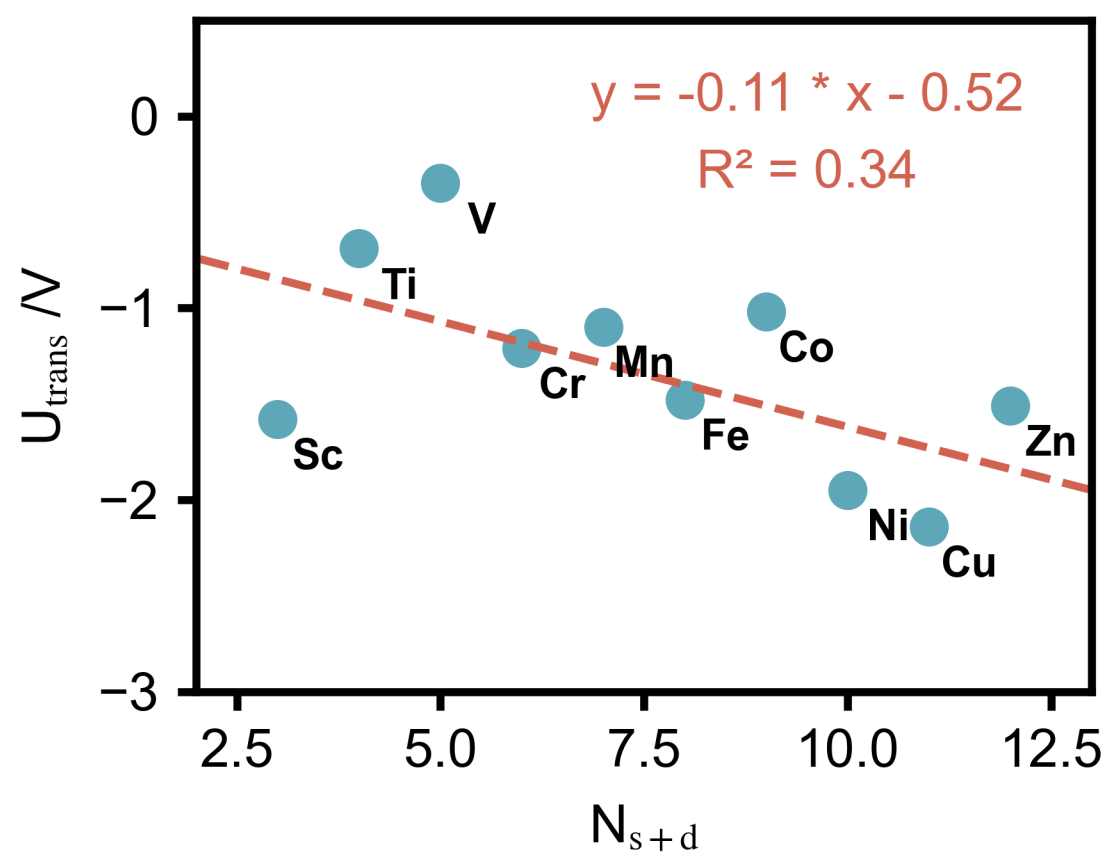

Figure S29. Linear relationship between U_{trans} and the electron numbers in d and s orbitals.

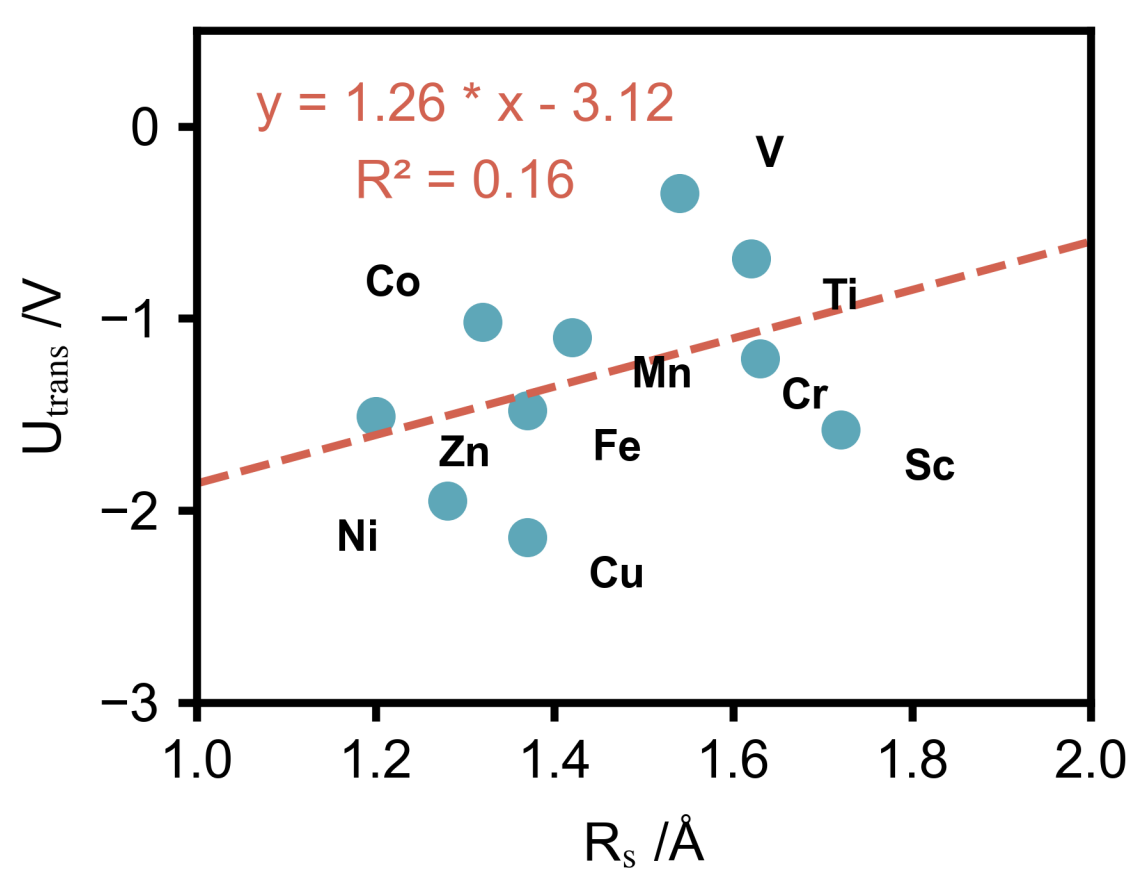

Figure S30. Linear relationship between U_{trans} and s orbital radius.

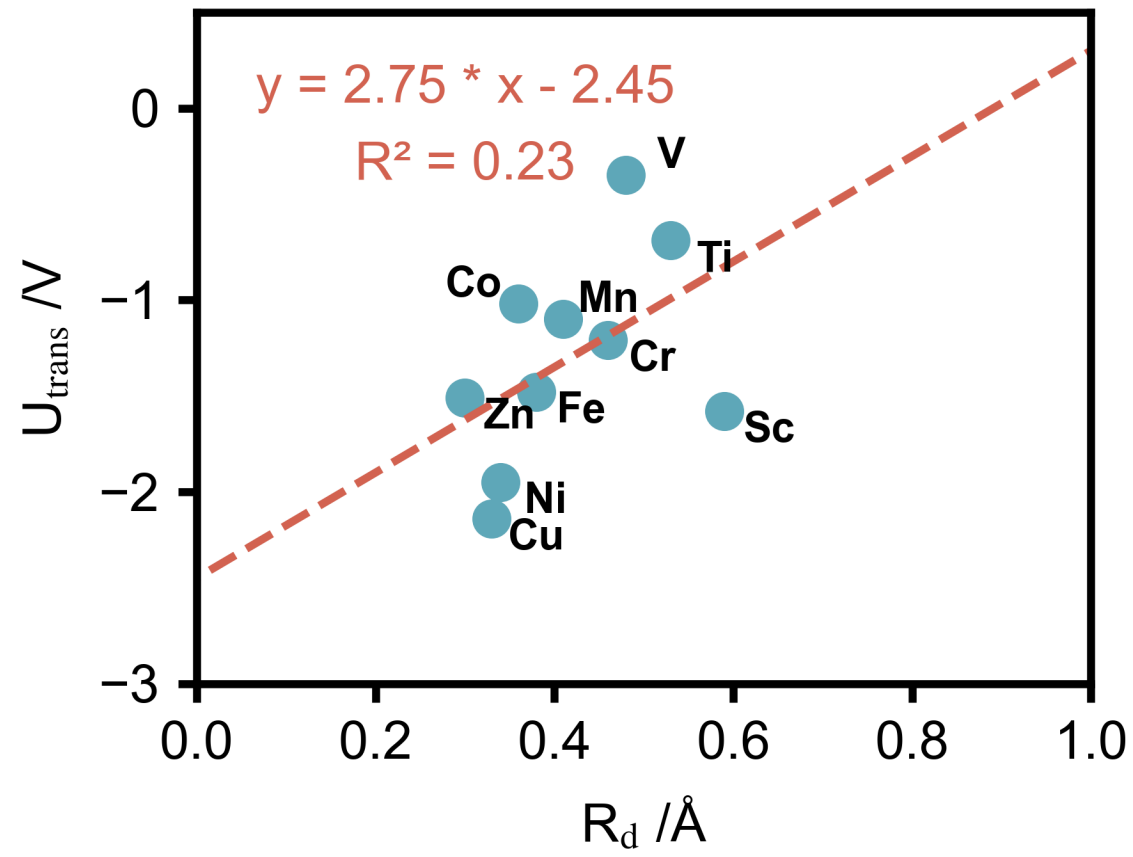

Figure S31. Linear relationship between U_{trans} and d orbital radius.

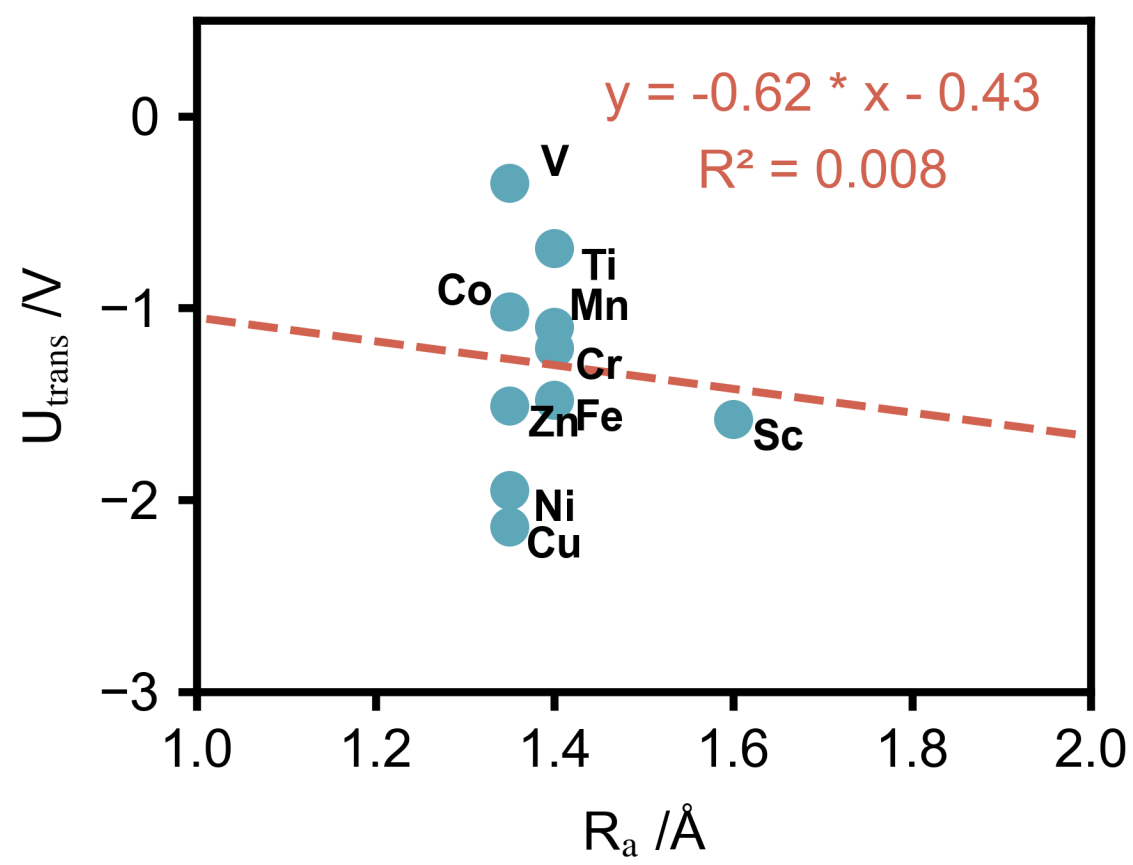

Figure S32. Linear relationship between U_{trans} and atomic radius.

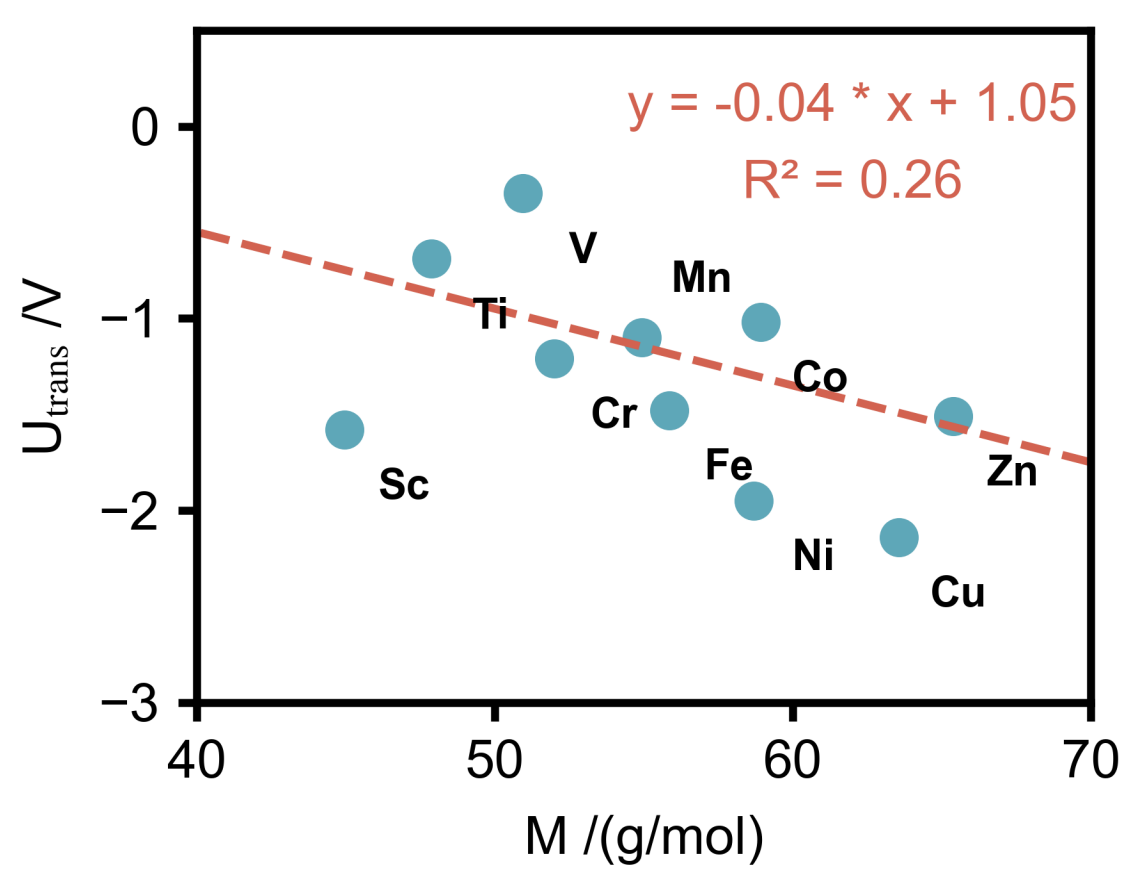

Figure S33. Linear relationship between U_{trans} and mass.

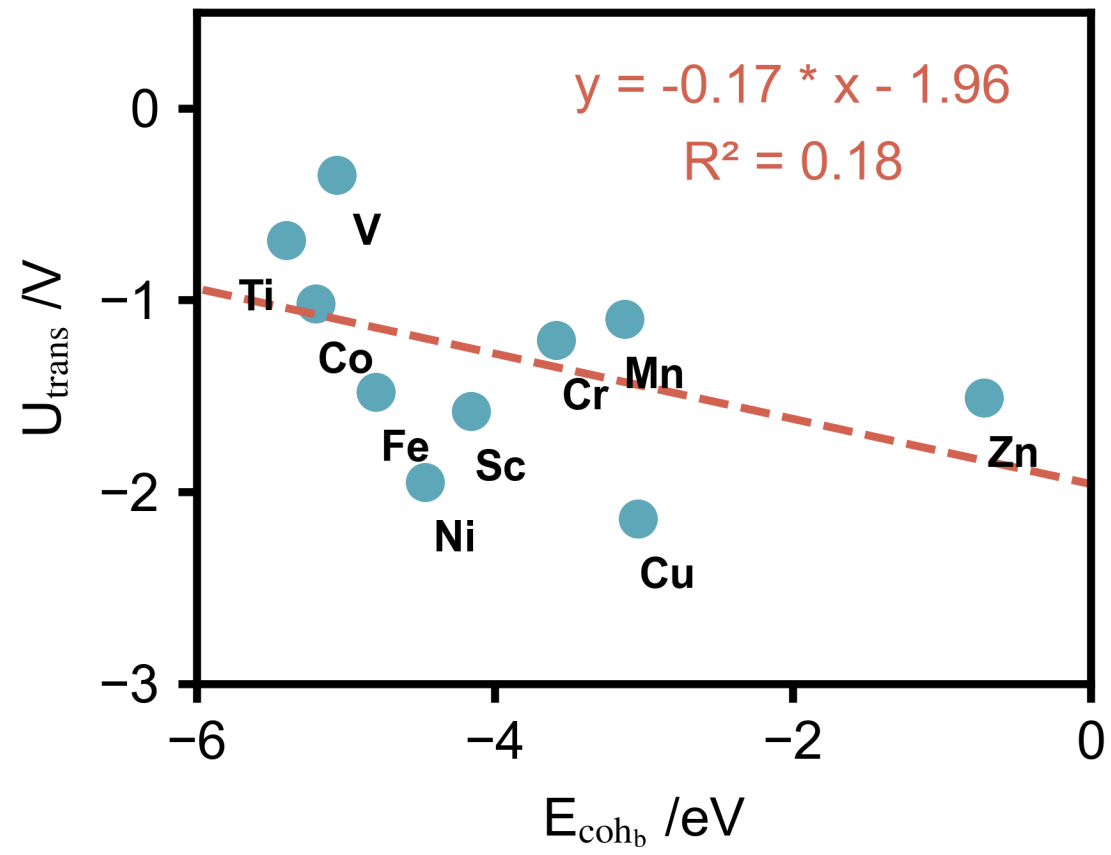

Figure S34. Linear relationship between U_{trans} and cohesive energy.

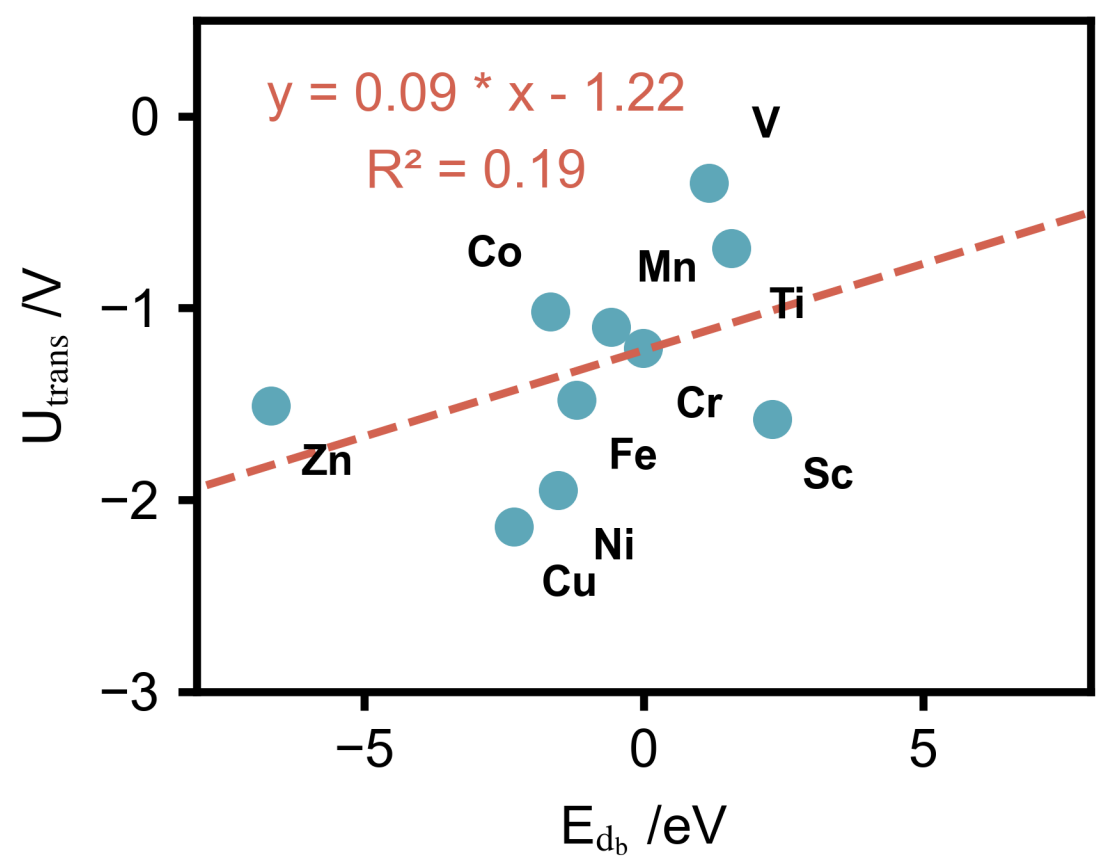

Figure S35. Linear relationship between U_{trans} and the energy of d-band center.

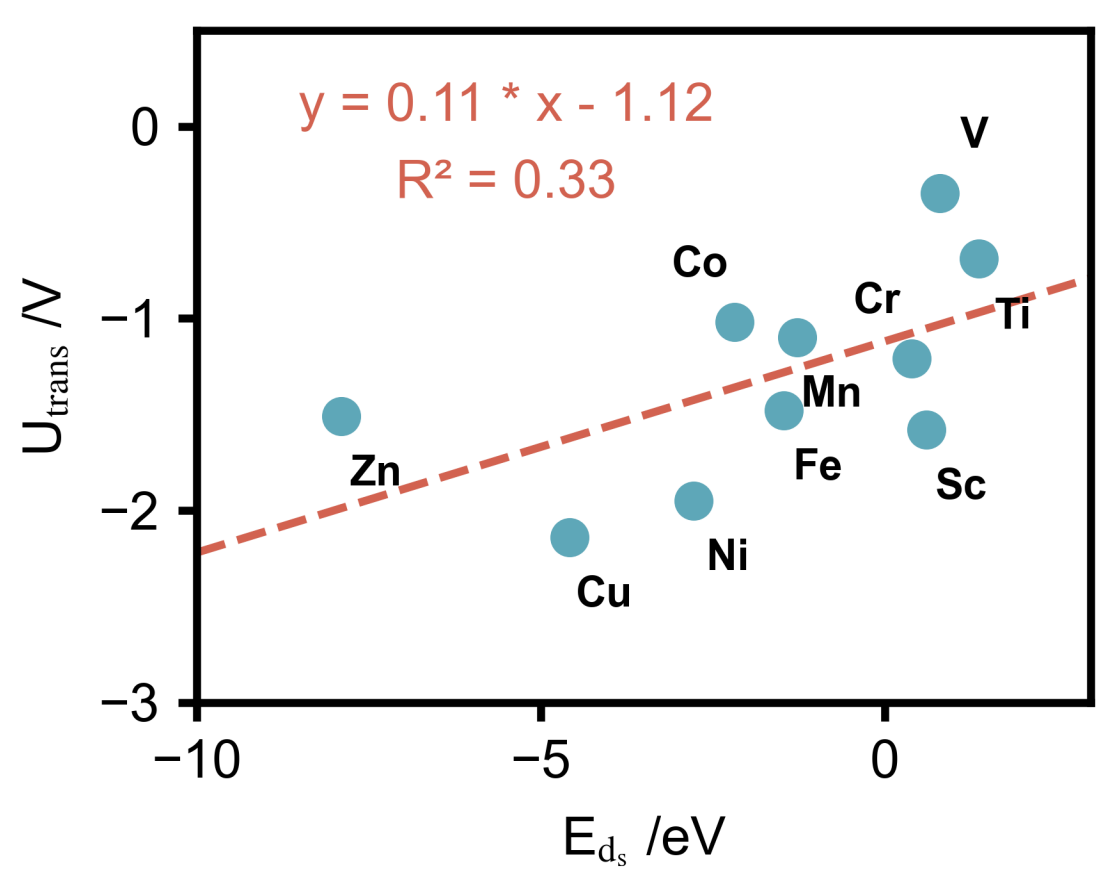

Figure S36. Linear relationship between U_{trans} and the energy of d-band center on SACs.

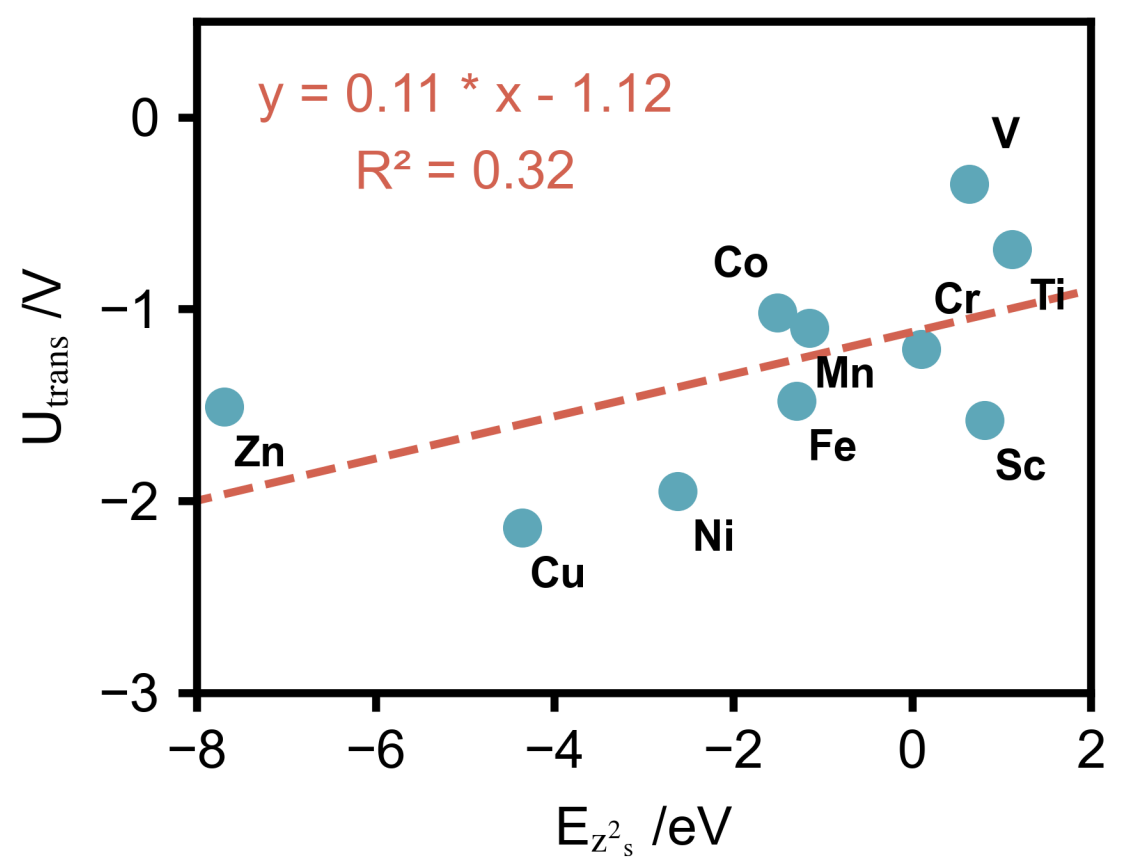

Figure S37. Linear relationship between U_{trans} and the energy of d_{z^2}-band center on SACs.

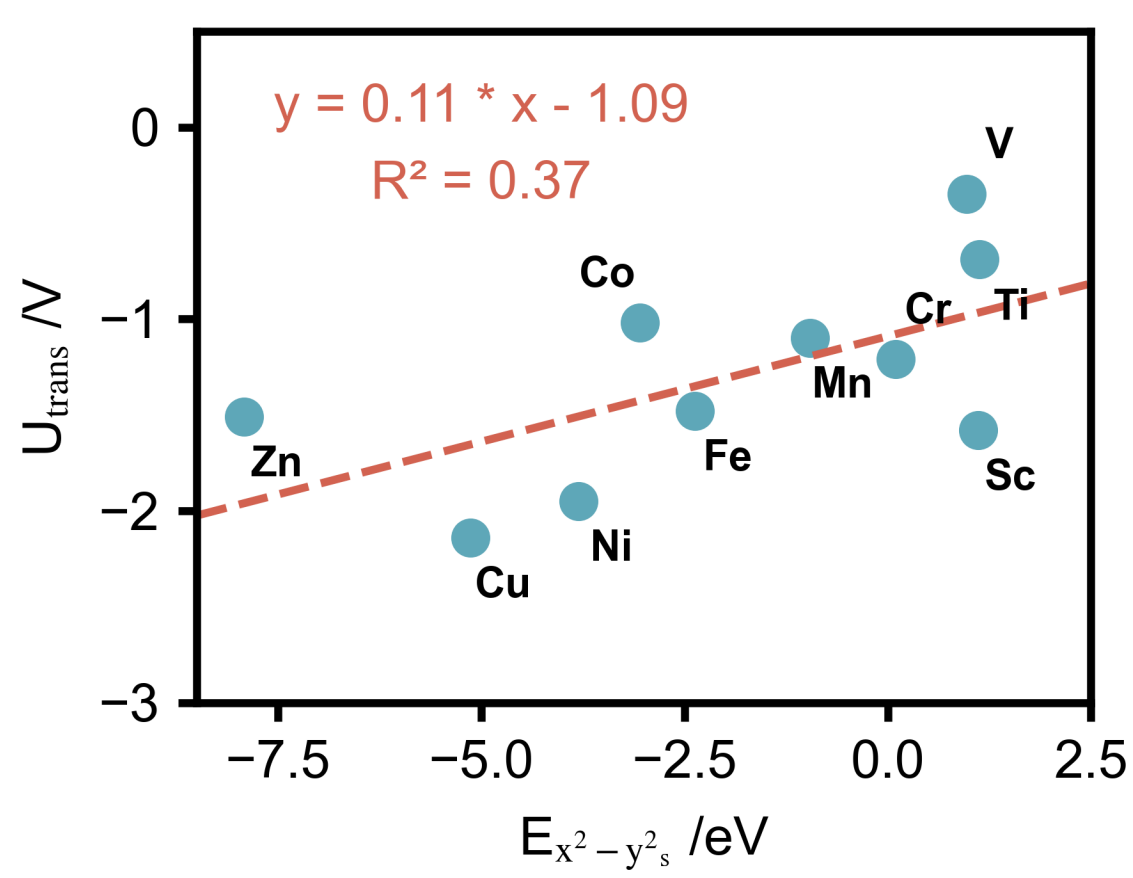

Figure S38. Linear relationship between U_{trans} and the energy of $d_{x^2-y^2}$-band center on SACs.

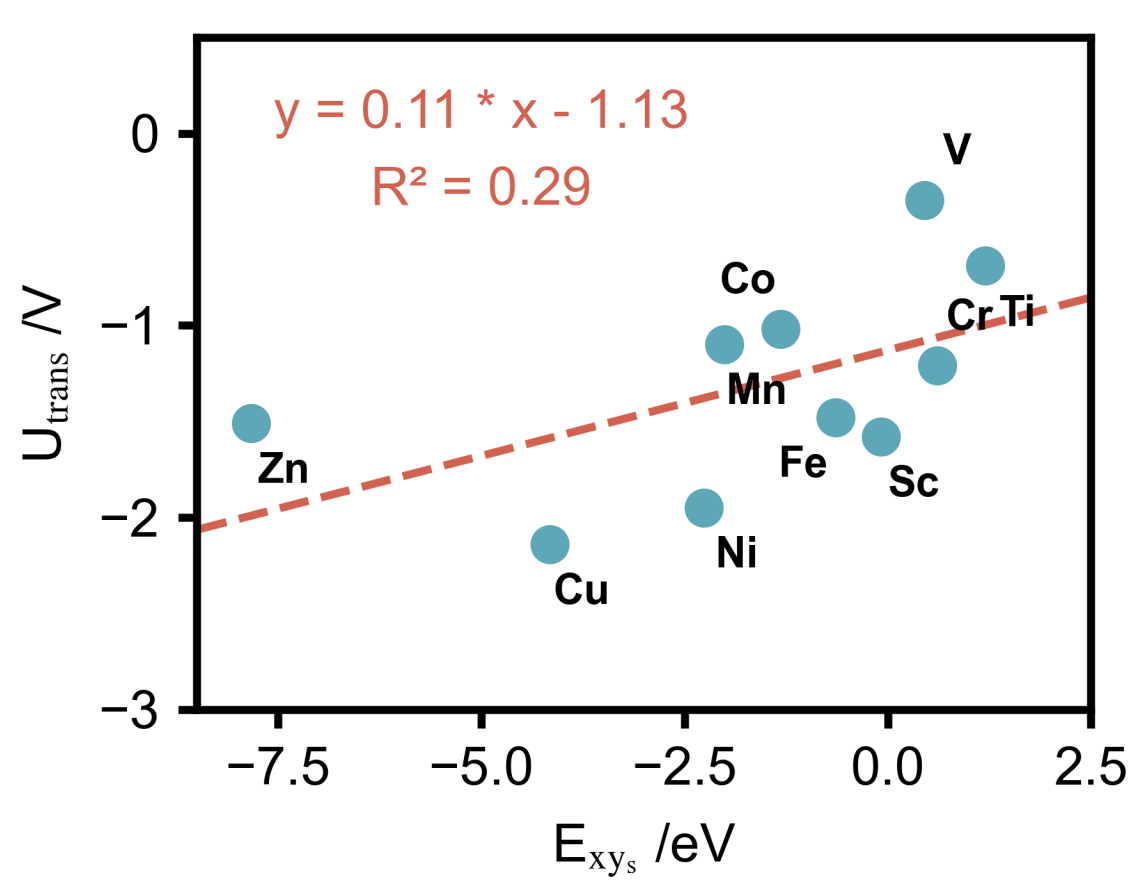

Figure S39. Linear relationship between U_{trans} and the energy of d_{xy}-band center on SACs.

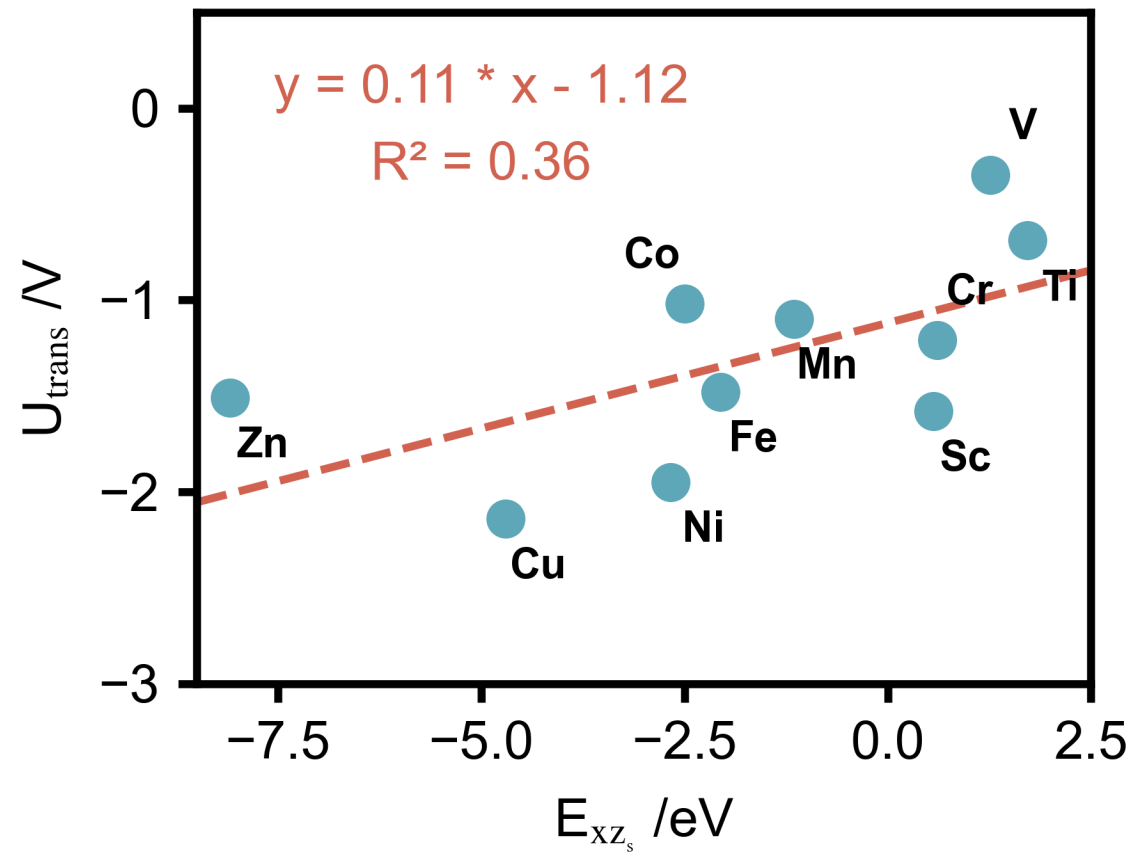

Figure S40. Linear relationship between U_{trans} and the energy of d_{xz}-band center on SACs.

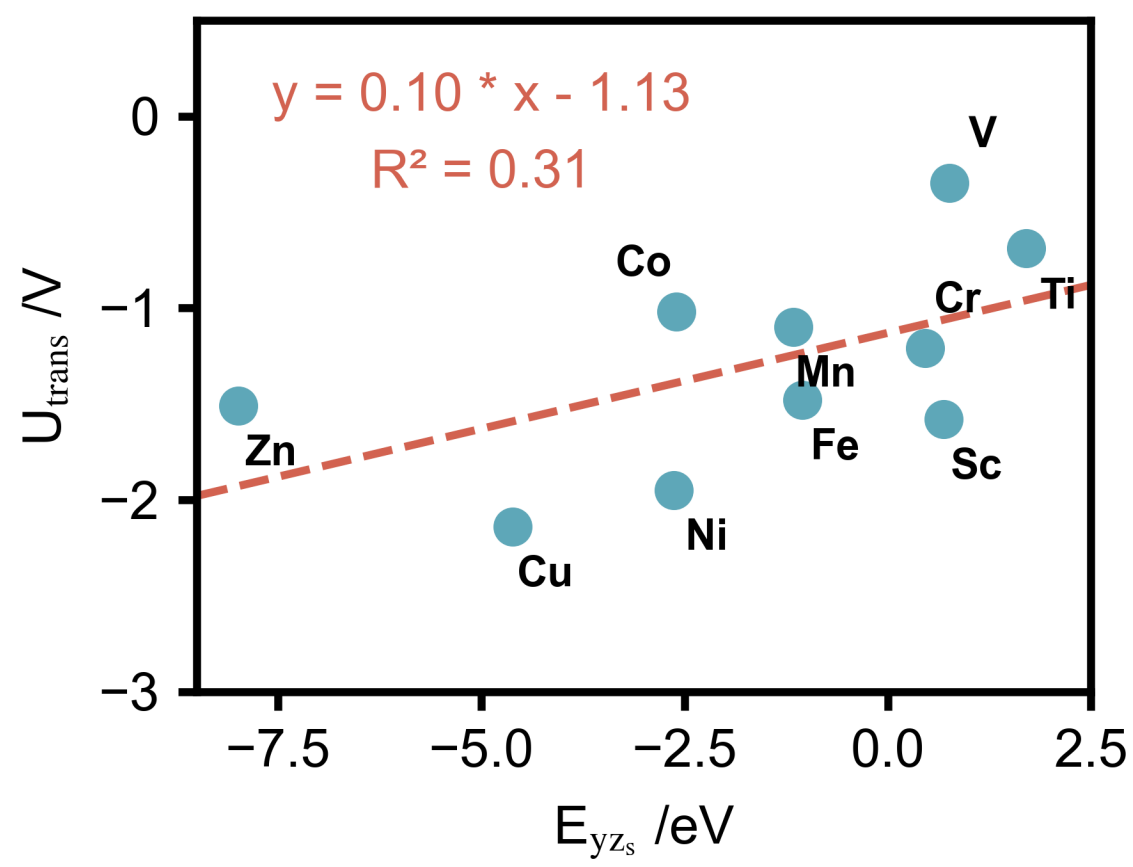

Figure S41. Linear relationship between U_{trans} and the energy of d_{yz}-band center on SACs.

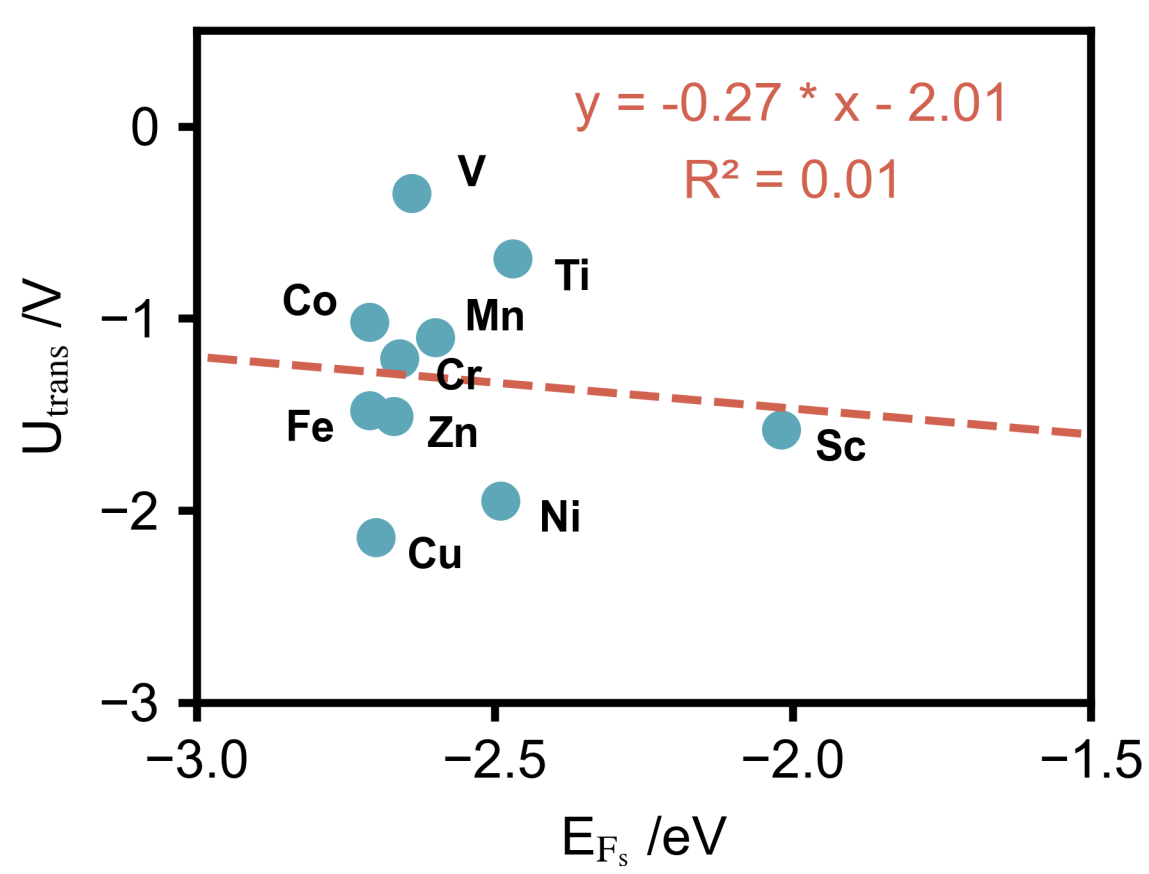

Figure S42. Linear relationship between U_{trans} and the Fermi energy on SACs.

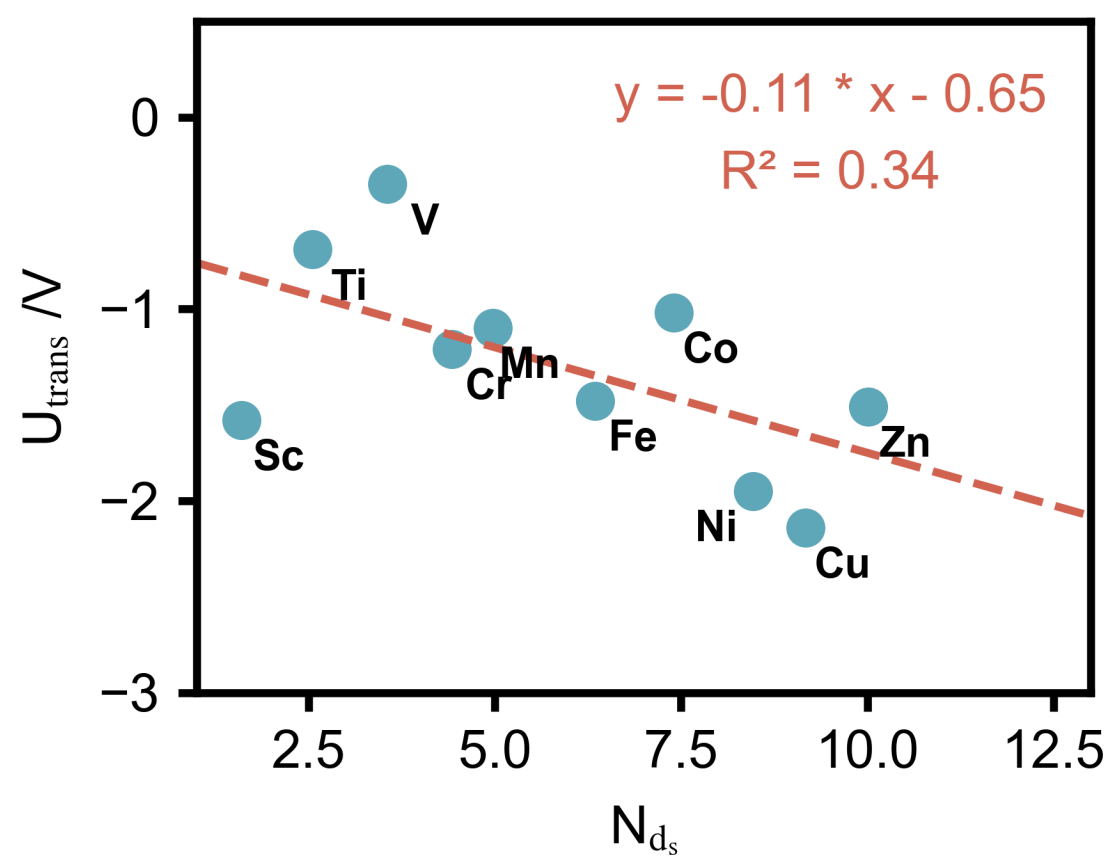

Figure S43. Linear relationship between U_{trans} and the electron number in d orbitals in grand state on SACs.

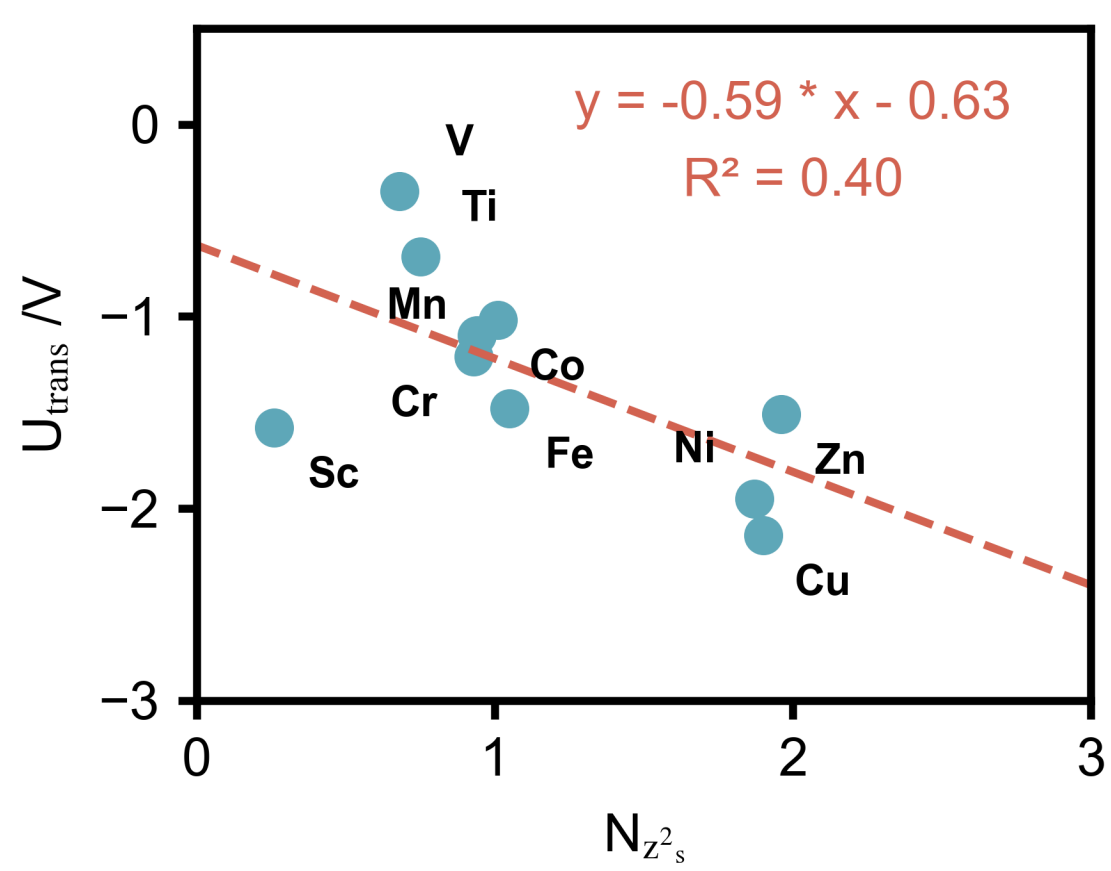

Figure S44. Linear relationship between U_{trans} and the electron number in d_{z^2} orbitals in grand state on SACs.

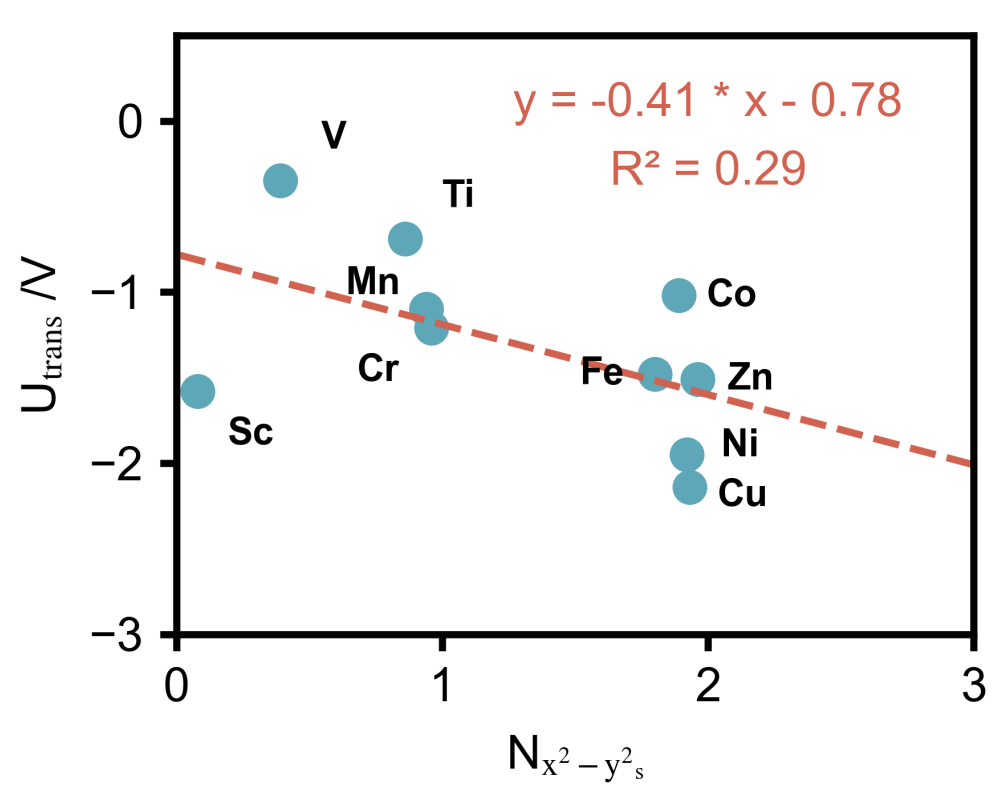

Figure S45. Linear relationship between U_{trans} and the electron number in $d_{x^2-y^2}$ orbitals in the grand state on SACs.

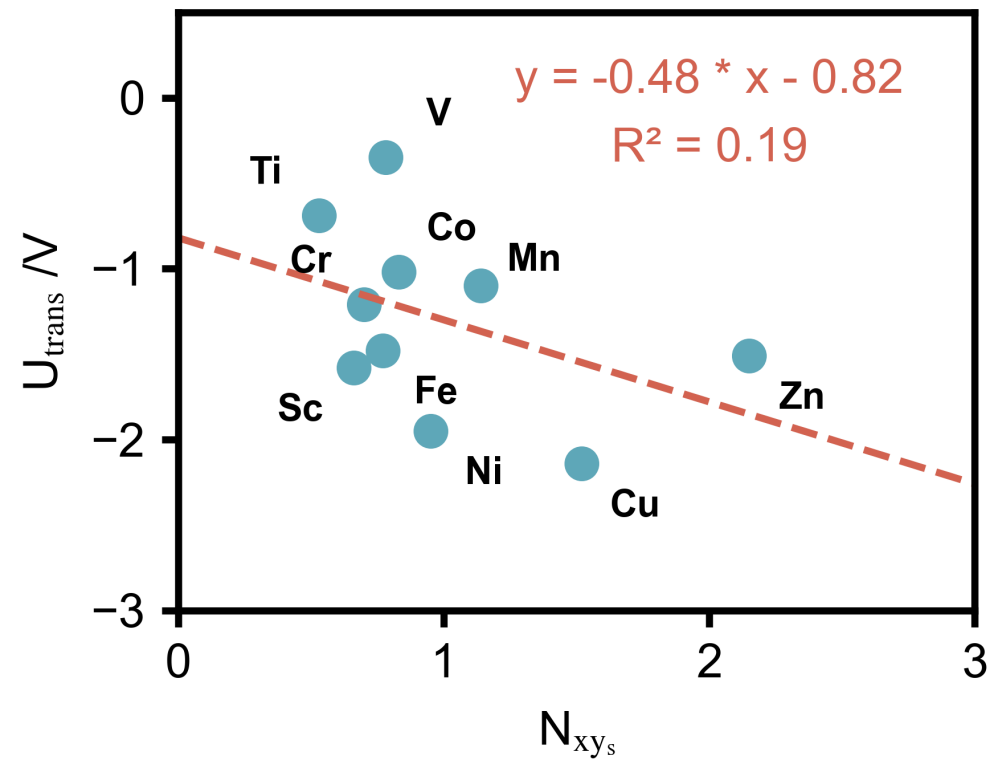

Figure S46. Linear relationship between U_{trans} and the electron number in d_{xy} orbitals in the grand state on SACs.

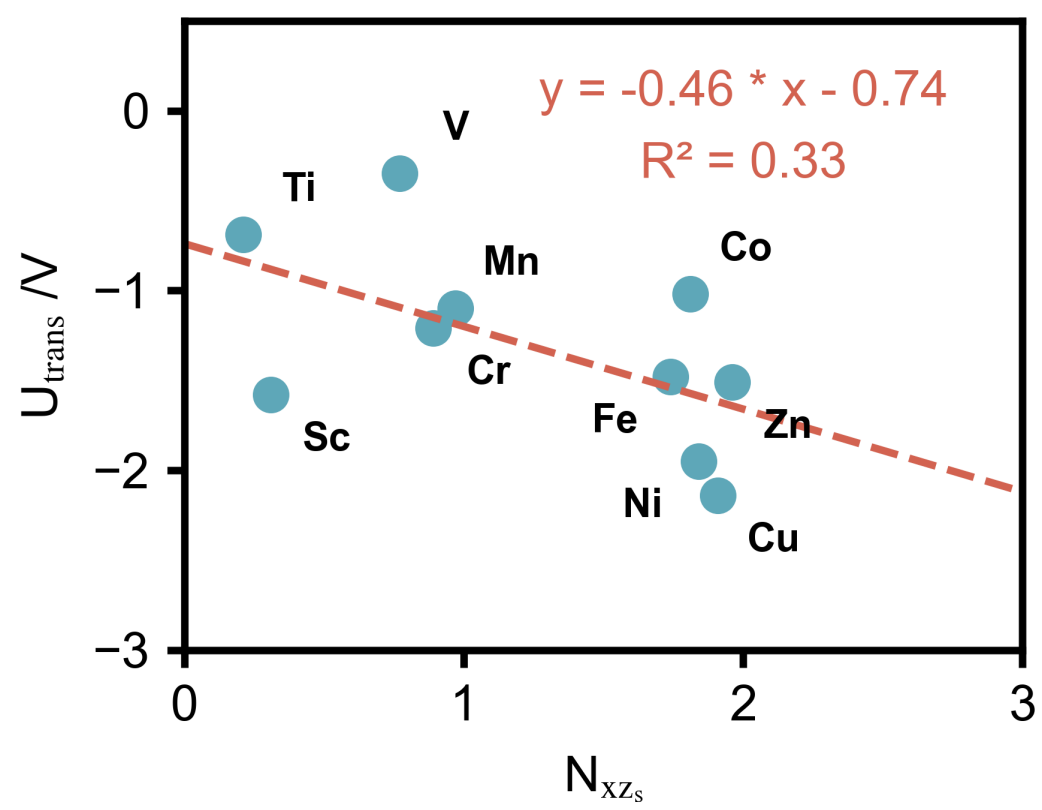

Figure S47. Linear relationship between U_{trans} and the electron number in d_{xz} orbitals in the grand state on SACs.

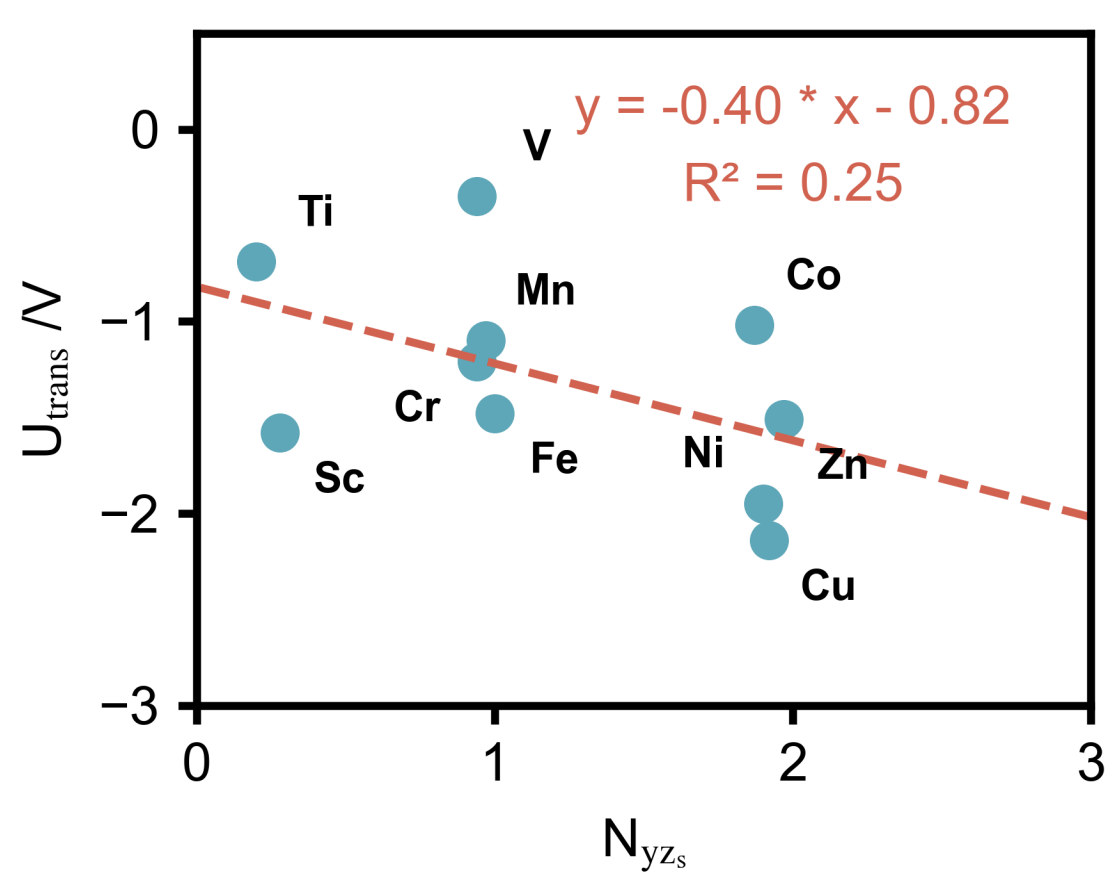

Figure S48. Linear relationship between U_{trans} and the electron number in d_{yz} orbitals in the grand state on SACs.

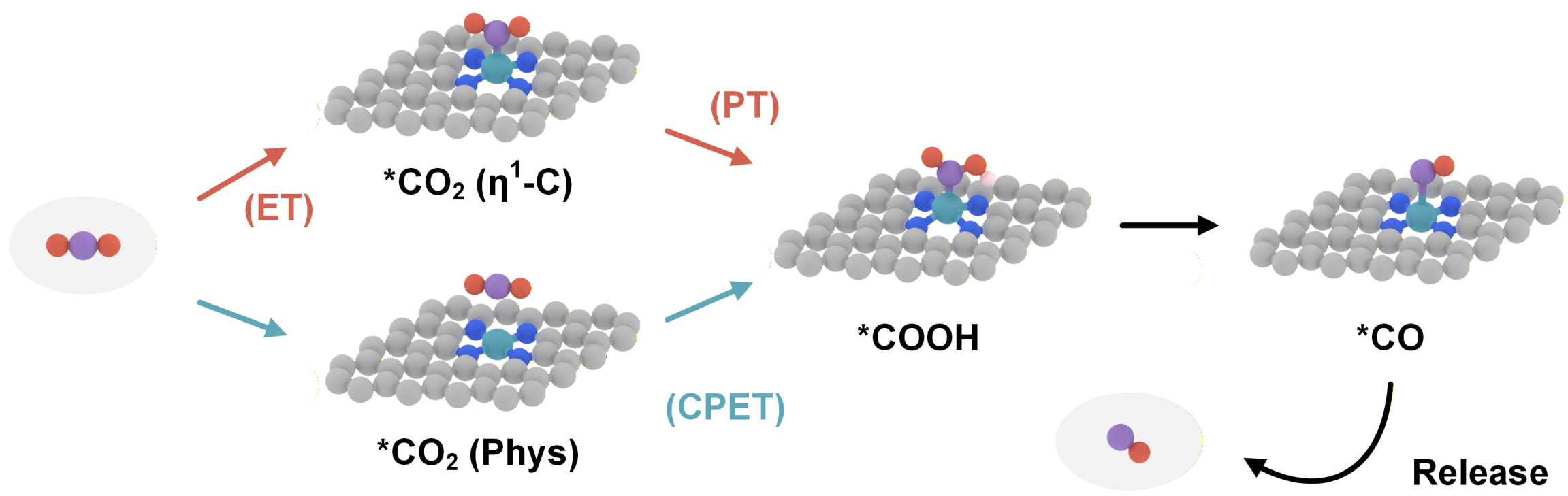

Figure S49. Illustration of CO_2RR on Ni-N_4 SACs. The C atoms in the graphene substrate are colored gray, while those in CO_2 are rendered in purple. The N, O, H, and TM atoms are shown in blue, red, pink, and cyan, respectively.

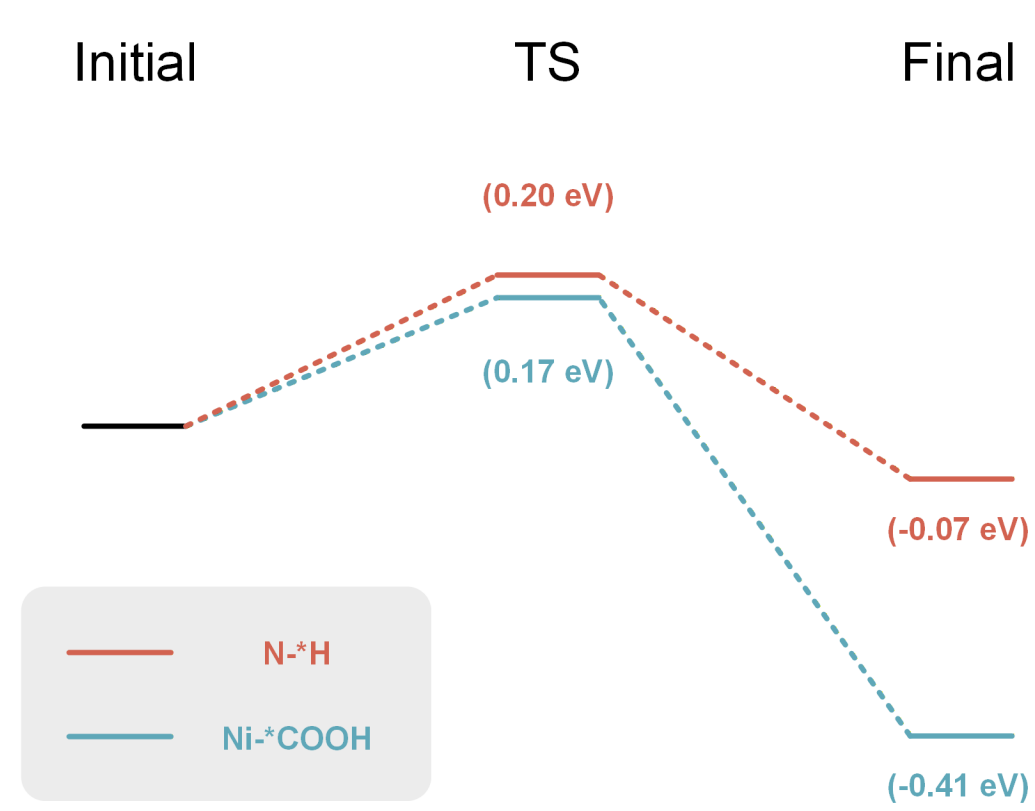

Figure S50. Reaction pathways for *COOH formation and competing *H adsorption on one of the four coordinating N sites of Ni-N_4 SAC. The computations were performed using the CI-NEB method within a commonly used hybrid solvation model,[26,27] in which 13 explicit H_2O molecules were included to represent the local solvent environment and facilitate proton transfer.

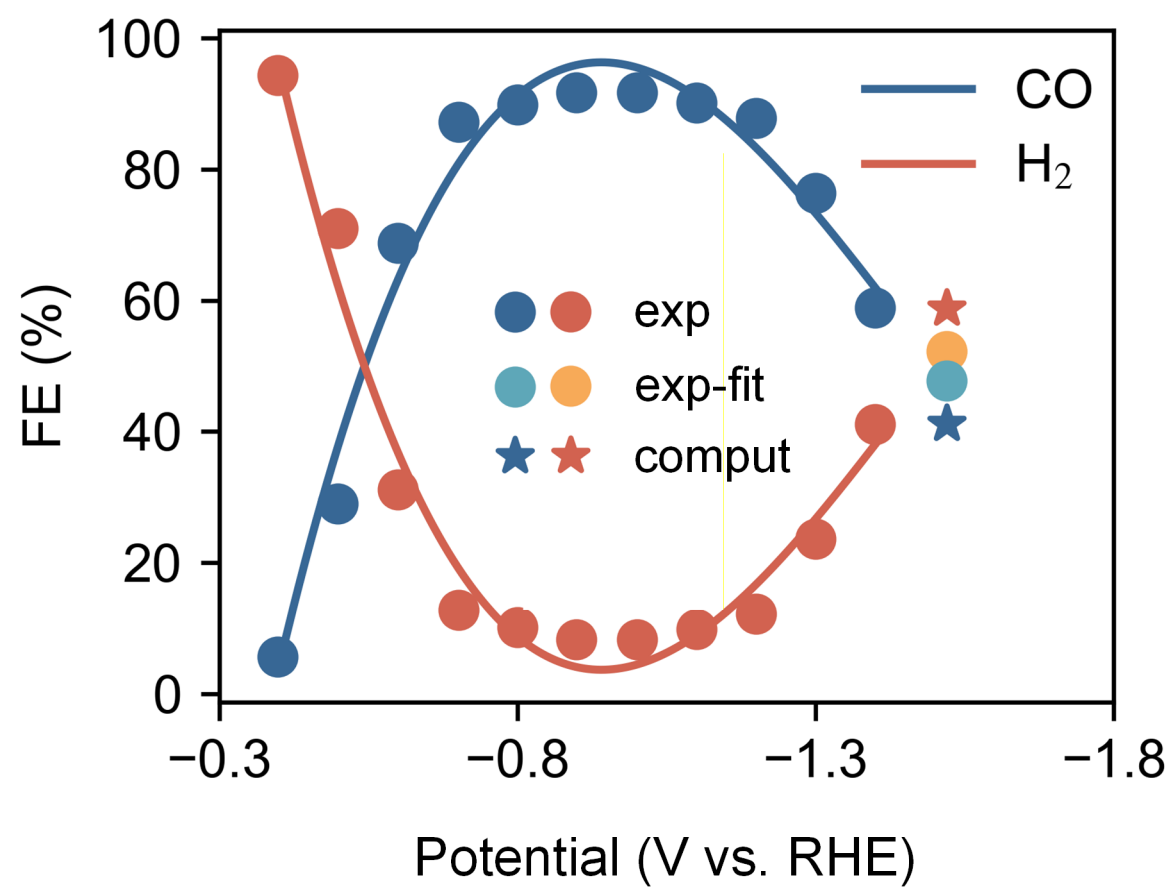

Figure S51. Comparison of Faradaic efficiencies between computation and experiment.[20]

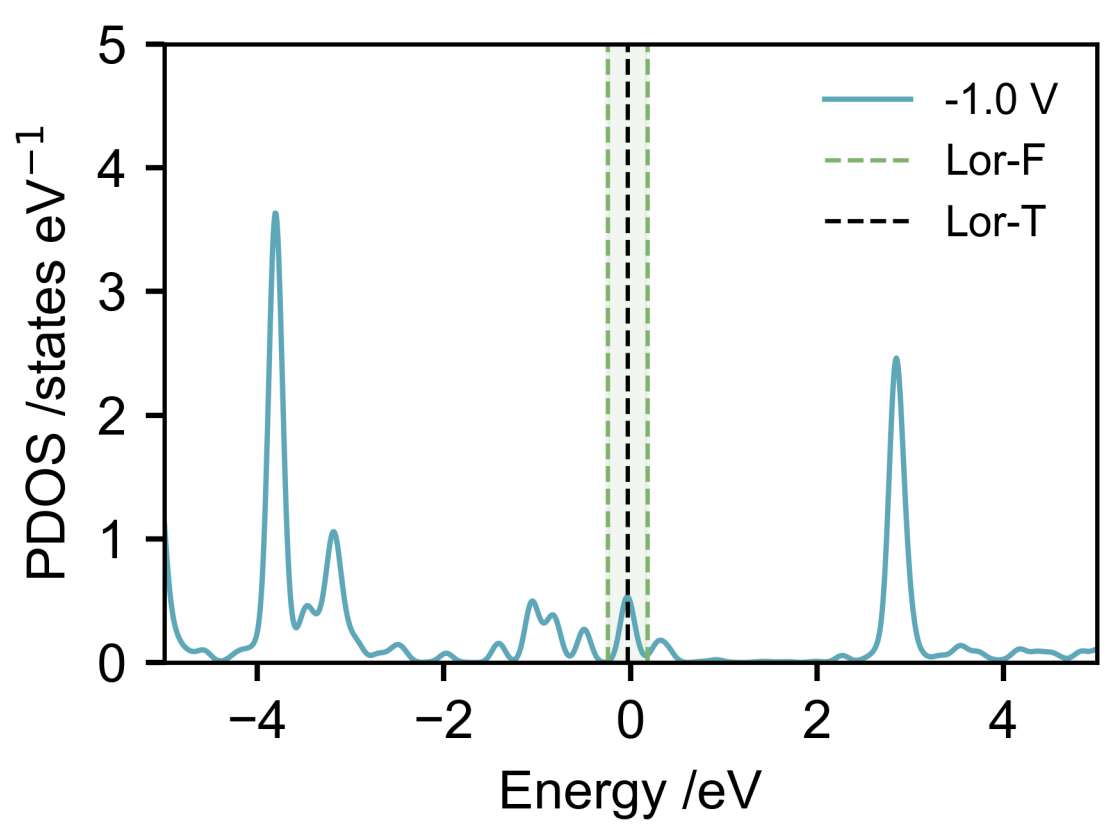

Figure S52. PDOS projected onto COOH s and p-states on Ni-N_4 SACs at -1.0 V. The peak is fitted with a Lorentzian function (Lor-F), with the integration boundary indicated by green dotted vertical lines. The corresponding integrated area is shaded in light green, while the peak maximum (Lor-T) is marked by a black dotted vertical line.

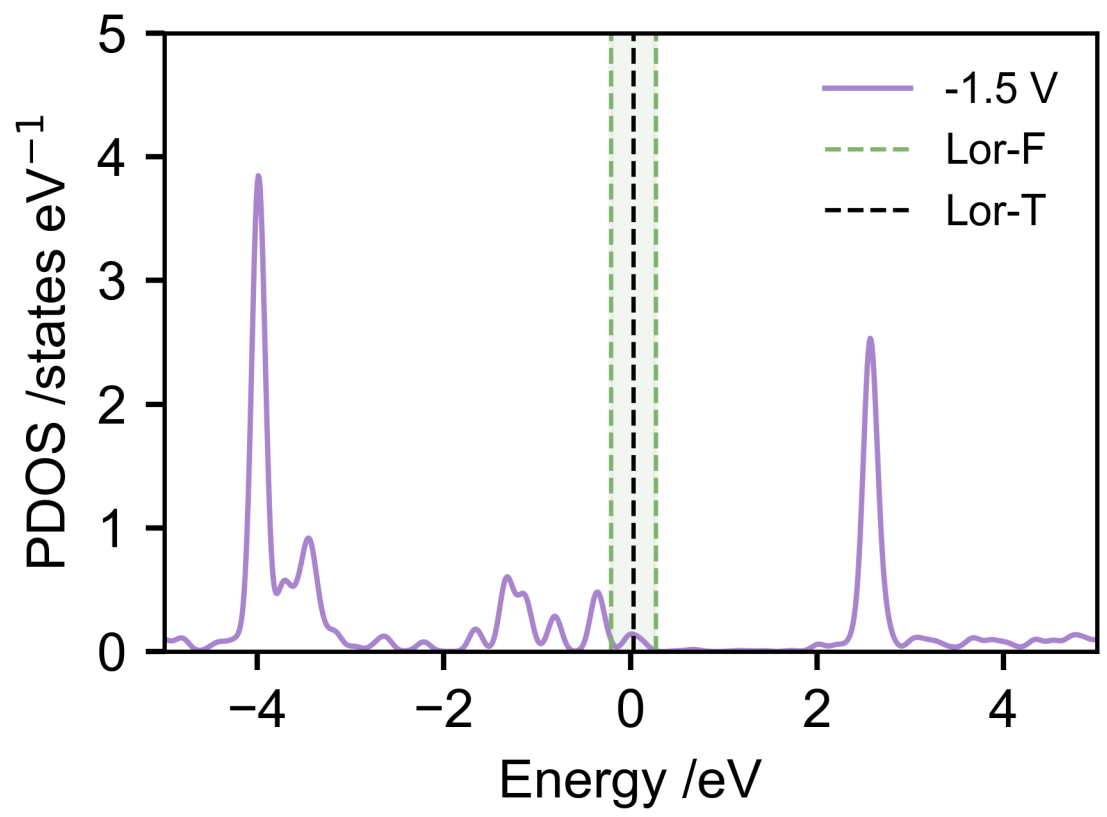

Figure S53. PDOS projected onto COOH s and p-states on Ni-N_4 SACs at -1.5 V. The peak is fitted with a Lorentzian function (Lor-F), with the integration boundary indicated by green dotted vertical lines. The corresponding integrated area is shaded in light green, while the peak maximum (Lor-T) is marked by a black dotted vertical line.

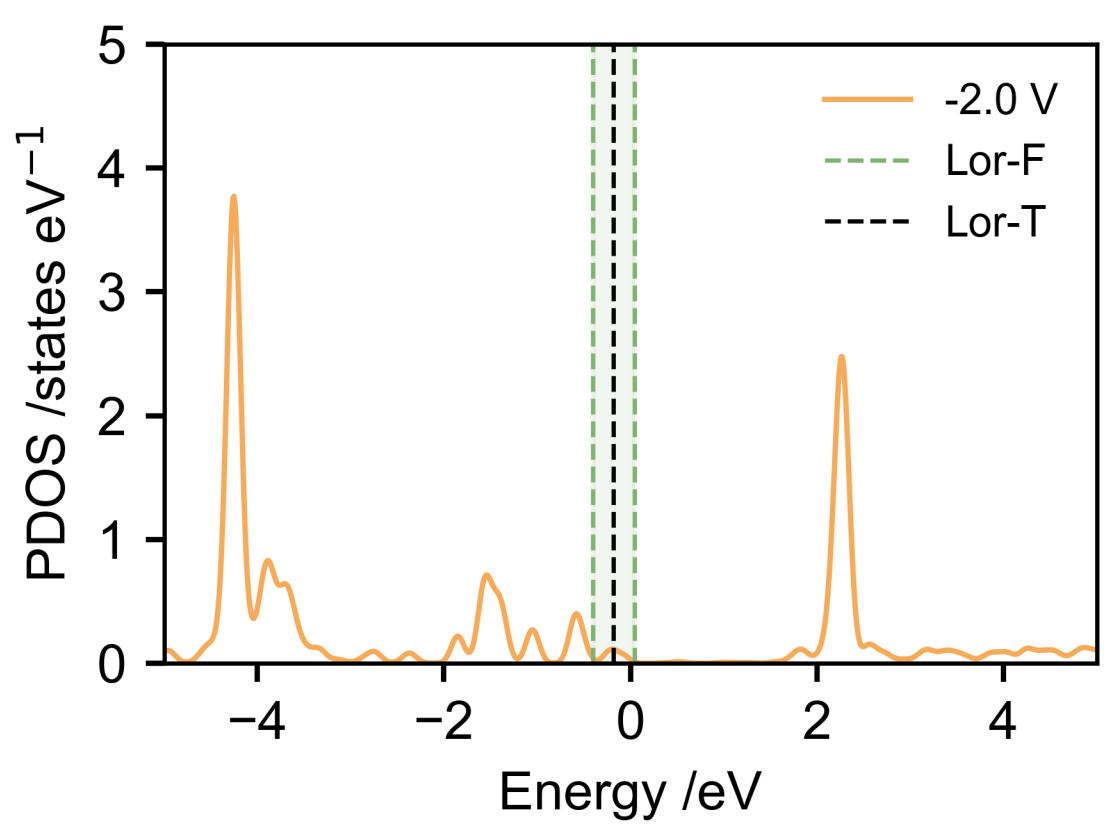

Figure S54. PDOS projected onto COOH s and p-states on Ni-N_4 SACs at -2.0 V. The peak is fitted with a Lorentzian function (Lor-F), with the integration boundary indicated by green dotted vertical lines. The corresponding integrated area is shaded in light green, while the peak maximum (Lor-T) is marked by a black dotted vertical line.

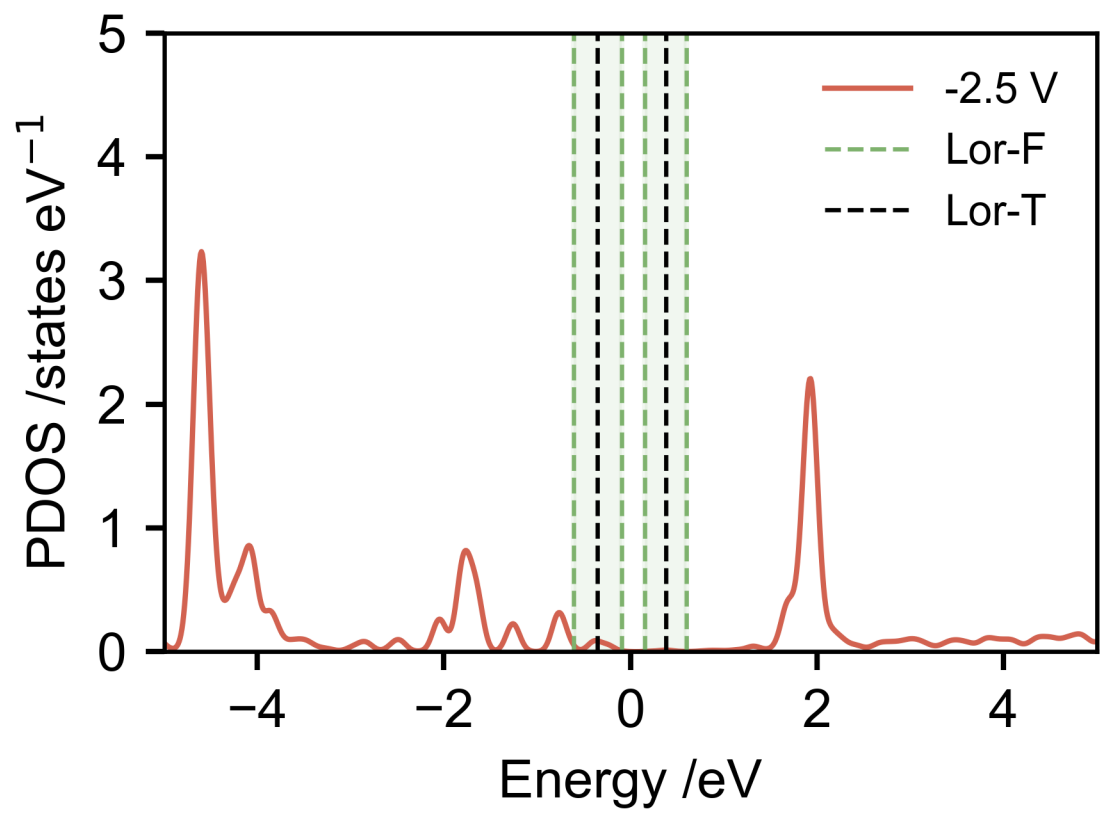

Figure S55. PDOS projected onto COOH s and p-states on Ni-N_4 SACs at -2.5 V. The peak is fitted with a Lorentzian function (Lor-F), with the integration boundary indicated by green dotted vertical lines. The corresponding integrated area is shaded in light green, while the peak maximum (Lor-T) is marked by a black dotted vertical line.

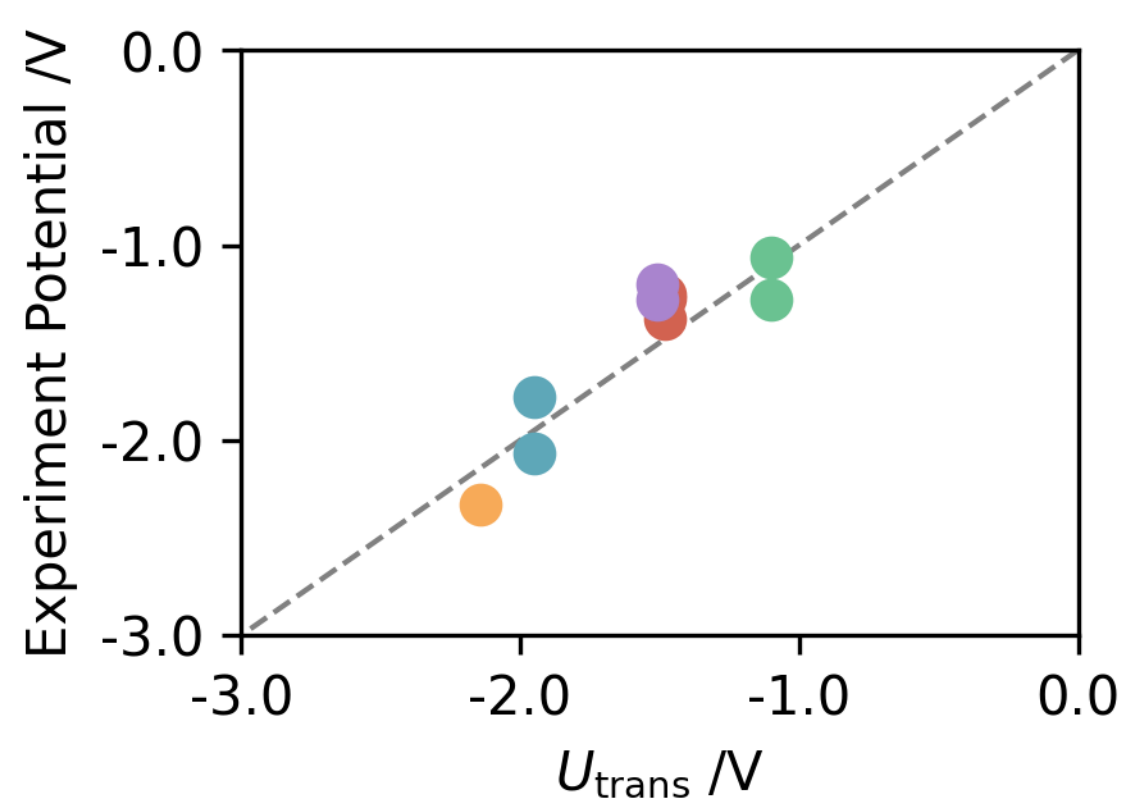

Figure S56. Parity plot comparing the calculated U_{trans} value with the experimental onset potential for CO yield decline across various TM-N_4 SACs (M=Mn, Fe, Ni, Cu, and Zn). Data points are colored by metal center: Mn with green, Fe with red, Ni with blue, Cu with orange, and Zn with purple.

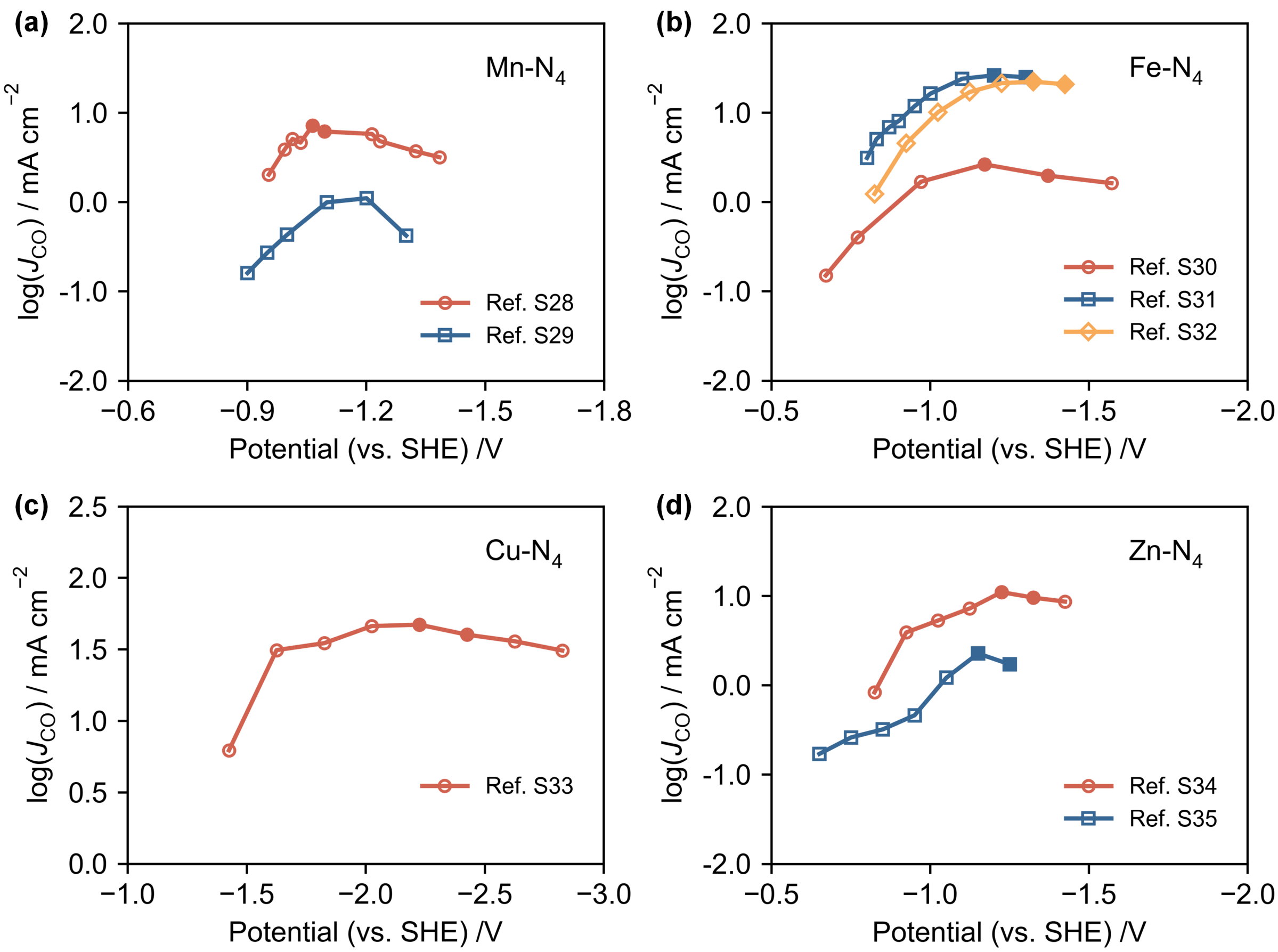

Figure S57. Experimental data set of CO partial current density as a function of applied potential on (a) Mn-N_4,[28,29] (b) Fe-N_4,[30-32] (c) Cu-N_4,[33] and (d) Zn-N_4.[34,35] The two solid symbols on each curve indicate the upper and lower potential limits at which the partial current density begins to decrease, and correspond to the light-colored ranges shown in Figure 6(d). All potentials are converted to values versus SHE to enable comparison across different pH conditions.

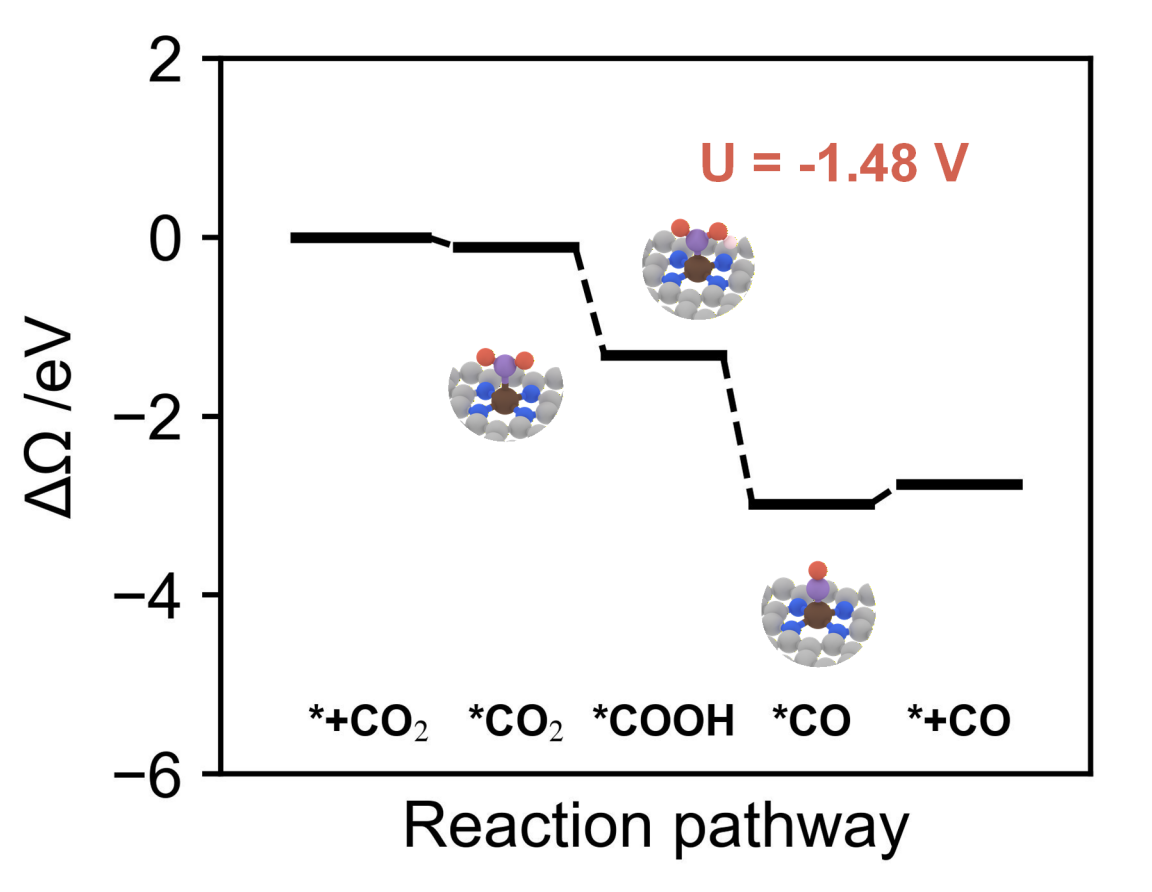

Figure S58. Free energy diagram of Fe-N_4 SACs under U_{trans} = -1.48 V vs. SHE. The C atoms in the graphene substrate are colored gray, while those in CO_2 are rendered in purple. The N, O, H, and Fe atoms are shown in blue, red, pink, and brown, respectively.

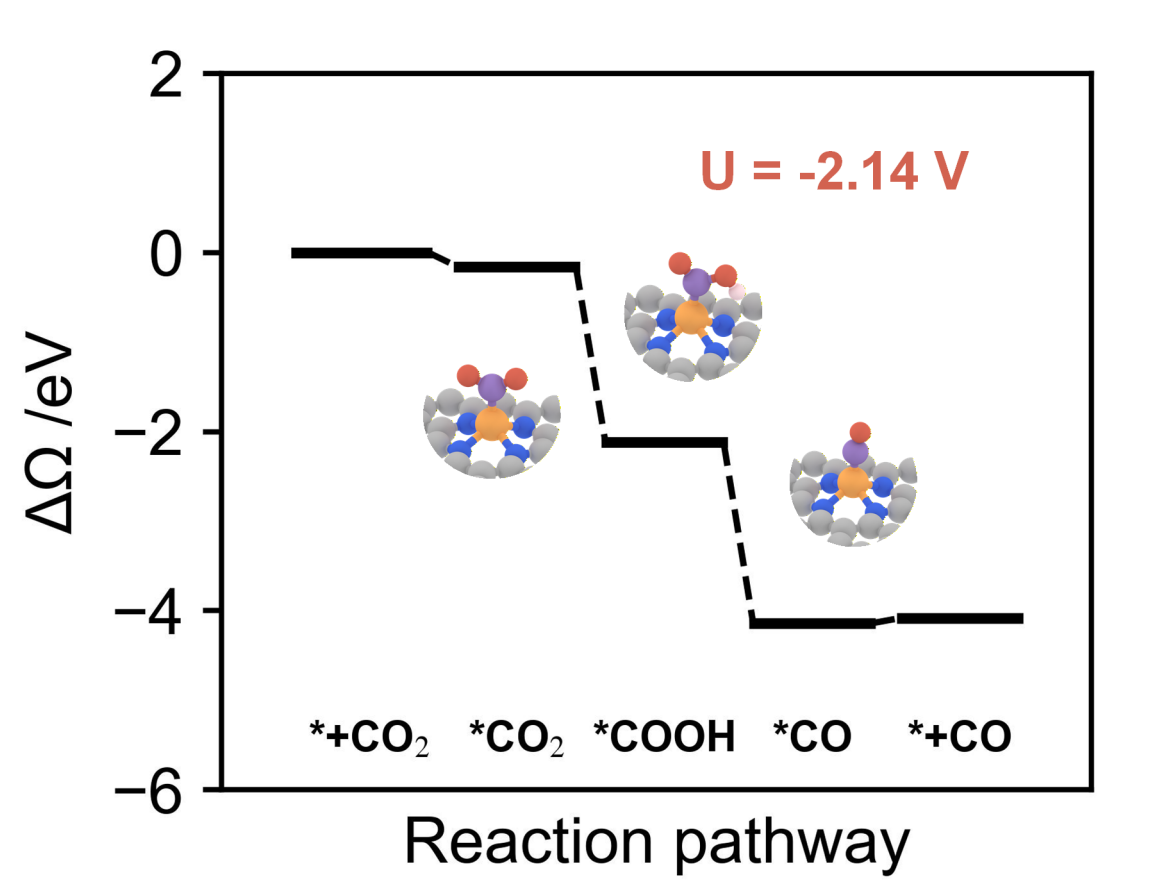

Figure S59. Free energy diagram of Cu-N_4 SACs under U_{trans} = -2.14 V vs. SHE. The C atoms in the graphene substrate are colored gray, while those in CO_2 are rendered in purple. The N, O, H, and Cu atoms are shown in blue, red, pink, and orange, respectively.

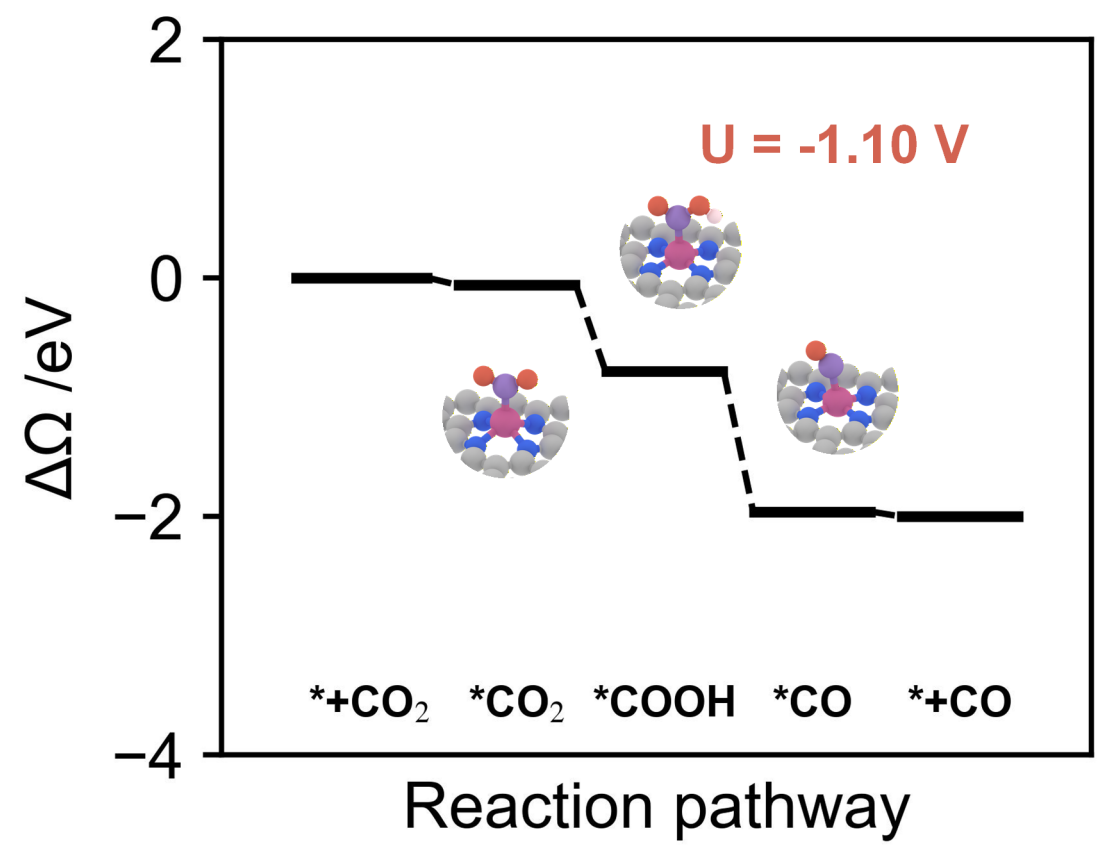

Figure S60. Free energy diagram of Mn-N_4 SACs under U_{trans} = -1.10 V vs. SHE. The C atoms in the graphene substrate are colored gray, while those in CO_2 are rendered in purple. The N, O, H, and Mn atoms are shown in blue, red, pink, and magenta, respectively.

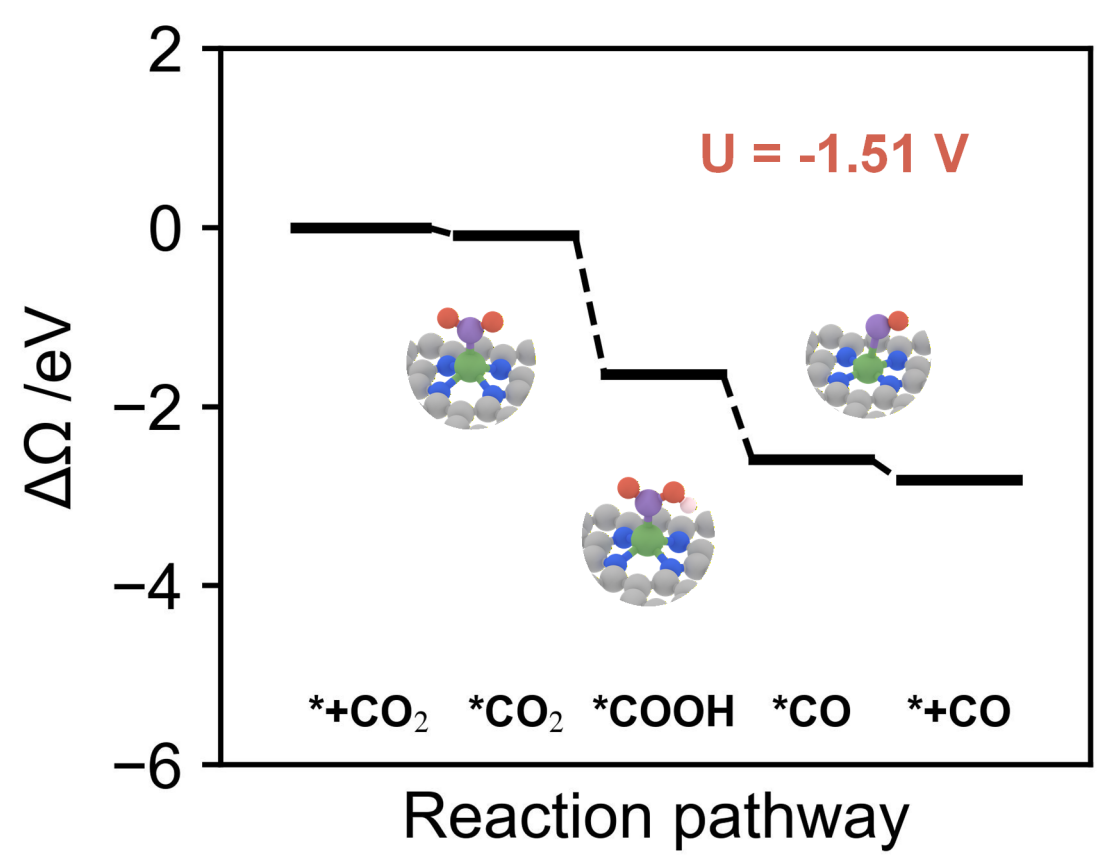

Figure S61. Free energy diagram of Zn-N_4 SACs under U_{trans} = -1.51 V vs. SHE. The C atoms in the graphene substrate are colored gray, while those in CO_2 are rendered in purple. The N, O, H, and Zn atoms are shown in blue, red, pink, and green, respectively.

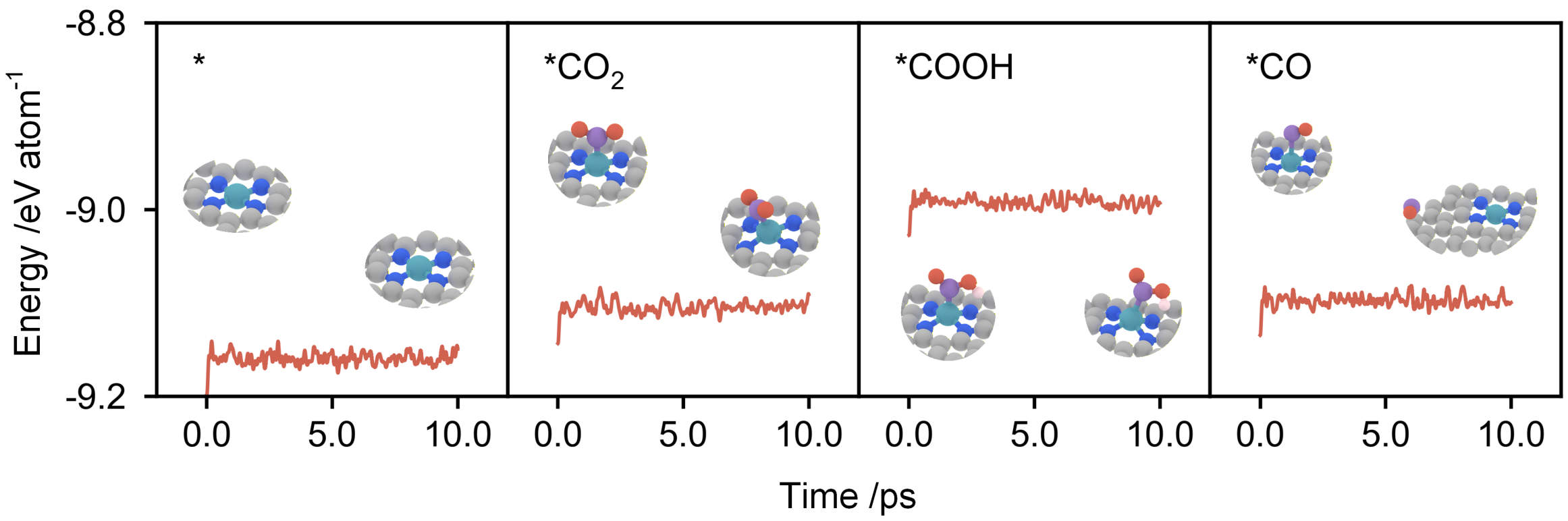

Figure S62. Energy evolution on Ni-N$_4$ SAC at U_{trans} under 300 K. The C atoms in the graphene substrate are colored gray, while those in CO_2 are rendered in purple. The N, O, H, and Ni atoms are shown in blue, red, pink, and cyan, respectively.

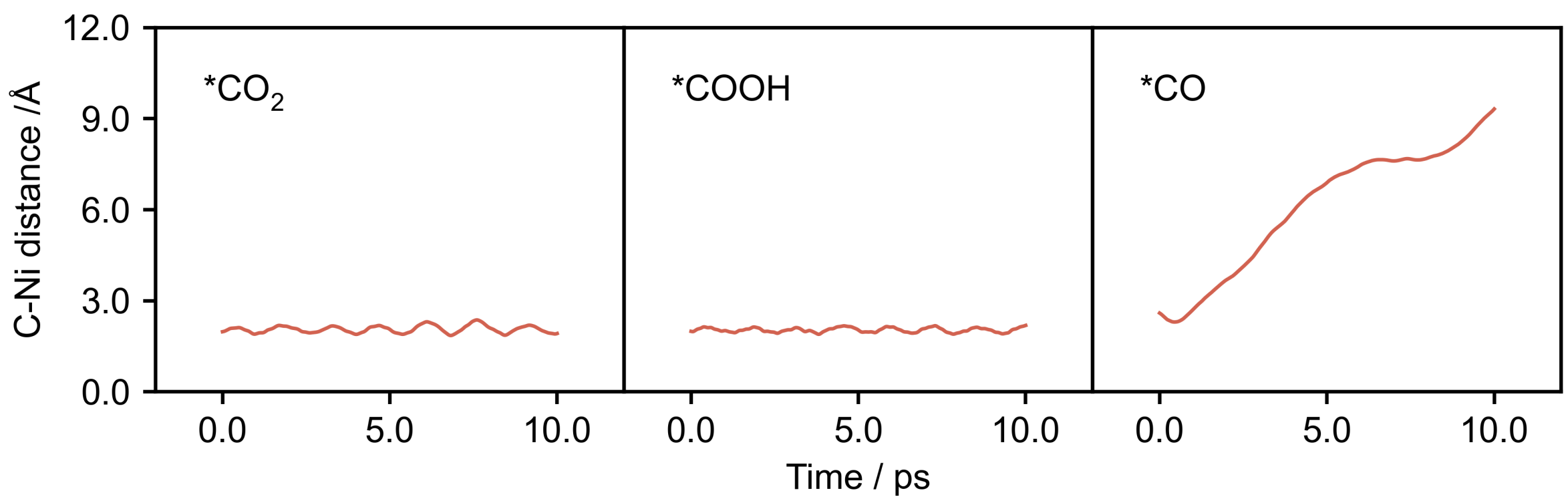

Figure S63. C-Ni distance variation on Ni-N$_4$ SAC at U_{trans} under 300 K.

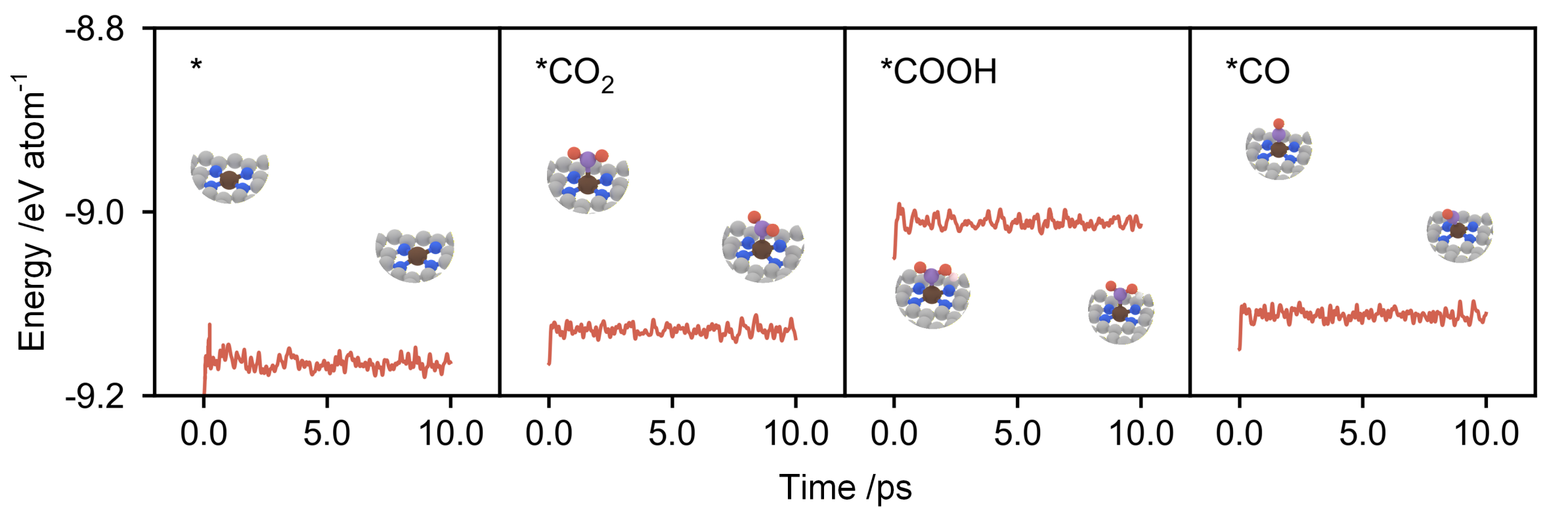

Figure S64. Energy evolution on Fe-N$_4$ SAC at U_{trans} under 300 K. The C atoms in the graphene substrate are colored gray, while those in CO_2 are rendered in purple. The N, O, H, and Fe atoms are shown in blue, red, pink, and brown, respectively.

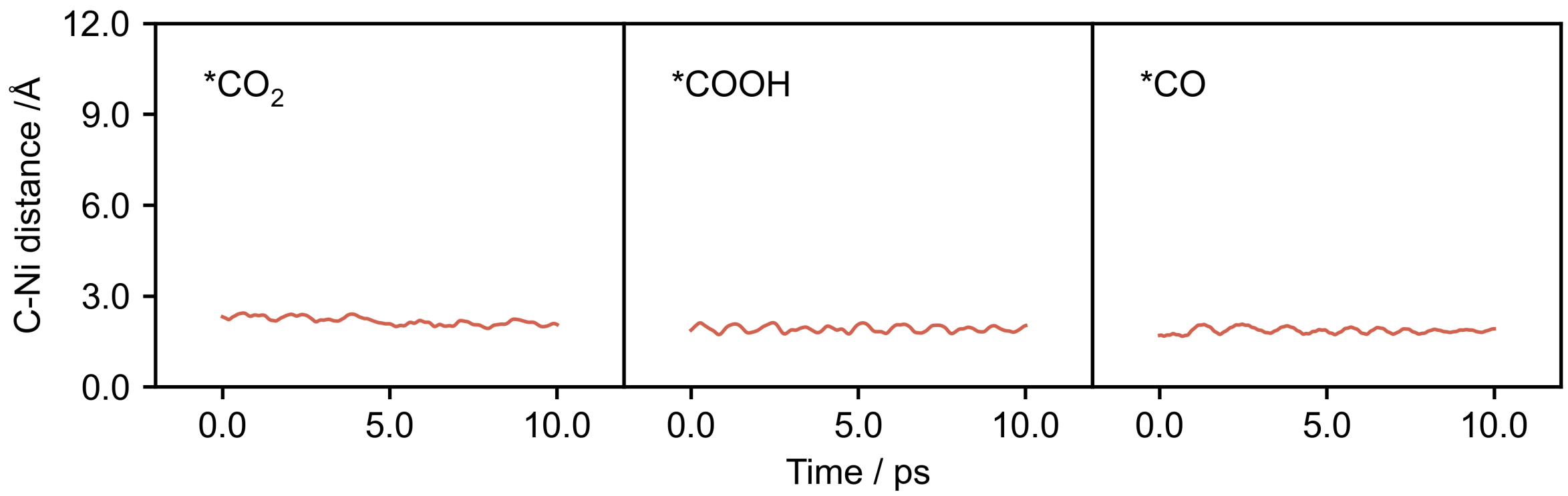

Figure S65. C-Fe distance variation on Fe-N$_4$ SAC at U_{trans} under 300 K.

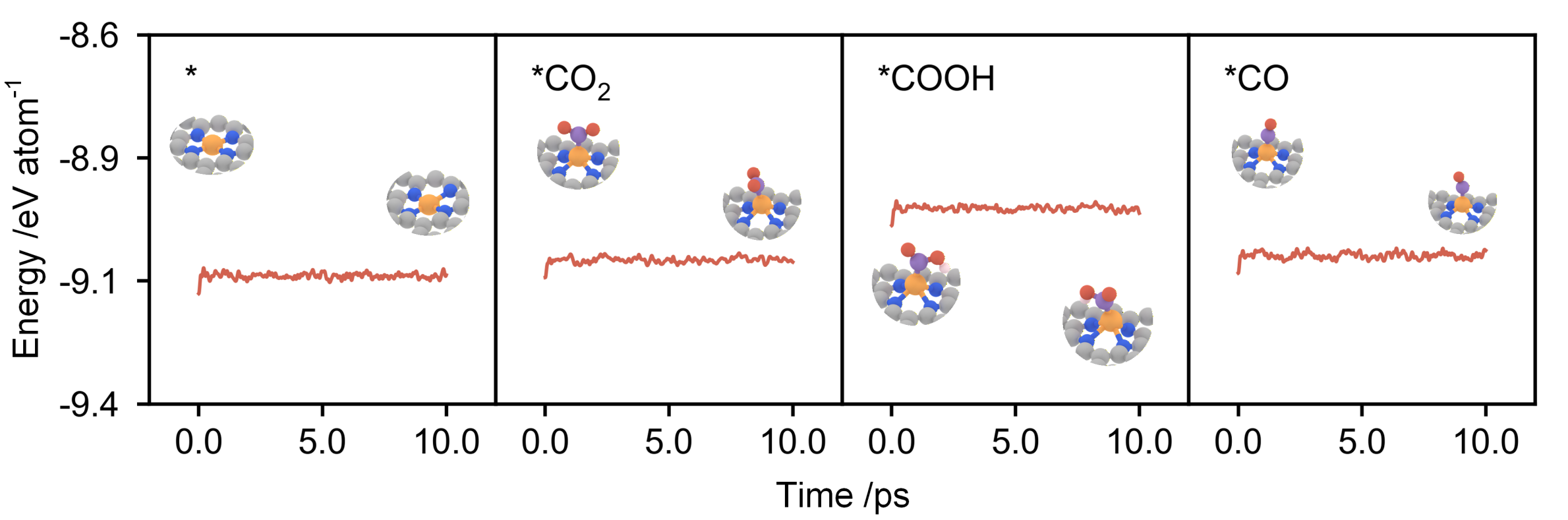

Figure S66. Energy evolution on Cu-N_4 SAC at U_{trans} under 300 K. The C atoms in the graphene substrate are colored gray, while those in CO_2 are rendered in purple. The N, O, H, and Cu atoms are shown in blue, red, pink, and orange, respectively.

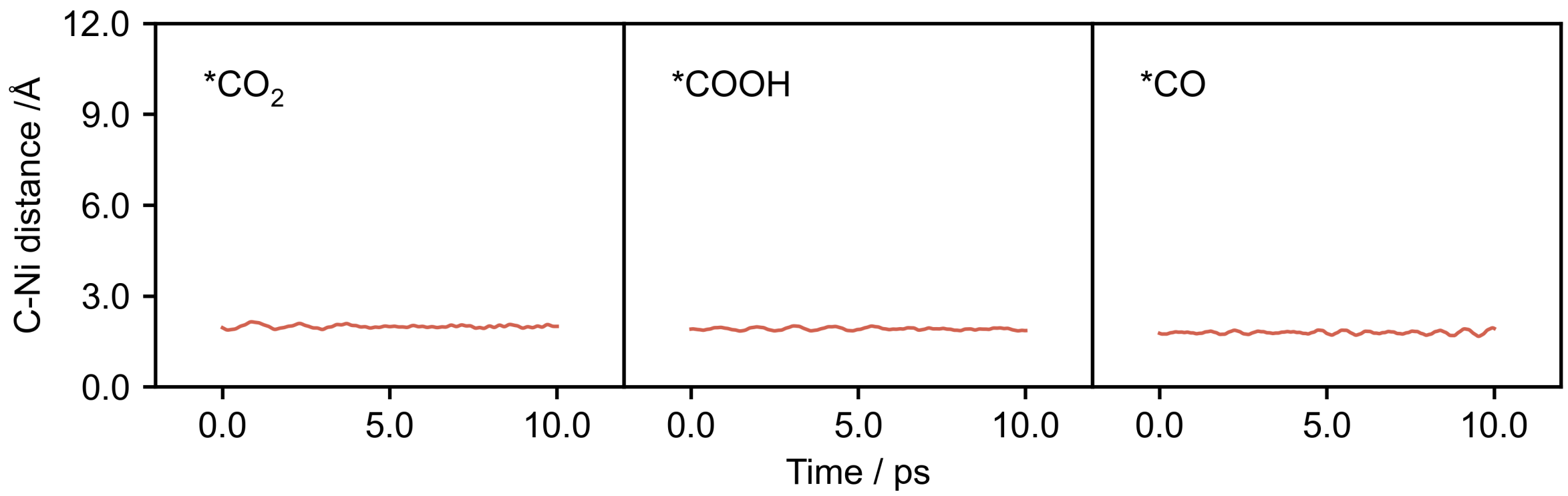

Figure S67. C-Cu distance variation on Cu-N_4 SAC at U_{trans} under 300 K.

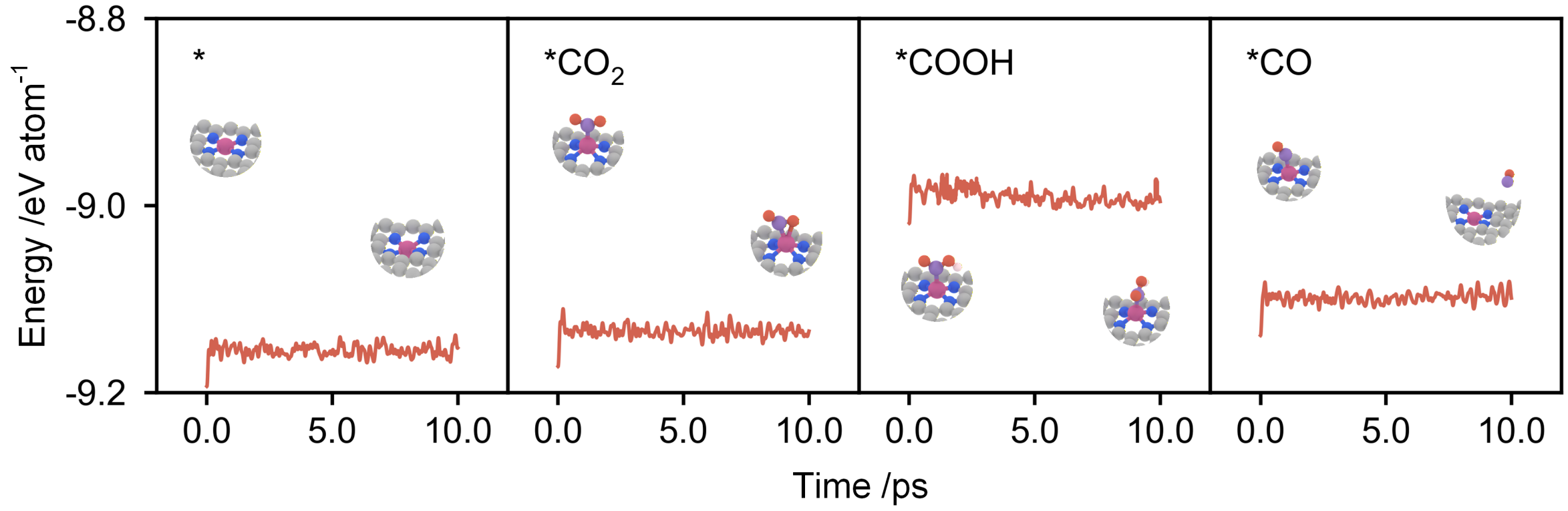

Figure S68. Energy evolution on Mn-N₄ SAC at U_{trans} under 300 K. The C atoms in the graphene substrate are colored gray, while those in CO_2 are rendered in purple. The N, O, H, and Mn atoms are shown in blue, red, pink, and magenta, respectively.

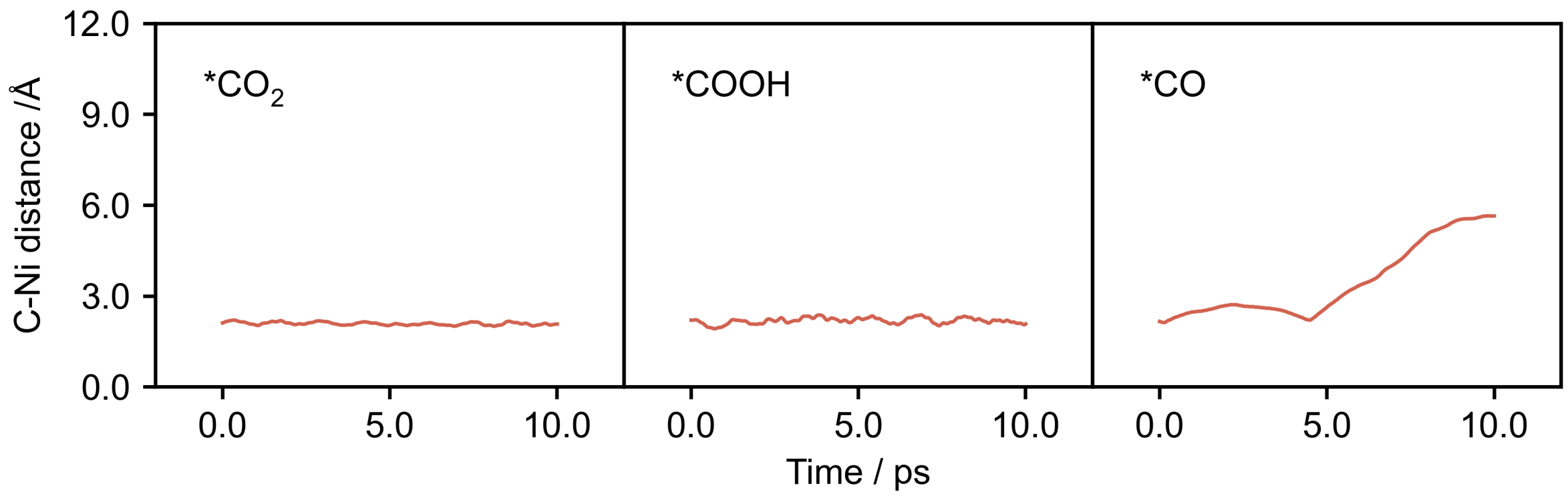

Figure S69. C-Mn distance variation on Mn-N_4 SAC at U_{trans} under 300 K.

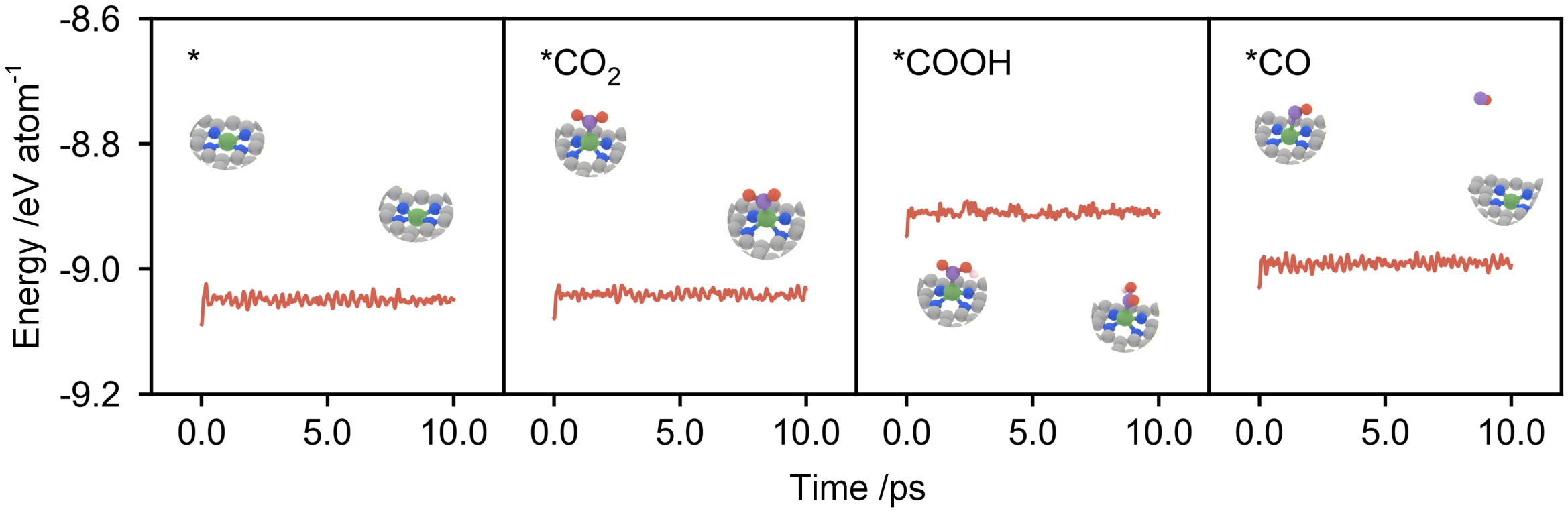

Figure S70. Energy evolution on Zn-N4 SAC at U_{trans} under 300 K. The C atoms in the graphene substrate are colored gray, while those in CO_2 are rendered in purple. The N, O, H, and Zn atoms are shown in blue, red, pink, and green, respectively.

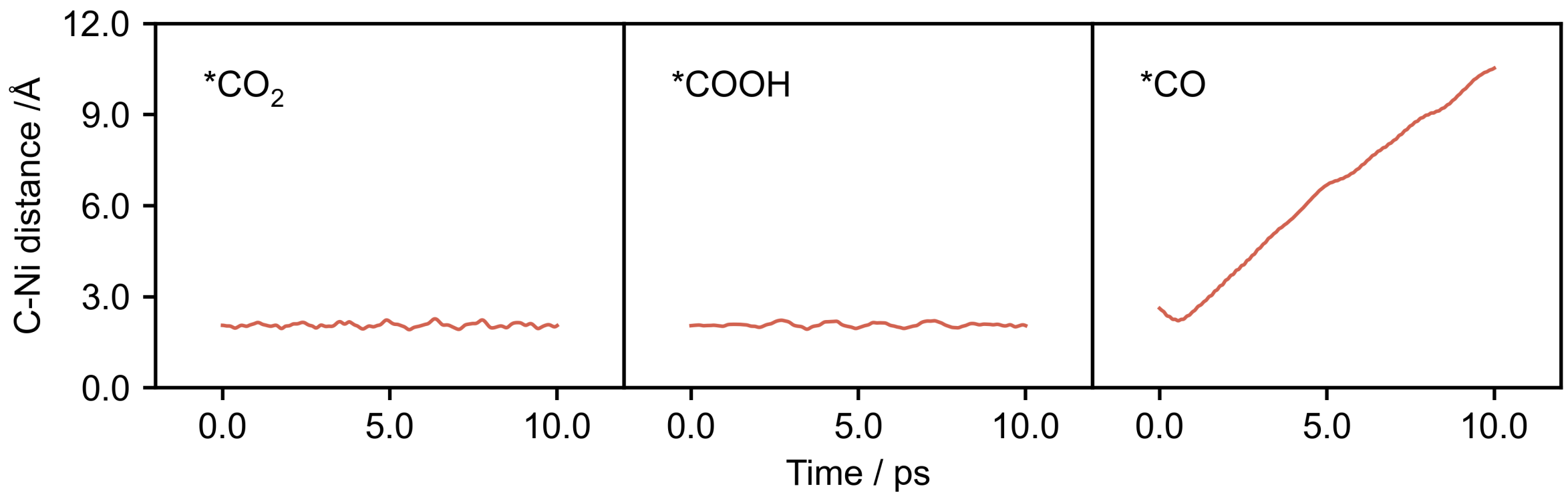

Figure S71. C-Zn distance variation on Zn-N4 SAC at U_{trans} under 300 K.

Table S1. The values of U parameters for DFT calculations.[36,37]

3d	Sc	Ti	V	Cr	Mn	Fe	Co	Ni	Cu	Zn
U	2.11	2.58	2.72	2.79	3.06	3.29	3.42	3.40	3.87	4.12

Table S2. Spin initialization results for CO_2 adsorption on Ni-N_4 SAC starting from chemisorption at -1.5 V.

U /V	Magnetic moment		Energy /eV	Adsorption Mode	
	Initial	Final		Initial	Final
-1.5	0.00	0.96	-469.69	chemisorption	chemisorption
	1.00	0.00	-469.96	chemisorption	physisorption
	2.00	0.00	-469.96	chemisorption	physisorption
	3.00	0.00	-469.96	chemisorption	physisorption
	4.00	0.97	-469.69	chemisorption	chemisorption
	5.00	0.00	-469.96	chemisorption	physisorption

Table S3. Spin initialization results for CO_2 adsorption on Ni-N_4 SAC starting from physisorption at -1.5 V.

U /V	Magnetic moment		Energy /eV	Adsorption Mode	
	Initial	Final		Initial	Final
-1.5	0.00	0.00	-469.96	physisorption	physisorption
	1.00	0.00	-469.96	physisorption	physisorption
	2.00	0.00	-469.96	physisorption	physisorption
	3.00	0.00	-469.96	physisorption	physisorption
	4.00	0.00	-469.96	physisorption	physisorption
	5.00	0.00	-469.96	physisorption	physisorption

Table S4. Spin initialization results for CO_2 adsorption on Ni-N_4 SAC starting from chemisorption at -2.0 V.

U /V	Magnetic moment		Energy /eV	Adsorption Mode	
	Initial	Final		Initial	Final
-2.0	0.00	-1.04	-470.58	chemisorption	chemisorption
	1.00	0.00	-470.55	chemisorption	physisorption
	2.00	0.00	-470.55	chemisorption	physisorption
	3.00	1.04	-470.58	chemisorption	chemisorption
	4.00	1.04	-470.58	chemisorption	chemisorption
	5.00	0.00	-470.55	chemisorption	physisorption

Table S5. Spin initialization results for CO_2 adsorption on Ni-N_4 SAC starting from physisorption at -2.0 V.

U /V	Magnetic moment		Energy /eV	Adsorption Mode	
	Initial	Final		Initial	Final
-2.0	0.00	0.00	-470.55	physisorption	physisorption
	1.00	0.00	-470.55	physisorption	physisorption
	2.00	0.00	-470.55	physisorption	physisorption
	3.00	0.00	-470.55	physisorption	physisorption
	4.00	0.00	-470.55	physisorption	physisorption
	5.00	0.00	-470.55	physisorption	physisorption

Table S6. Spin initialization results for CO_2 adsorption on Ni-N_4 SAC starting from chemisorption at -2.5 V.

U /V	Magnetic moment		Energy /eV	Adsorption Mode	
	Initial	Final		Initial	Final
-2.5	0.00	-0.25	-471.45	chemisorption	chemisorption
	1.00	-0.24	-471.45	chemisorption	chemisorption
	2.00	0.22	-471.45	chemisorption	chemisorption
	3.00	1.06	-471.83	chemisorption	chemisorption
	4.00	1.05	-471.83	chemisorption	chemisorption
	5.00	-1.06	-471.83	chemisorption	chemisorption

Table S7. Spin initialization results for CO_2 adsorption on Ni-N_4 SAC starting from physisorption at -2.5 V.

U /V	Magnetic moment		Energy /eV	Adsorption Mode	
	Initial	Final		Initial	Final
-2.5	0	0.00	-471.50	physisorption	physisorption
	1	0.00	-471.50	physisorption	physisorption
	2	0.00	-471.50	physisorption	physisorption
	3	0.00	-471.50	physisorption	physisorption
	4	0.00	-471.50	physisorption	physisorption
	5	0.00	-471.50	physisorption	physisorption

Table S8. OMC-constrained energies (eV) of NiN_4-SAC with chemisorbed (chem) and physisorbed (phys) CO_2 at applied potentials of -1.5, -2.0, and -2.5 V. Five distinct prescribed Ni $3d$-orbital occupation patterns were examined, comprising one low-spin configuration (LS) and four high-spin configurations (HS-1 to HS-4). The lowest-energy configuration at each condition is highlighted in bold.

	-1.5 V		-2.0 V		-2.5 V	
	chem	phys	chem	phys	chem	phys
LS	-475.51	**-474.16**	-477.14	**-476.10**	-478.94	**-477.64**
HS-1	-475.46	-472.49	-477.30	-474.62	-479.12	-476.22
HS-2	-475.41	-472.99	-477.13	-474.98	-478.83	-476.61
HS-3	-475.54	-473.34	-477.28	-475.46	-479.12	-477.13
HS-4	**-475.59**	-472.57	**-477.34**	-474.65	**-479.18**	-476.27

Note. For chemisorbed CO_2, HS-4 is consistently the lowest-energy OMC configuration at all three examined potentials, whereas the LS configuration is consistently lowest in energy for physisorbed CO_2. For each condition, the lowest-energy OMC solution was subsequently used as the initial state for a fully unconstrained optimization after removal of the occupation constraint. These unconstrained calculations recovered the same electronic states as those obtained in the original calculations, supporting the robustness of the identified states with respect to the initial Ni -orbital occupation.

Table S9. The values of U_{trans} in V for 3d TM-N_4 SACs.

TM	Sc	Ti	V	Cr	Mn	Fe	Co	Ni	Cu	Zn
U_{trans}	-1.58	-0.69	-0.35	-1.21	-1.10	-1.48	-1.02	-1.95	-2.14	-1.51

Table S10. Surface energy of seven low-index Hg facets.

Hg facet	E_{cleave} (eV/Å^2)	E_{relax} (eV/Å^2)	E_{sur} (eV/Å^2)
(100)	0.028	-0.010	0.018
(010)	0.030	-0.006	0.023
(001)	0.029	-0.009	0.020
(110)	0.030	-0.001	0.029
(101)	0.025	-0.001	0.024
(011)	0.029	-0.008	0.022
(111)	0.029	-0.007	0.022

Table S11. Two-dimensional (2D) SISSO model.

Fitted data	2D SISSO model
Full-data fitting	$U_{trans} = -0.47 * ln\left(\left\|E_{d_b} - E_{x^2-y^2{}_s}\right\| - \left\|E_{yz_s} - E_{z^2{}_s}\right\|\right) - 0.00020 * \frac{{E_{xz_s}}^2 * E_{coh_b}}{R_d - E_{xz_s} + E_{x^2-y^2{}_s}} - 1.56$

Table S12. One-dimensional (1D) SISSO model obtained with leave-one-out cross-validation (LOOCV).

Leaved TM	1D SISSO model resulted from the LOOCV
Sc	$U_{trans} = -0.48 * ln\left(\left\|\left(E_{d_b} - E_{x^2-y^2_s}\right) - \left\|E_{yz_s} - E_{z^2_s}\right\|\right\|\right) - 1.58$
Ti	$U_{trans} = -0.48 * ln\left(\left\|\left(E_{d_b} - E_{x^2-y^2_s}\right) - \left\|E_{yz_s} - E_{z^2_s}\right\|\right\|\right) - 1.59$
V	$U_{trans} = -1.59 * \sqrt[3]{\left\|E_{d_b} - E_{x^2-y^2_s}\right\| - \left\|E_{yz_s} - E_{z^2_s}\right\|} + 0.06$
Cr	$U_{trans} = -0.48 * ln\left(\left\|\left(E_{d_b} - E_{x^2-y^2_s}\right) - \left\|E_{yz_s} - E_{z^2_s}\right\|\right\|\right) - 1.58$
Mn	$U_{trans} = -0.48 * ln\left(\left\|\left(E_{d_b} - E_{x^2-y^2_s}\right) - \left\|E_{yz_s} - E_{z^2_s}\right\|\right\|\right) - 1.58$
Fe	$U_{trans} = -0.49 * ln\left(\left\|\left(E_{d_b} - E_{x^2-y^2_s}\right) - \left\|E_{yz_s} - E_{z^2_s}\right\|\right\|\right) - 1.59$
Co	$U_{trans} = 0.44 * \left[\left(E_{d_b} - E_{xy_s}\right) - E_{z^2_s}\right] * \frac{R_s * N_{x^2-y^2_s}}{E_{xy_s}} - 0.36$
Ni	$U_{trans} = -0.48 * ln\left(\left\|\left(E_{d_b} - E_{x^2-y^2_s}\right) - \left\|E_{yz_s} - E_{z^2_s}\right\|\right\|\right) - 1.59$
Cu	$U_{trans} = -0.46 * ln\left(\left\|\left(E_{d_b} - E_{x^2-y^2_s}\right) - \left\|E_{yz_s} - E_{z^2_s}\right\|\right\|\right) - 1.55$
Zn	$U_{trans} = -0.48 * ln\left(\left\|\left(E_{d_b} - E_{x^2-y^2_s}\right) - \left\|E_{yz_s} - E_{z^2_s}\right\|\right\|\right) - 1.59$

Table S13. 2D SISSO model obtained with LOOCV.

Leaved TM	2D SISSO model resulted from the LOOCV
Sc	$U_{trans} = -0.48 * ln\left(\left\|\left(E_{d_b} - E_{x^2-y^2_s}\right) - \left\|E_{yz_s} - E_{z^2_s}\right\|\right\|\right) + 0.47 * \frac{\left\|E_{yz_s}/E_{xy_s} - N_{x^2-y^2_s}/N_{xy_s}\right\|}{E_{b_a} * R_s} - 1.70$
Ti	$U_{trans} = -0.48 * ln\left(\left\|\left(E_{d_b} - E_{x^2-y^2_s}\right) - \left\|E_{yz_s} - E_{z^2_s}\right\|\right\|\right) - 0.04 * \frac{\left\|E_{yz_s} - E_{z^2_s}\right\|}{E_{coh_b}/E_{F_s} - E_{z^2_s}/E_{xy_s}} - 1.56$
V	$U_{trans} = -1.60 * \sqrt[3]{\left\|E_{d_b} - E_{x^2-y^2_s}\right\| - \left\|E_{yz_s} - E_{z^2_s}\right\|} - 0.07 * \left\|\left\|E_{xy_s} - 2 * E_{xz_s}\right\| - \left\|E_{xz_s} - 2 * E_{x^2-y^2_s}\right\|\right\| - 0.03$
Cr	$U_{trans} = -0.47 * ln\left(\left\|\left(E_{d_b} - E_{x^2-y^2_s}\right) - \left\|E_{yz_s} - E_{z^2_s}\right\|\right\|\right) - 0.15 * \frac{\left(E_{yz_s} - E_{z^2_s}\right)/\left(N_{xy_s} - N_{yz_s}\right)}{\left\|E_{d_b} - E_{coh_b}\right\|} - 1.57$
Mn	$U_{trans} = -0.47 * ln\left(\left\|\left(E_{d_b} - E_{x^2-y^2_s}\right) - \left\|E_{yz_s} - E_{z^2_s}\right\|\right\|\right) + 0.00012 * \frac{E_{coh_b} * N_{yz_s}}{\left\|N_{yz_s} - N_{xz_s}\right\|} * \left(E_{1_a} + E_{x^2-y^2_s}\right) - 1.51$
Fe	$U_{trans} = -0.48 * ln\left(\left\|\left(E_{d_b} - E_{x^2-y^2_s}\right) - \left\|E_{yz_s} - E_{z^2_s}\right\|\right\|\right) + 0.06 * \frac{\left(E_{yz_s} - E_{z^2_s}\right) * E_{x^2-y^2_s}/N_{yz_s}}{E_{coh_b} - E_{F_s}} - 1.57$
Co	$U_{trans} = 0.44 * \left[\left(E_{d_b} - E_{xy_s}\right) - E_{z^2_s}\right] * \frac{R_s * N_{x^2-y^2}}{E_{xy}} - 0.0056 * \frac{E_{b_a} + E_{coh_b} + E_{F_s}}{M/E_{1_a}^{3}} - 0.56$
Ni	$U_{trans} = -0.48 * ln\left(\left\|\left(E_{d_b} - E_{x^2-y^2_s}\right) - \left\|E_{yz_s} - E_{z^2_s}\right\|\right\|\right) - 0.0057 * E_{b_a} * E_{coh_b} * N_{z^2_s} * ln\left(\left\|E_{yz_s} - E_{xz_s}\right\|\right) - 1.52$
Cu	$U_{trans} = -0.45 * ln\left(\left\|\left(E_{d_b} - E_{x^2-y^2_s}\right) - \left\|E_{yz_s} - E_{z^2_s}\right\|\right\|\right) + 0.38 * \frac{N_{xy_s} * E_{xz_s}/E_{yz_s}}{N_{s+d} - 2 * N_{xz_s}} - 1.64$
Zn	$U_{trans} = -0.48 * ln\left(\left\|\left(E_{d_b} - E_{x^2-y^2_s}\right) - \left\|E_{yz_s} - E_{z^2_s}\right\|\right\|\right) - 0.061 * \left\|\frac{\left\|E_{yz_s} - E_{F_s}\right\|}{E_{coh_b} - E_{x^2-y^2_s}} - \frac{E_{yz_s}}{E_{b_a}}\right\| - 1.50$

Table S14. Held-out U_{trans} predictions for each TM-N_4 system obtained from 1D and 2D SISSO leave-one-out cross-validation (LOOCV).

Leaved TM	DFT value	1D prediction	2D prediction
Sc	-1.58	-1.61	-0.32
Ti	-0.69	-0.64	-0.63
V	-0.35	-0.62	-0.81
Cr	-1.21	-1.21	-1.15
Mn	-1.10	-1.10	-1.64
Fe	-1.48	-1.55	-1.52
Co	-1.02	-1.32	-1.21
Ni	-1.95	-1.97	-2.23
Cu	-2.14	-2.03	-1.98
Zn	-1.51	-1.56	-3.66

References:

(1) Butler, J.; Henglein, A. Elementary reactions of the reduction of Tl^+ in aqueous solution. *Radiat. Phys. Chem.* **1980**, *15* (5), 603-612.

(2) Koppenol, W.; Rush, J. Reduction potential of the carbon dioxide/carbon dioxide radical anion: a comparison with other C1 radicals. *J. Phys. Chem.* **1987**, *91* (16), 4429-4430.

(3) Schneider, J.; Jia, H.; Muckerman, J. T.; Fujita, E. Thermodynamics and kinetics of CO_2, CO, and H^+ binding to the metal centre of CO_2 reduction catalysts. *Chem. Soc. Rev.* **2012**, *41* (6), 2036-2051.

(4) Schichtl, Z. G.; Carvalho, O. Q.; Tan, J.; Saund, S. S.; Ghoshal, D.; Wilder, L. M.; Gish, M. K.; Nielander, A. C.; Stevens, M. B.; Greenaway, A. L. Chemistry of materials underpinning photoelectrochemical solar fuel production. *Chem. Rev.* **2025**, *125* (10), 4768-4839.

(5) Meites, L. *Polarographic techniques*; 2nd ed.; Wiley-Interscience, 1965.

(6) Lilie, J.; Beck, G.; Henglein, A. Pulsradiolyse und Polarographie: Halbstufenpotentiale für die Oxydation und Reduktion von kurzlebigen organischen Radikalen an der Hg-Elektrode. *Ber. Bunsenges. Phys. Chem.* **1971**, *75* (5), 458-465.

(7) Jain, A.; Ong, S. P.; Hautier, G.; Chen, W.; Richards, W. D.; Dacek, S.; Cholia, S.; Gunter, D.; Skinner, D.; Ceder, G.; Persson, K. A. Commentary: The Materials Project: A materials genome approach to accelerating materials innovation. *APL Mater.* **2013**, *1* (1), 011002-011012.

(8) Horton, M. K.; Huck, P.; Yang, R. X.; Munro, J. M.; Dwaraknath, S.; Ganose, A. M.; Kingsbury, R. S.; Wen, M.; Shen, J. X.; Mathis, T. S.; Kaplan, A. D.; Berket, K.; Riebesell, J.; George, J.; Rosen, A. S.; Spotte-Smith, E. W. C.; McDermott, M. J.; Cohen, O. A.; Dunn, A.; Kuner, M. C.; Rignanese, G.-M.; Petretto, G.; Waroquiers, D.; Griffin, S. M.; Neaton, J. B.; Chrzan, D. C.; Asta, M.; Hautier, G.; Cholia, S.; Ceder, G.; Ong, S. P.; Jain, A.; Persson, K. A. Accelerated data-driven materials science with the Materials Project. *Nat. Commun.* **2025**, *24* (10), 1522-1532.

(9) Gauthier, J. A.; Fields, M.; Bajdich, M.; Chen, L. D.; Sandberg, R. B.; Chan, K.; Nørskov, J. K. Facile electron transfer to CO_2 during adsorption at the metal| solution interface. *J. Phys. Chem. C* **2019**, *123* (48), 29278-29283.

(10) Vijay, S.; Ju, W.; Brückner, S.; Tsang, S.-C.; Strasser, P.; Chan, K. Unified mechanistic understanding of CO_2 reduction to CO on transition metal and single atom catalysts. *Nat. Catal.* **2021**, *4* (12), 1024-1031.

(11) Hu, X.-M.; Hval, H. H.; Bjerglund, E. T.; Dalgaard, K. J.; Madsen, M. R.; Pohl, M.-M.; Welter, E.; Lamagni, P.; Buhl, K. B.; Bremholm, M. Selective CO_2 reduction to CO in water using earth-abundant metal and nitrogen-doped carbon electrocatalysts. *ACS Catal.* **2018**, *8* (7), 6255-6264.

(12) Zheng, T.; Jiang, K.; Ta, N.; Hu, Y.; Zeng, J.; Liu, J.; Wang, H. Large-scale and highly selective CO_2 electrocatalytic reduction on nickel single-atom catalyst. *Joule* **2019**, *3* (1), 265-278.

(13) Möller, T.; Ju, W.; Bagger, A.; Wang, X.; Luo, F.; Thanh, T. N.; Varela, A. S.; Rossmeisl, J.; Strasser, P. Efficient CO_2 to CO electrolysis on solid Ni–N–C catalysts at industrial current densities. *Energy Environ. Sci.* **2019**, *12* (2), 640-647.

(14) Ren, W.; Tan, X.; Chen, X.; Zhang, G.; Zhao, K.; Yang, W.; Jia, C.; Zhao, Y.; Smith, S. C.; Zhao, C. Confinement of ionic liquids at single-Ni-sites boost electroreduction of CO_2 in aqueous electrolytes. *ACS Catal.* **2020**, *10* (22), 13171-13178.

(15) Koshy, D. M.; Chen, S.; Lee, D. U.; Stevens, M. B.; Abdellah, A. M.; Dull, S. M.; Chen, G.; Nordlund, D.; Gallo, A.; Hahn, C. Understanding the origin of highly selective CO_2 electroreduction to CO on Ni, N-doped carbon catalysts. *Angew. Chem., Int. Ed.* **2020**, *59* (10), 4043-4050.

(16) Fan, Q.; Hou, P.; Choi, C.; Wu, T. S.; Hong, S.; Li, F.; Soo, Y. L.; Kang, P.; Jung, Y.; Sun, Z. Activation of Ni particles into single Ni–N atoms for efficient electrochemical reduction of CO_2. *Adv. Energy Mater.* **2020**, *10* (5), 1903068.

(17) Zhang, T.; Lin, L.; Li, Z.; He, X.; Xiao, S.; Shanov, V. N.; Wu, J. Nickel–nitrogen–carbon molecular catalysts for high rate CO_2 electro-reduction to CO: on the role of carbon substrate and reaction chemistry. *ACS Appl. Energy Mater.* **2020**, *3* (2), 1617-1626.

(18) Yang, H.; Lin, Q.; Zhang, C.; Yu, X.; Cheng, Z.; Li, G.; Hu, Q.; Ren, X.; Zhang, Q.; Liu, J. Carbon dioxide electroreduction on single-atom nickel decorated carbon membranes with industry compatible current densities. *Nat. Comm.* **2020**, *11* (1), 593.

(19) Rong, X.; Wang, H. J.; Lu, X. L.; Si, R.; Lu, T. B. Controlled synthesis of a vacancy-defect single-atom catalyst for boosting CO_2 electroreduction. *Angew. Chem., Int. Ed.* **2020**, *132* (5), 1977-1981.

(20) Wang, X.; Feng, S.; Lu, W.; Zhao, Y.; Zheng, S.; Zheng, W.; Sang, X.; Zheng, L.; Xie, Y.; Li, Z. A new strategy for accelerating dynamic proton transfer of electrochemical CO_2 reduction at high current densities. *Adv. Funct. Mater.* **2021**, *31* (50), 2104243.

(21) Chen, B.; Li, B.; Tian, Z.; Liu, W.; Liu, W.; Sun, W.; Wang, K.; Chen, L.; Jiang, J. Enhancement of Mass Transfer for Facilitating Industrial-Level CO_2 Electroreduction on Atomic Ni-N_4 Sites. *Adv. Energy Mater.* **2021**, *11* (40), 2102152.

(22) Jeong, G. H.; Tan, Y. C.; Song, J. T.; Lee, G.-Y.; Lee, H. J.; Lim, J.; Jeong, H. Y.; Won, S.; Oh, J.; Kim, S. O. Synthetic multiscale design of nanostructured Ni single atom catalyst for superior CO_2 electroreduction. *Chem. Eng. J.* **2021**, *426*, 131063.

(23) Hao, Q.; Tang, Q.; Zhong, H.-X.; Wang, J.-Z.; Liu, D.-X.; Zhang, X.-B. Fully exposed nickel clusters with electron-rich centers for high-performance electrocatalytic CO_2 reduction to CO. *Sci. Bull.* **2022**, *67* (14), 1477-1485.

(24) Li, Y.; Adli, N. M.; Shan, W.; Wang, M.; Zachman, M. J.; Hwang, S.; Tabassum, H.; Karakalos, S.; Feng, Z.; Wang, G. Atomically dispersed single Ni site catalysts for high-efficiency CO_2 electroreduction at industrial-level current densities. *Energy Environ. Sci.* **2022**, *15* (5), 2108-2119.

(25) Hu, C.; Hong, X.; Liu, M.; Shen, K.; Chen, L.; Li, Y. Hierarchically Ordered Pore Engineering of Carbon Supports with High-Density Edge-Type Single-Atom Sites to Boost Electrochemical CO_2 Reduction. *Adv. Mater.* **2024**, *36* (48), 2409531.

(26) Hossain, M. D.; Huang, Y.; Yu, T. H.; Goddard III, W. A.; Luo, Z. Reaction mechanism and kinetics for CO_2 reduction on nickel single atom catalysts from quantum mechanics. *Nat. Comm.* **2020**, *11* (1), 2256.

(27) Pei, Z.; Tan, H.; Gu, J.; Lu, L.; Zeng, X.; Zhang, T.; Wang, C.; Ding, L.; Cullen, P. J.; Chen, Z. A polymeric hydrogel electrocatalyst for direct water oxidation. *Nat. Commun.* **2023**, *14* (1), 818.

(28) Varela, A. S.; Ranjbar Sahraie, N.; Steinberg, J.; Ju, W.; Oh, H. S.; Strasser, P. Metal-doped nitrogenated carbon as an efficient catalyst for direct CO_2 electroreduction to CO and hydrocarbons. *Angew. Chem., Int. Ed.* **2015**, *54* (37), 10758-10762.

(29) Zhang, B.; Zhang, J.; Shi, J.; Tan, D.; Liu, L.; Zhang, F.; Lu, C.; Su, Z.; Tan, X.; Cheng, X. Manganese acting as a high-performance heterogeneous electrocatalyst in carbon dioxide reduction. *Nat. Comm.* **2019**, *10* (1), 2980.

(30) Zhang, C.; Yang, S.; Wu, J.; Liu, M.; Yazdi, S.; Ren, M.; Sha, J.; Zhong, J.; Nie, K.; Jalilov, A. S. Electrochemical CO_2 reduction with atomic iron-dispersed on nitrogen-doped graphene. *Adv. Energy Mater.* **2018**, *8* (19), 1703487.

(31) Mohd Adli, N.; Shan, W.; Hwang, S.; Samarakoon, W.; Karakalos, S.; Li, Y.; Cullen, D. A.; Su, D.; Feng, Z.; Wang, G. Engineering atomically dispersed FeN_4 active sites for CO_2 electroreduction. *Angew. Chem., Int. Ed.* **2021**, *60* (2), 1022-1032.

(32) Shen, L.; Wang, L.; Ye, Y.; Li, Z.; Dai, Q. Creation of intrinsic defects on ZIF-8 particles to facilitate electrochemical reduction of CO_2 over Fe single-atom catalyst. *Chem. Eng. J.* **2024**, *495*, 153073.

(33) Guan, A.; Fan, Y.; Xi, S.; Huang, H.; Zhang, Q.; Lyu, N.; Wu, B.; Chen, Y.; Liu, Z.; Yang, C. Nitrogen-adsorbed hydrogen species promote CO_2 methanation on Cu single-atom electrocatalyst. *ACS Mater. Lett.* **2022**, *5* (1), 19-26.

(34) Chen, Z.; Mou, K.; Yao, S.; Liu, L. Zinc-coordinated nitrogen-codoped graphene as an efficient catalyst for selective electrochemical reduction of CO_2 to CO. *ChemSusChem* **2018**, *11* (17), 2944-2952.

(35) Pei, J.; Yang, L.; Lin, J.; Zhang, Z.; Sun, Z.; Wang, D.; Chen, W. Integrating host design and tailored electronic effects of yolk–shell Zn− Mn diatomic sites for efficient CO_2 electroreduction. *Angew. Chem., Int. Ed.* **2024**, *136* (3), e202316123.

(36) Solovyev, I. I.; Dederichs, P. H.; Anisimov, V. V. Corrected atomic limit in the local-density approximation and the electronic structure of d impurities in Rb. *Phys. Rev. B* **1994**, *50* (23), 16861-16871.

(37) Lin, C. Y.; Zhang, L.; Zhao, Z.; Xia, Z. Design Principles for Covalent Organic Frameworks as Efficient Electrocatalysts in Clean Energy Conversion and Green Oxidizer Production. *Adv. Mater.* **2017**, *29* (17), 1606635.
